# Supplementary material for: NuRD‐independent Mi‐2 activity represses ectopic gene expression during neuronal maturation
Source: EMBO Rep. 2023 Feb 1;24(4):e55362. doi: 10.15252/embr.202255362 (PMC10074086; doi:10.15252/embr.202255362)
Supplement: Supplementary file 1 — Appendix S1 [file EMBR-24-e55362-s001.pdf]

NuRD independent Mi-2 activity represses ectopic gene expression during neuronal maturation.

Gabriel N Aughey‡, Elhana Forsberg†, Krista Grimes†, Shen Zhang†, Tony D Southall*

†Equal contribution

*Correspondence to t.southall@imperial.ac.uk

Department of Life Sciences, Imperial College London, Sir Ernst Chain Building, London, UK

‡Current address: Department of Clinical and Experimental Epilepsy, UCL Queen Square Institute of Neurology, London, United Kingdom.

Appendix information

Contents:

Appendix Figure S1. Tissue enrichment analysis for differentially upregulated genes associated with the "cilium organisation" GO term.

Appendix Figure S2. Expression of differentially expressed genes in other tissues.

Appendix Figure S3. Verification of *Mi-2* and *HDAC1* RNAi lines.

Appendix Figure S4. Pupal lethality in NuRD component RNAi knockdown experiments

Appendix Table S1. Fly stocks used.

Appendix Table S2. DNA oligos used in this study.

Appendix References

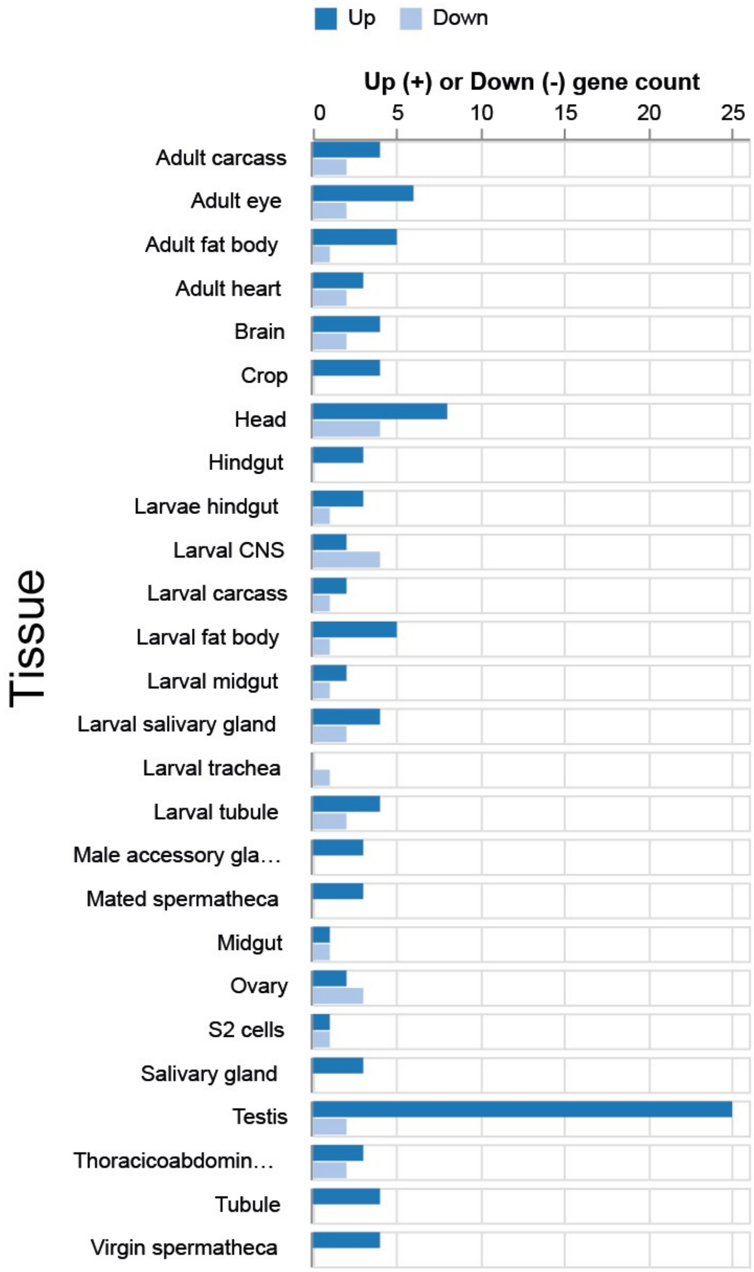

Appendix figure S1. Tissue enrichment analysis for differentially upregulated genes associated with the "cilium organisation" GO term. The majority of these genes are highly upregulated in testis. "Up" and "Down" refer to genes in the set that are significantly up or downregulated in the respective tissue.

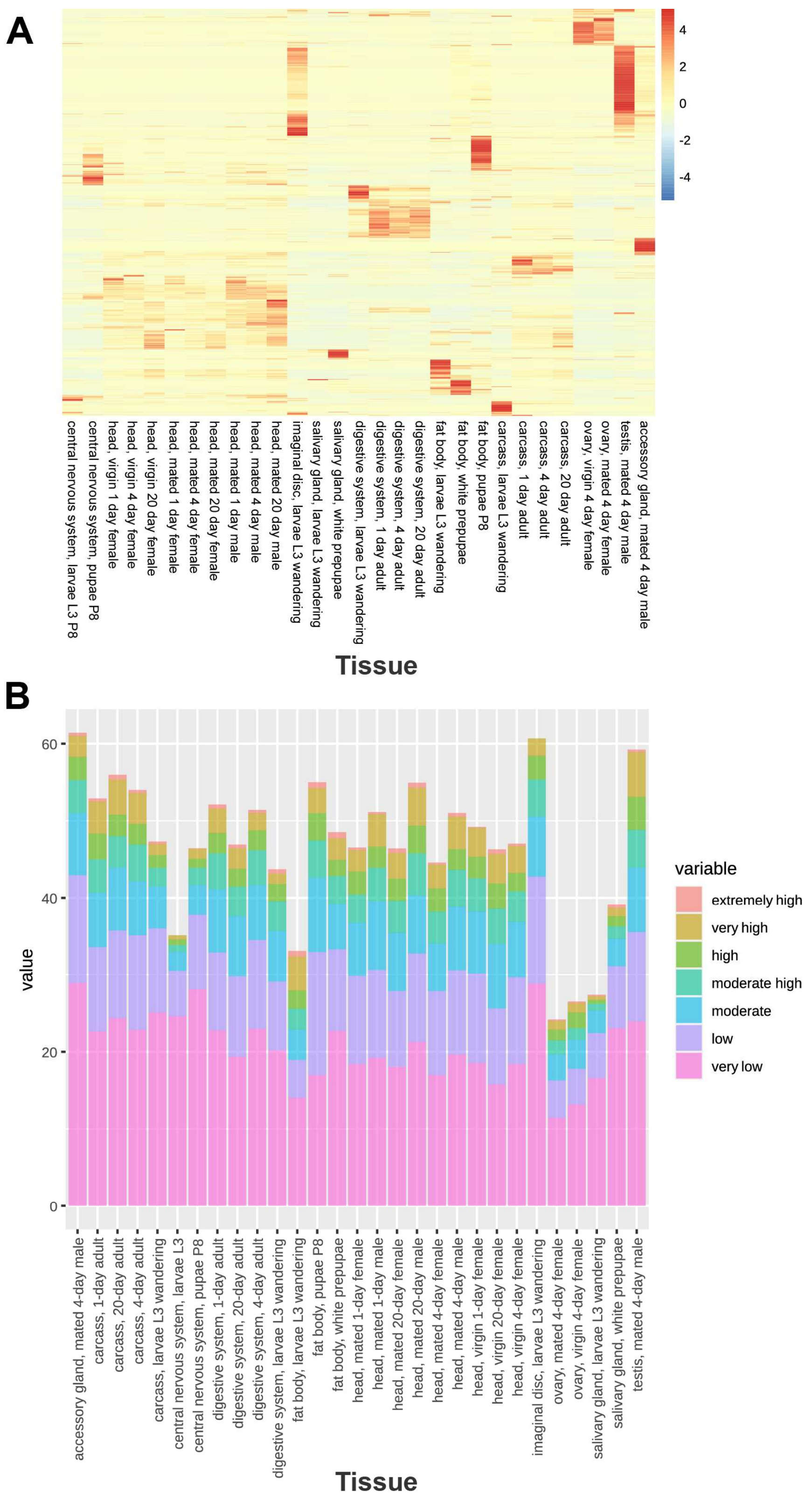

Appendix figure S2. Expression of differentially expressed genes in other tissues. A) Heatmap showing relative expression levels of upregulated genes across all tissues. B) Stacked bar plot showing percentage of upregulated genes that are expressed at different levels in specific tissues. Note that many upregulated genes are expressed at "very low" levels in the fly CNS, whilst upregulated genes are frequently expressed at "moderate" to very high levels in germline and other non-CNS tissues.

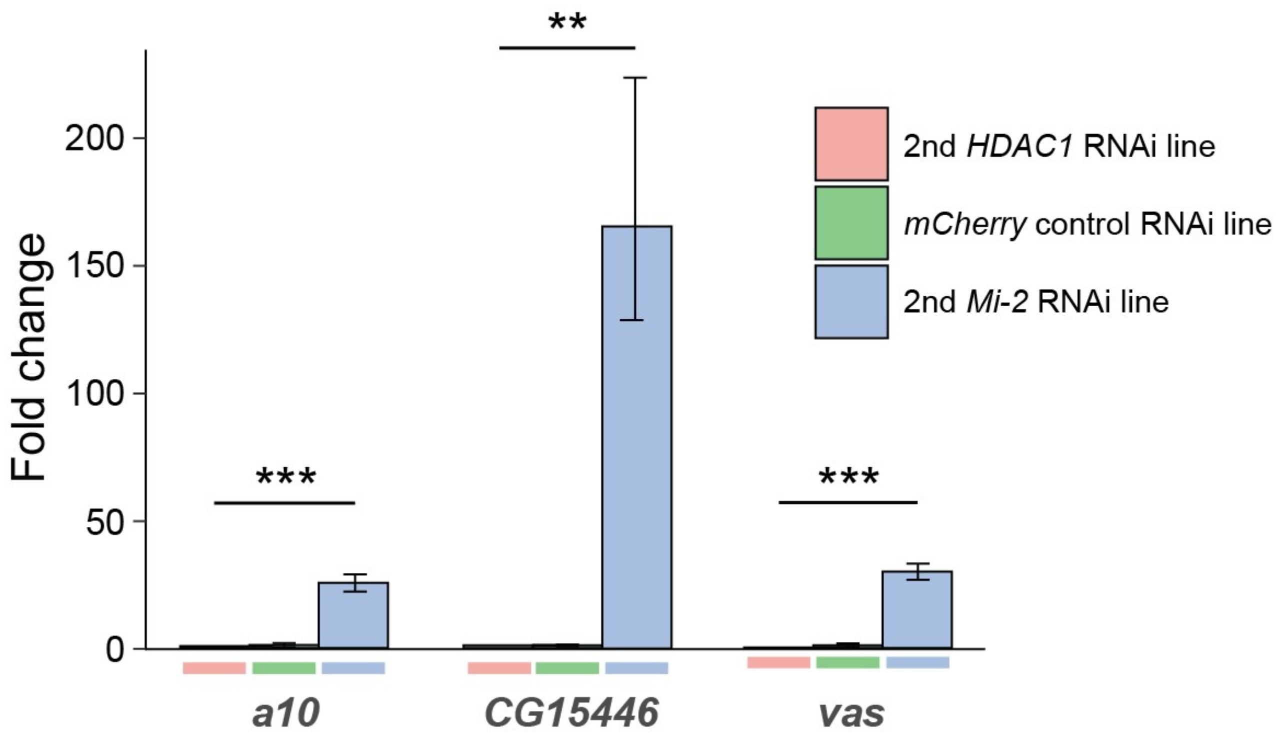

Appendix figure S3. Verification of *Mi-2* and *HDAC1* RNAi lines. qPCR measurement of non-neuronal genes in larval brains using alternative RNAi lines for *Mi-2* and *HDAC* (n = 3). These confirm the results obtained using the original RNAi lines. **P < 0.005, ***P < 0.0005 (one-tailed student's t-test). Error bars show standard deviation. Represented as mean ± SEM.

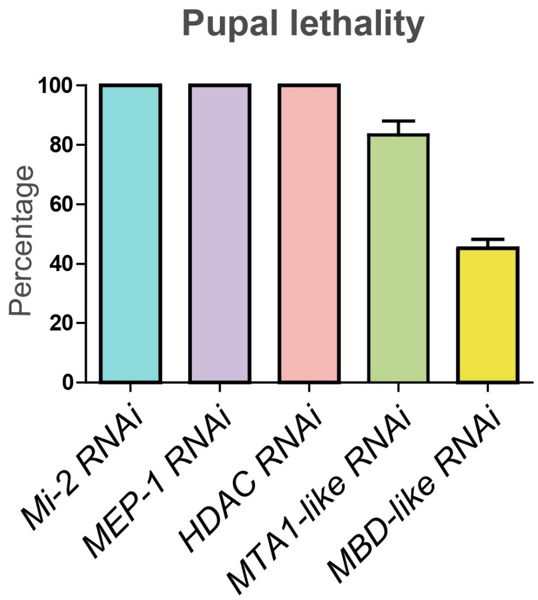

Appendix figure S4. Pupal lethality in NuRD component RNAi knockdown experiments. Percentage of pupae that die when NuRD subunits are knocked down in neurons (driven by *elav*-GAL4) during development. 3 independent experiments were performed for each genotype.

Appendix Table S1. Fly stocks used.

Genotype	Source	Reference	Description
y^1*w**; *Mi{PT-GFSTF.1}Mi-2*$^{MI07934-GFSTF.1}$ *Su(Tpl)*$^{MI07934-GFSTF.1-X}$*/TM6C, Sb*^{1} *Tb*^{1}	BDSC #63188	(Nagarkar-Jaiswal et al., 2015)	Mi-2 GFP trap
y^1 *w**; *Mi{PT-GFSTF.1}MTA1-like*$^{MI01790-GFSTF.1}$	BDSC #63161	(Nagarkar-Jaiswal et al., 2015)	MTA GFP trap
UAS-LT3-Dam	Andrea Brand	(Southall et al., 2013)	
UAS- Dam-MEP-1	This study		
UAS-Dam-Mi-2	This study		
UAS-MTA1-like-Dam	This study		flyORF TaDa
UAS-HDAC-Dam	This study		flyORF TaDa
wor-GAL4; *tub*-GAL80^{ts}	Andrea Brand	(Albertson et al., 2004)	
wor-GAL4	Andrea Brand	(Albertson et al., 2004)	
elav-GAL4*; tub*-GAL80^{ts}	Andrea Brand		
nSyb-GAL4	BDSC #51941		
hs-flp; UAS-flyORF.TaDa	BDSC #91637	(Aughey et al., 2021)	
UAS-HDAC1	FlyORF F000675	(Bischof et al., 2013)	
UAS-MTA1-like	FlyORF F001892	(Bischof et al., 2013)	
UAS-Mi2 RNAi	BDSC #51774		
UAS-Mi2 RNAi (2nd line)	BDSC #35398		
UAS-MTA1-like RNAi	BDSC #34624		
UAS-HDAC1 RNAi	BDSC #33725		
UAS-HDAC1 RNAi (2nd line)	BDSC #34846		
UAS-MEP-1 RNAi	BDSC #62180	(Perkins et al., 2015)	
UAS-MBD-like RNAi	VDRC #9261	(Dietzl et al., 2007)	
mCherry-RNAi (control)	BDSC #35787		
repo-GAL80	Manolis Fanto	(Awasaki et al., 2008)	
elav-GAL4 ; *repo*-GAL80 / *TM6*	This study		

Appendix Table S2. DNA oligos used in this study.

Primer name	Primer sequence
vas qRT-PCR forward	TGTCTGACGACTGGGATGATG
vas qRT-PCR reverse	ATTTCCTCCTTGGTAGCCGC
CG17566 qRT-PCR forward	TTGGGCCAATGTGGCAATCA
CG17566 qRT-PCR reverse	TGCGATCCTGTCCATCGGT
qin qRT-PCR forward	TCCCTTTCTACTGGGATGCG
qin qRT-PCR reverse	GAGCTGGACTATGGCACACG
RpS5b qRT-PCR forward	ACTACATTGCCGTAAAGGAGAAG
RpS5b qRT-PCR reverse	CATTGGGCCTTGCGGAATC
CG8526 qRT-PCR forward	GATTTGATTGAGTTCTGCCCACT
CG8526 qRT-PCR reverse	CTTGTATGTCTTTCCCACTTCGT
CG15446 qRT-PCR forward	CGGAAACGGCTACCCATGT
CG15446 qRT-PCR reverse	CCCCGACTTACCTTCATCTTCG
a10 qRT-PCR forward	ATCCTTAACCAAGAGCGACTGT
a10 qRT-PCR reverse	TCACCTTTTCAGCACCATACC
RpL4 qRT-PCR forward	TCCACCTTGAAGAAGGGCTA
RpL4 qRT-PCR reverse	TTGCGGATCTCCTCAGACTT
*Dam-Mi2*_forward	gaagaggatctggccggcgcagatctgcggATGGCATCGGAGGAAGAGAATGACGATAAT
*Dam-Mi2*_reverse	aagtaaggttccttcacaaagatcctctagCTAGACGCCGGAATTATTCGATAGCTGGCC
*Dam-MEP-1*_forward	gaagaggatctggccggcgcagatctgcggATGACTGAAGTTGATGTCGTTTTGCCGGAG
*Dam-MEP-1*_reverse	aagtaaggttccttcacaaagatcctctagTTAATCTATGACATGACTCTCCATATTTG

Appendix References

Albertson, R., Chabu, C., Sheehan, A., and Doe, C.Q. (2004). Scribble protein domain mapping reveals a multistep localization mechanism and domains necessary for establishing cortical polarity. *J Cell Sci* 117, 6061-6070.

Aughey, G.N., Delandre, C., McMullen, J.P.D., Southall, T.D., and Marshall, O.J. (2021). FlyORF-TaDa allows rapid generation of new lines for in vivo cell-type-specific profiling of protein-DNA interactions in Drosophila melanogaster. *G3 (Bethesda)* 11.

Awasaki, T., Lai, S.L., Ito, K., and Lee, T. (2008). Organization and postembryonic development of glial cells in the adult central brain of Drosophila. *J Neurosci* 28, 13742-13753.

Bischof, J., Bjorklund, M., Furger, E., Schertel, C., Taipale, J., and Basler, K. (2013). A versatile platform for creating a comprehensive UAS-ORFeome library in Drosophila. *Development* 140, 2434-2442.

Dietzl, G., Chen, D., Schnorrer, F., Su, K.C., Barinova, Y., Fellner, M., Gasser, B., Kinsey, K., Oppel, S., Scheiblauer, S., *et al.* (2007). A genome-wide transgenic RNAi library for conditional gene inactivation in Drosophila. *Nature* 448, 151-156.

Nagarkar-Jaiswal, S., Lee, P.T., Campbell, M.E., Chen, K., Anguiano-Zarate, S., Gutierrez, M.C., Busby, T., Lin, W.W., He, Y., Schulze, K.L., *et al.* (2015). A library of MiMICs allows tagging of genes and reversible, spatial and temporal knockdown of proteins in Drosophila. *Elife* 4.

Perkins, L.A., Holderbaum, L., Tao, R., Hu, Y., Sopko, R., McCall, K., Yang-Zhou, D., Flockhart, I., Binari, R., Shim, H.S., *et al.* (2015). The Transgenic RNAi Project at Harvard Medical School: Resources and Validation. *Genetics* 201, 843-852.

Southall, T.D., Gold, K.S., Egger, B., Davidson, C.M., Caygill, E.E., Marshall, O.J., and Brand, A.H. (2013). Cell-type-specific profiling of gene expression and chromatin binding without cell isolation: assaying RNA Pol II occupancy in neural stem cells. *Dev Cell* 26, 101-112.
