# Supplementary material for: NuRD‐independent Mi‐2 activity represses ectopic gene expression during neuronal maturation
Source: EMBO Rep. 2023 Feb 1;24(4):e55362. doi: 10.15252/embr.202255362 (PMC10074086; doi:10.15252/embr.202255362)
Supplement: Supplementary file 2 — Expanded View Figures PDF [file EMBR-24-e55362-s004.pdf]

Expanded View Figures

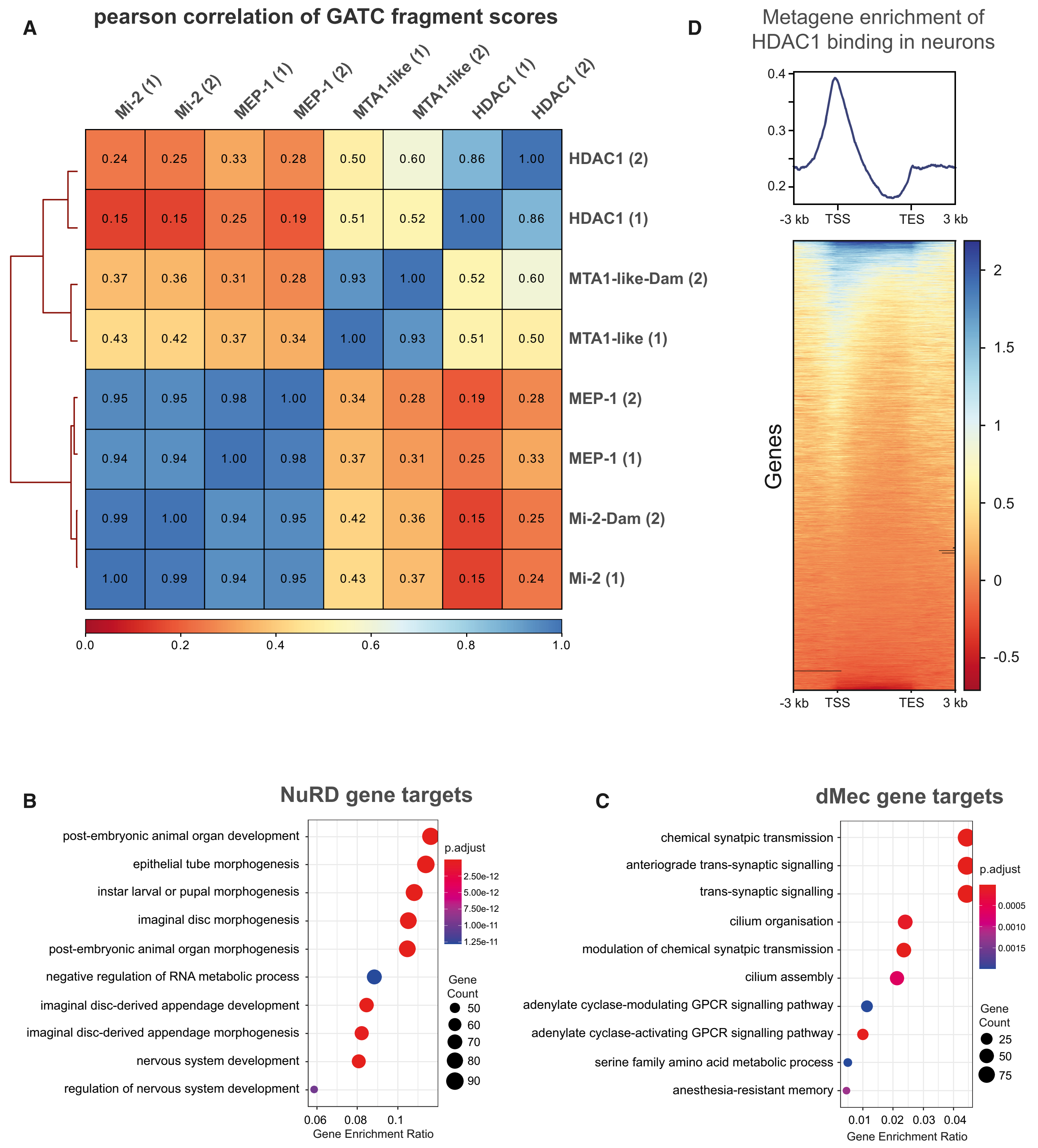

Figure EV1.

Figure EV1. Genomic binding of NuRD components.

A Correlation between NuRD subunits (including replicates) in larval neurons (*elav*-GAL4). Very strong correlations are seen between Mi-2 and MEP-1 binding while the correlation between Mi-2/MEP-1 and either MTA-1like or HDAC1 is relatively lower. Good correlations are exhibited between replicates for each subunit (spearman's correlation $r^2 = 0.85–0.95$).

B GO analysis of NuRD gene targets.

C GO analysis of dMec gene targets genes.

D Metagene analysis of HDAC1 binding. TSS is Transcriptional Start Site, and TES is Transcriptional End Site.

Figure EV2. RNA-seq quality control and differentially expressed genes.

A Heatmap showing Pearson correlation of RNA-seq reads between biological replicates and control and experimental groups. Very strong correlations are observed between replicates ($R^2 > 0.97$) with slightly lower correlations between knockdown and controls reflecting the changes in *Mi-2* RNAi transcriptome.

B Genome browser tracks indicating RNA-seq read coverage at the *Mi-2* locus. *Mi-2* reads are clearly depleted in *Mi-2 RNAi* compared with *elav*-GAL4 × *mCherry RNAi* controls (adjusted P value $< 2.2 \times 10^{-227}$).

C Heatmap showing relative changes in normalised gene expression for the thirty most significantly upregulated genes.

D Heatmap showing relative changes in normalised gene expression for the 30 most significantly down-regulated genes.

E, F Read coverage for (E) *gammaTub37C* and (F) *RpS5b* loci, which are non-CNS genes ectopically expressed in *Mi-2* knockdown.

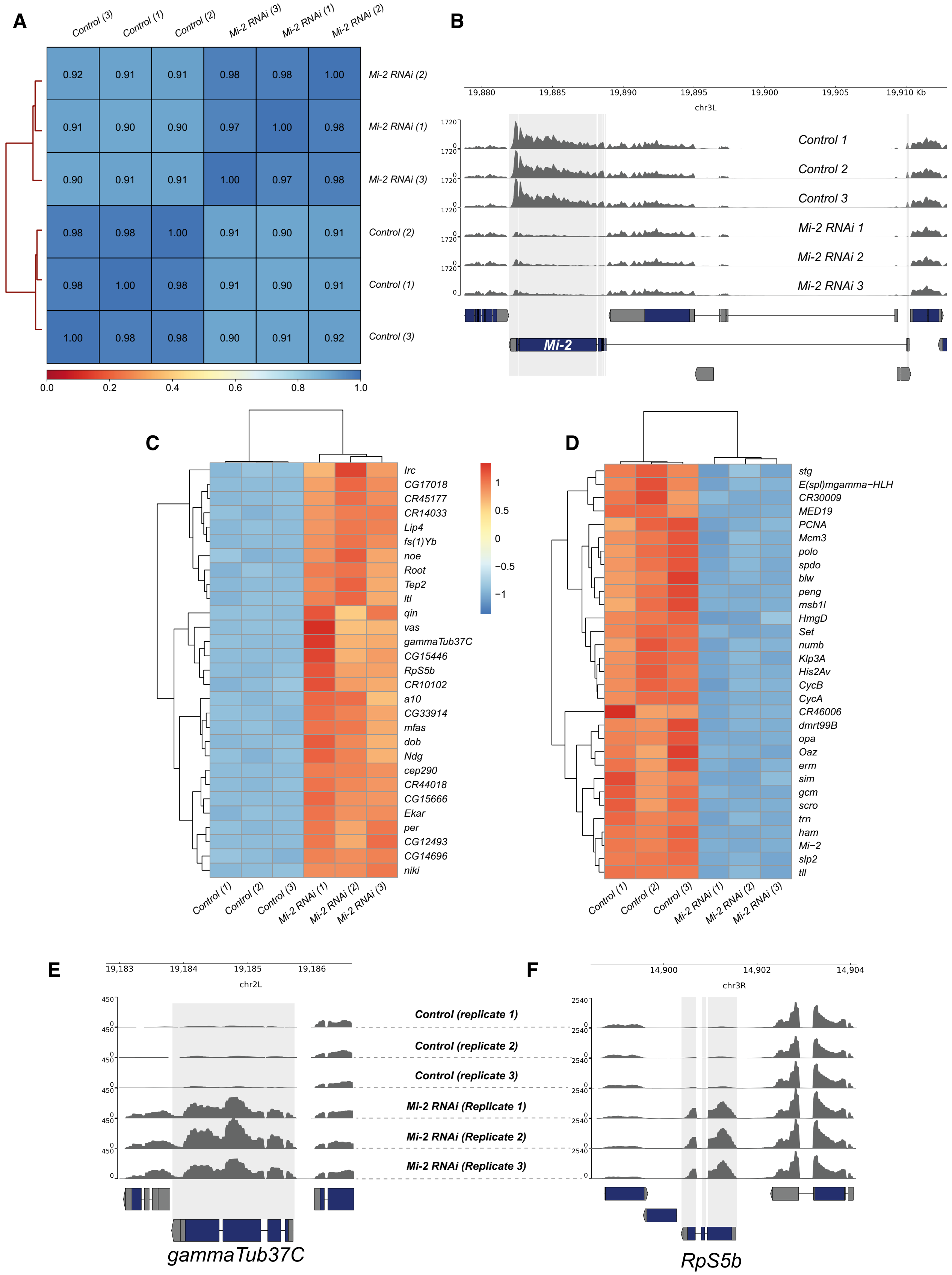
A
Control (3)
Control (1)
Control (2)
Mi-2 RNAi (3)
Mi-2 RNAi (1)
Mi-2 RNAi (2)
B
chr3L
Control 1
Control 2
Control 3
Mi-2 RNAi 1
Mi-2 RNAi 2
Mi-2 RNAi 3
Mi-2
C
D
E
chr2L
Control (replicate 1)
Control (replicate 2)
Control (replicate 3)
Mi-2 RNAi (Replicate 1)
Mi-2 RNAi (Replicate 2)
Mi-2 RNAi (Replicate 3)
gammaTub37C
F
chr3R
RpS5b

Figure EV2.

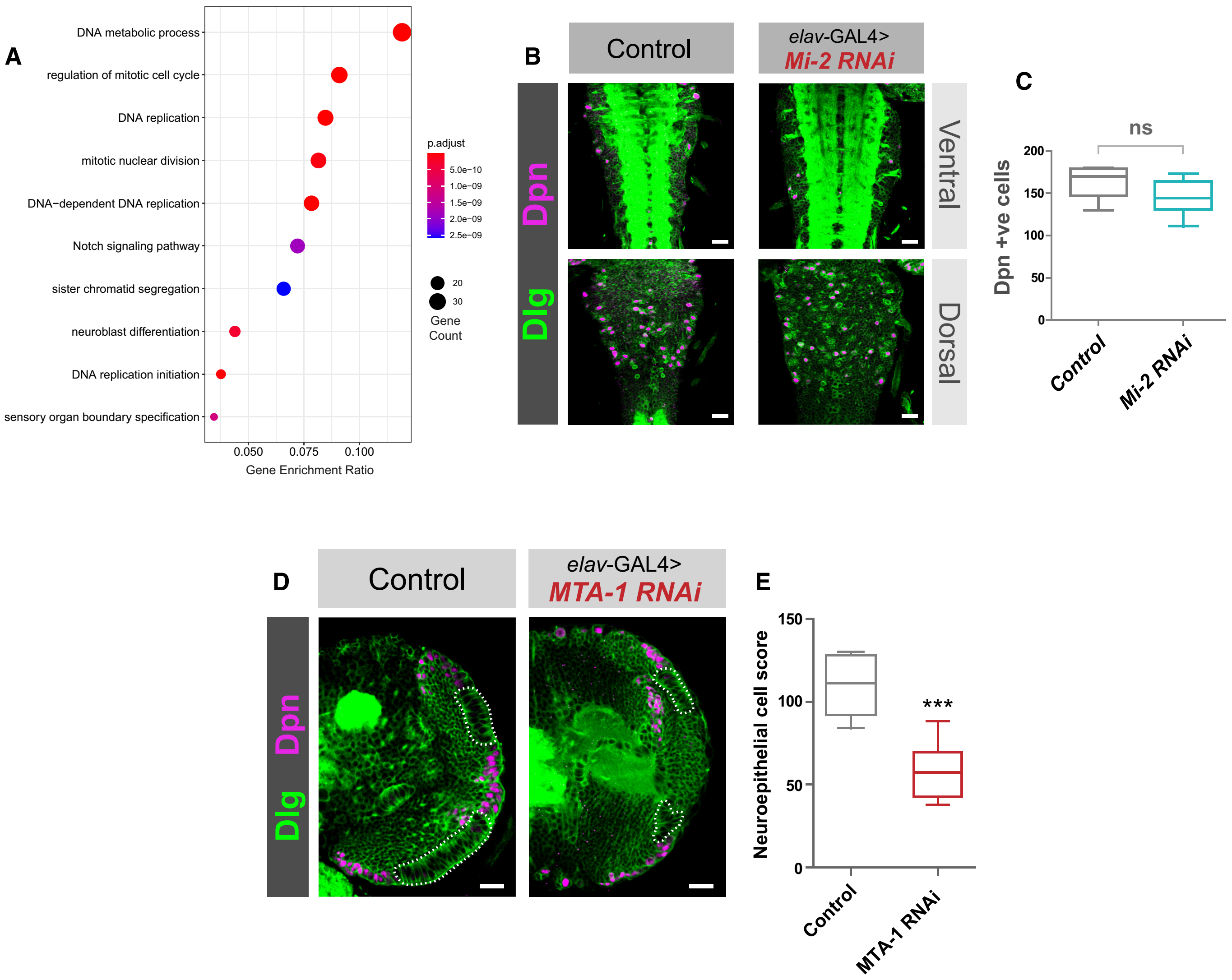

Figure EV3. *NuRD* knockdown larval brain phenotypes.

A GO analysis of downregulated genes.

B *Mi-2* knockdown does not affect NSC numbers in the VNC. (B) Example images from ventral and dorsal sections of the VNC. Scale bars = 20 µm.

C Quantification of Dpn-positive cells (no significant difference). Six VNCs measured for each genotype. No significant difference (two-tailed student's *t*-test). Represented as a box plot.

D *MTA1-like* knockdown causes an optic lobe phenotype. (D) Representative images of control (*elav*-GAL4;; *mCherry RNAi*), and *MTA1-like* knockdown in the third instar larval CNS (all scale bars = 20 µm). Discs large (Dlg) = green, Deadpan (Dpn) = magenta. Neuroepithelial cells are highlighted by dashed lines.

E Quantification of optic lobe neuroepithelial cells of genotypes shown in panel A. At least eight brains measured for each genotype. ***$P < 0.001$ (one-tailed student's *t*-test). Represented as a box plot.

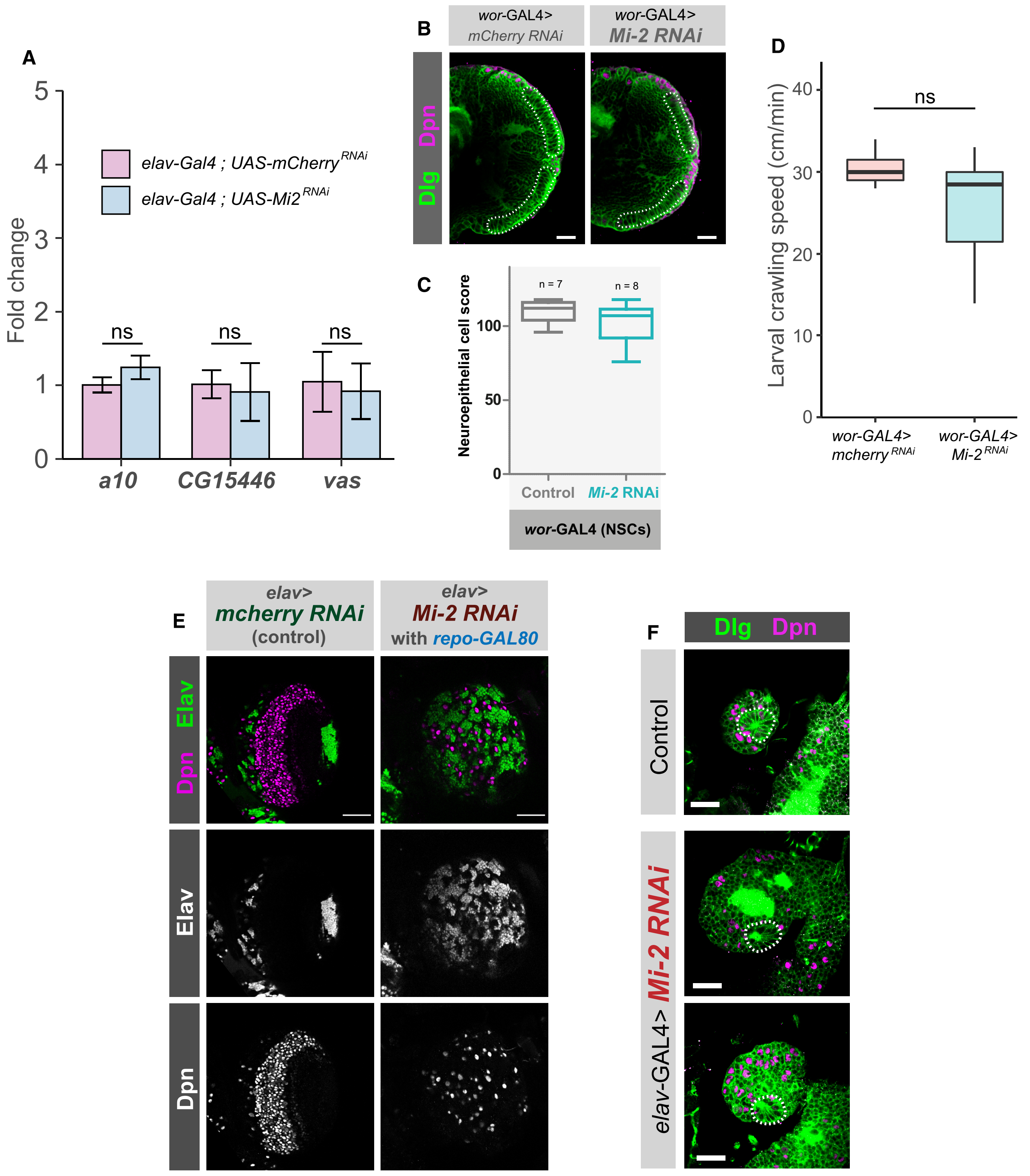
A
Fold change
elav-Gal4 ; UAS-mCherry RNAi
elav-Gal4 ; UAS-Mi2 RNAi
ns
a10
CG15446
vas
B
wor-GAL4> mCherry RNAi
wor-GAL4> Mi-2 RNAi
Dlg Dpn
C
Neuroepithelial cell score
n = 7
n = 8
Control
Mi-2 RNAi
wor-GAL4 (NSCs)
D
Larval crawling speed (cm/min)
ns
wor-GAL4> mcherry RNAi
wor-GAL4> Mi-2 RNAi
E
elav> mcherry RNAi (control)
elav> Mi-2 RNAi with repo-GAL80
Dpn Elav
Elav
Dpn
F
Dlg Dpn
Control
elav-GAL4> Mi-2 RNAi

Figure EV4.

Figure EV4. Differential requirements of Mi-2 during neuronal differentiation.

A Knockdown of *Mi-2* in adult brain neurons does not cause ectopic expression of genes. qPCR measurement of gene expression in adult heads. *Mi-2*^{RNAi} is induced for 24 h using GAL80^{ts}. No significant changes in expression of either *vas*, *a10* or *CG15446* were observed (one-tailed students *t*-test, represented as mean ± SEM.). Three biological replicates per genotype.
B Representative images of optic lobes for control and *Mi-2* knockdown in NSCs.
C Quantification of optic lobe neuroepithelial cells of genotypes shown in panel B (*n* >= 7 for each group). Represented as a box plot. No significant difference found (one-tailed student's *t*-test).
D Larval crawl speed (cm/min) in *wor*-GAL4 × *Mi-2* RNAi and *wor*-GAL4 × *mCherry* RNAi controls. No significant changes in crawl speed were observed (two-tailed students *t*-test, *n* = 10 animals per genotype). Represented as a box plot.
E Optic lobe phenotype is not due to *Mi-2* knockdown in glial cells. Optic lobe of third instar larvae in which *repo*-GAL80 represses glial GAL4 expression. Loss of neuroepithelial cells is still observed as in *elav*-GAL4; *Mi-2 RNAi* brains. Scale bar = 50 µm.
F Characteristic rosette structure of the neuroepithelial cells is present in the control first instar optic lobe and in *elav*-GAL4; *Mi-2 RNAi* 1st instar optic lobes. Scale bar = 20 µm.

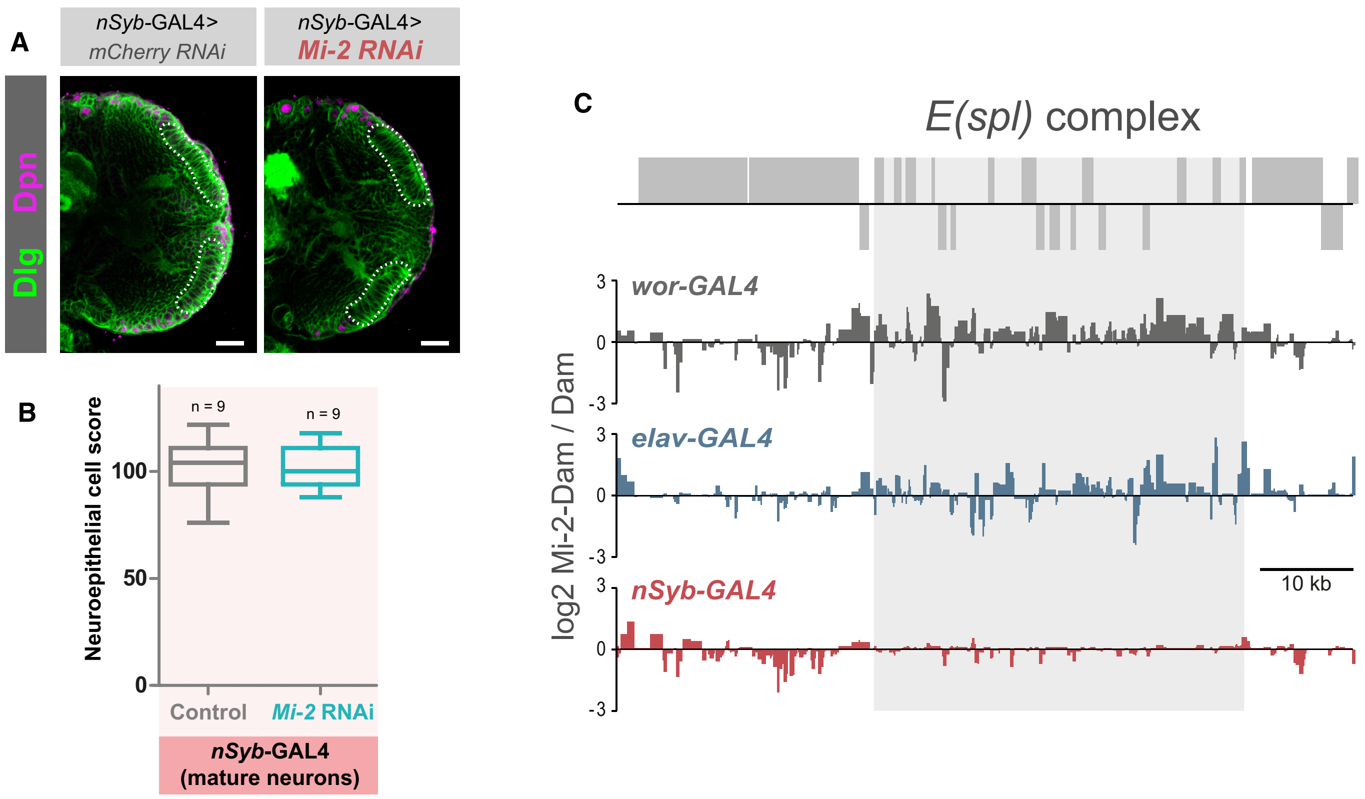

Figure EV5. Differential requirements and binding of Mi-2 during neuronal differentiation.

A Representative images of optic lobes for control and *Mi-2* knockdown in mature neurons.
B Quantification of optic lobe neuroepithelial cells of genotypes shown in panel B (*n* >= 7 for each group). Represented as a box plot. No significant difference found (one-tailed student's *t*-test).
C Mi-2 binding at the *E(spl)* complex is lost in mature neurons. Grey boxes represent genes.
