# Supplementary material for: NuRD‐independent Mi‐2 activity represses ectopic gene expression during neuronal maturation
Source: EMBO Rep. 2023 Feb 1;24(4):e55362. doi: 10.15252/embr.202255362 (PMC10074086; doi:10.15252/embr.202255362)
Supplement: Supplementary file 3 — PDF+ [file EMBR-24-e55362-s002.pdf]

Article

TRANSPARENT PROCESS
OPEN ACCESS

EMBO reports

NuRD-independent Mi-2 activity represses ectopic gene expression during neuronal maturation

Gabriel N Aughey‡, Elhana Forsberg†, Krista Grimes†, Shen Zhang† & Tony D Southall*

Abstract

During neuronal development, extensive changes to chromatin states occur to regulate lineage-specific gene expression. The molecular factors underlying the repression of non-neuronal genes in differentiated neurons are poorly characterised. The Mi2/NuRD complex is a multiprotein complex with nucleosome remodelling and histone deacetylase activity. Whilst NuRD has previously been implicated in the development of nervous system tissues, the precise nature of the gene expression programmes that it coordinates is ill-defined. Furthermore, evidence from several species suggests that Mi-2 may be incorporated into multiple complexes that may not possess histone deacetylase activity. We show that Mi-2 activity is required for suppressing ectopic expression of germline genes in neurons independently of HDAC1/NuRD, whilst components of NuRD, including Mi-2, regulate neural gene expression to ensure proper development of the larval nervous system. We find that Mi-2 binding in the genome is dynamic during neuronal maturation, and Mi-2-mediated repression of ectopic gene expression is restricted to the early stages of neuronal development, indicating that Mi-2/NuRD is required for establishing stable neuronal transcriptomes during the early stages of neuronal differentiation.

Keywords chromatin; differentiation; *Drosophila*; neurons; NuRD
Subject Categories Chromatin, Transcription & Genomics; Development; Neuroscience
DOI 10.15252/embr.202255362 | Received 6 May 2022 | Revised 10 January 2023 | Accepted 16 January 2023 | Published online 1 February 2023
EMBO Reports (2023) 24: e55362

Introduction

Neuronal development involves the transition of multipotent precursor cells into highly specialised neurons with distinct morphological and biochemical characteristics. Extensive gene expression changes accompany the differentiation of precursors into specialised cell-types. These gene expression changes are underpinned by a dynamic chromatin environment in which regulation of nucleosome positioning and histone modifications ensures appropriate transcriptional responses. Whilst recent studies have provided illuminating descriptions of changes to chromatin accessibility and histone modifications during the process of neuronal differentiation (Aughey *et al*, 2018; Preissl *et al*, 2018; Ziffra *et al*, 2021; Janssens *et al*, 2022), the chromatin-interacting complexes that affect these changes are not well defined.

The Mi-2/NuRD complex is a multiprotein chromatin remodelling complex that influences gene expression via two separate enzymatic activities; ATP-dependent nucleosome remodelling and histone deacetylation (Tong *et al*, 1998; Wade *et al*, 1998; Xue *et al*, 1998; Zhang *et al*, 1998). These activities are mediated by enzymes found in two distinct subunits. The nucleosome remodelling subunit, Mi-2, is a well-conserved ATP-dependent nucleosome remodeller (CHD4 in mammals). While histone deacetylation is conferred by a separate HDAC (histone deacetylase) enzyme. NuRD components are widely conserved and broadly expressed in most metazoan tissues, reflecting the important role of the complex in gene regulation. NuRD has been reported to have critical roles in developmental processes (e.g. in muscle (Gomez-Del Arco *et al*, 2016) and haematopoietic (Yoshida *et al*, 2008) cell differentiation), as well as regulation of pluripotent cell reprogramming (Rais *et al*, 2013).

Mi-2/NuRD complexes have critical roles in neurodevelopment and have been implicated in the aetiology of neurodevelopmental disorders (Hoffmann & Spengler, 2019). A recent study showed that Mi-2 is required in *Drosophila* neuronal lineages to deactivate notch-responsive stem cell enhancers in neuronal progeny, thereby ensuring the fidelity of differentiated cell fate in neurons (Zacharioudaki *et al*, 2019). Similarly, NuRD was shown to deactivate gene expression by acting on promoters of presynaptic genes to promote synaptic connectivity (Yamada *et al*, 2014). These studies suggest a predominantly suppressive role for NuRD in the regulation of gene expression. Whilst this is consistent with early models of NuRD activity as a repressive complex, more recent studies have presented a more nuanced role for NuRD in modulating gene expression, which may be neither activating or repressive, but acting to fine-tune gene expression and reduce transcriptional noise (Bornelov *et al*, 2018; Burgold *et al*, 2019; Ragheb *et al*, 2020). Therefore, an important unanswered question is whether NuRD is primarily repressive in neuronal development, or whether it may facilitate control of active gene expression as demonstrated in stem cell models.

Department of Life Sciences, Imperial College London, London, UK
*Corresponding author. Tel: +44 020 45338; E-mail: t.southall@imperial.ac.uk
†These authors contributed equally to this work
‡Present address: Department of Clinical and Experimental Epilepsy, UCL Queen Square Institute of Neurology, London, UK

© 2023 The Authors. Published under the terms of the CC BY 4.0 license

NuRD composition is dynamic in mouse cortical development, with alternate Mi-2/CHD paralogues incorporated to mediate distinct effects in different developmental stages (Nitarska *et al*, 2016). In *Drosophila*, the components of the NuRD complex are well conserved (although only single homologues of most NuRD proteins are identified). However, despite the strong conservation of NuRD constituent proteins, and ample evidence for physical interactions between them (Reddy *et al*, 2010; Zhang *et al*, 2016), the extent to which the canonical NuRD complex regulates gene expression in *Drosophila* is uncertain. A separate complex consisting of only Mi2 and MEP-1 (termed dMec) has been suggested to be more prevalent than NuRD in fly and may be responsible for the majority of Mi-2-dependent phenotypes (Kunert *et al*, 2009). This observation has not been corroborated by further studies; however, NuRD-independent roles for Mi-2 may be present in human cells (Amaya *et al*, 2013). Alternatively, it is possible that since NuRD comprises pre-assembled modules (Zhang *et al*, 2016), and Mi-2 may exist as a peripheral component to the core complex (Low *et al*, 2016), the detection of non-NuRD associated Mi-2 may simply be a proteomic artefact that does not reflect a *bona fide* biological activity. Therefore, the functional role of dMEC, and its relationship to the canonical NuRD holocomplex, remains poorly characterised.

Here, we set out to better understand the influence of Mi-2/NuRD on gene expression in the development of the central nervous system. Genomic profiling with Targeted DamID revealed distinct binding of Mi-2 containing complexes, NuRD and dMEC. Knockdown of Mi-2 in larval neurons resulted in ectopic expression non-neuronal genes as well as defective optic lobe development. Mi-2 represses lineage-inappropriate gene expression independently of NuRD, whilst optic lobe phenotypes are phenocopied by other members of the NuRD complex. We find that Mi-2 is not required for repression of non-neuronal genes after the early stages of differentiation, indicating that Mi-2 activity is critical for ensuring maintenance of the neuronal gene expression programme during maturation, but alternative mechanisms may be employed in fully differentiated neurons.

Results

Mi-2 is required in neurons for survival and larval locomotion

Mi-2 has been shown to protect neural lineages from mitogenic notch signalling, indicating that NuRD has an important role in gene regulation in the neuroblasts and early progeny of *Drosophila* central nervous system (CNS) lineages. To better understand NuRD function in the fly CNS, we set out to examine the distribution of the core ATP-dependent nucleosome remodelling subunit Mi-2. We utilised a MiMIC insertion line in which Mi-2 is EGFP-tagged at its endogenous locus (*Mi-2-GFP* hereafter). We observed widespread Mi-2 expression with detectable EGFP signal in multiple cell types in the third instar CNS (Fig 1A). EGFP signal was visible in nuclei of neural stem cells (NSCs; as distinguished by their larger size and absence of Elav or Repo) as well as all neurons and some glia (Fig 1A). The nuclear expression of Mi-2 in progenitor and post-mitotic cell-types in the larval CNS suggests that Mi-2 activity is likely to be involved in gene regulation in all stages of neuronal differentiation, including differentiated neurons.

To understand the importance of *Mi-2* expression in neurons, we used RNAi interference (RNAi) to induce cell-type-specific knockdown of *Mi-2* in the CNS. An RNAi line targeting Mi-2 was previously shown to produce robust knockdown of Mi-2 in neuronal lineages; therefore, we decided to utilise this line for our study (Zacharioudaki *et al*, 2019). Using the pan-neuronal *elav*-GAL4 driver, we observed lethality during late larval/pupal stages (Fig 1B). Whilst neuronal *Mi-2* appeared dispensable for embryogenesis and larval survival (possibly due to maternal contribution of Mi-2), we observed a severe mobility defect in larval crawling compared with controls (Fig 1C) indicating that *Mi-2* activity in neurons is required throughout development at all life stages for normal neuronal function.

Mi-2 association with chromatin reveals the presence of distinct complexes

Our data, along with previous reports, suggest that Mi-2/NuRD has an essential role in the regulation of neuronal gene expression. However, it is unclear which genes Mi-2 regulates and whether its function is predominantly via NuRD or dMEC (Mi-2 and MEP-1 only). To better understand NuRD function in regulating neuronal gene expression, we profiled the genomic binding of NuRD complex components using Targeted DamID (TaDa; Southall *et al*, 2013). This approach involves fusing a protein of interest to Dam methylase and expressing in a defined cell population, followed by enrichment and sequencing of selectively methylated regions, which reflect loci at which the protein associates with DNA (reviewed in Aughey *et al*, 2019).

We used Targeted DamID to profile genomic association of Mi-2 as well as NuRD components MTA1-like and HDAC1 in neurons (defined by *elav*-GAL4 expression) in the larval CNS. MTA1-like is a core component of the NuRD histone deacetylase subcomplex, with cells lacking MTA orthologues in mammals considered to be effectively NuRD-null (Burgold *et al*, 2019), whilst HDAC1 is the histone deacetylase subunit (also known as Rpd3). We also assayed for genomic binding of MEP-1, previously reported to associate exclusively with Mi-2 as part of both the dMEC and NuRD complexes. Principal component analysis revealed a single cluster including both Mi-2 and MEP-1 replicates, whilst the remaining NuRD components, MTA1-like and HDAC1, appeared to be distinct (Fig 2A–C). Similarity between Mi-2 and MEP-1 binding is further supported by the close correlation between the two datasets (pearson $R^2 = 0.94/0.95$), which is almost as strong as that seen between replicates (Fig EV1A). In contrast, both MTA-1like and HDAC1 showed relatively poor correlations to MEP-1 or Mi-2, whereas their correlation with each other was slightly stronger. Whilst HDAC1 binding may be expected to be poorly correlated with Mi-2 due to the inclusion of HDAC1 in protein complexes other than NuRD (e.g. with Polycomb Group proteins (Tie *et al*, 2001)), the relatively low correlation with MTA1-like points towards the possibility of distinct Mi-2 containing complexes having distinct binding in neurons.

To further understand the relationship between Mi-2 and other NuRD components at the chromatin level, Mi-2 significantly bound peaks were compared with those of MEP-1, MTA1-like and HDAC1. We found that the majority of MTA1-like peaks coincided with Mi-2, although a significant number of Mi-2 peaks did not overlap with MTA1-like (Fig 2C). We observed strong binding of HDAC1

 © 2023 The Authors

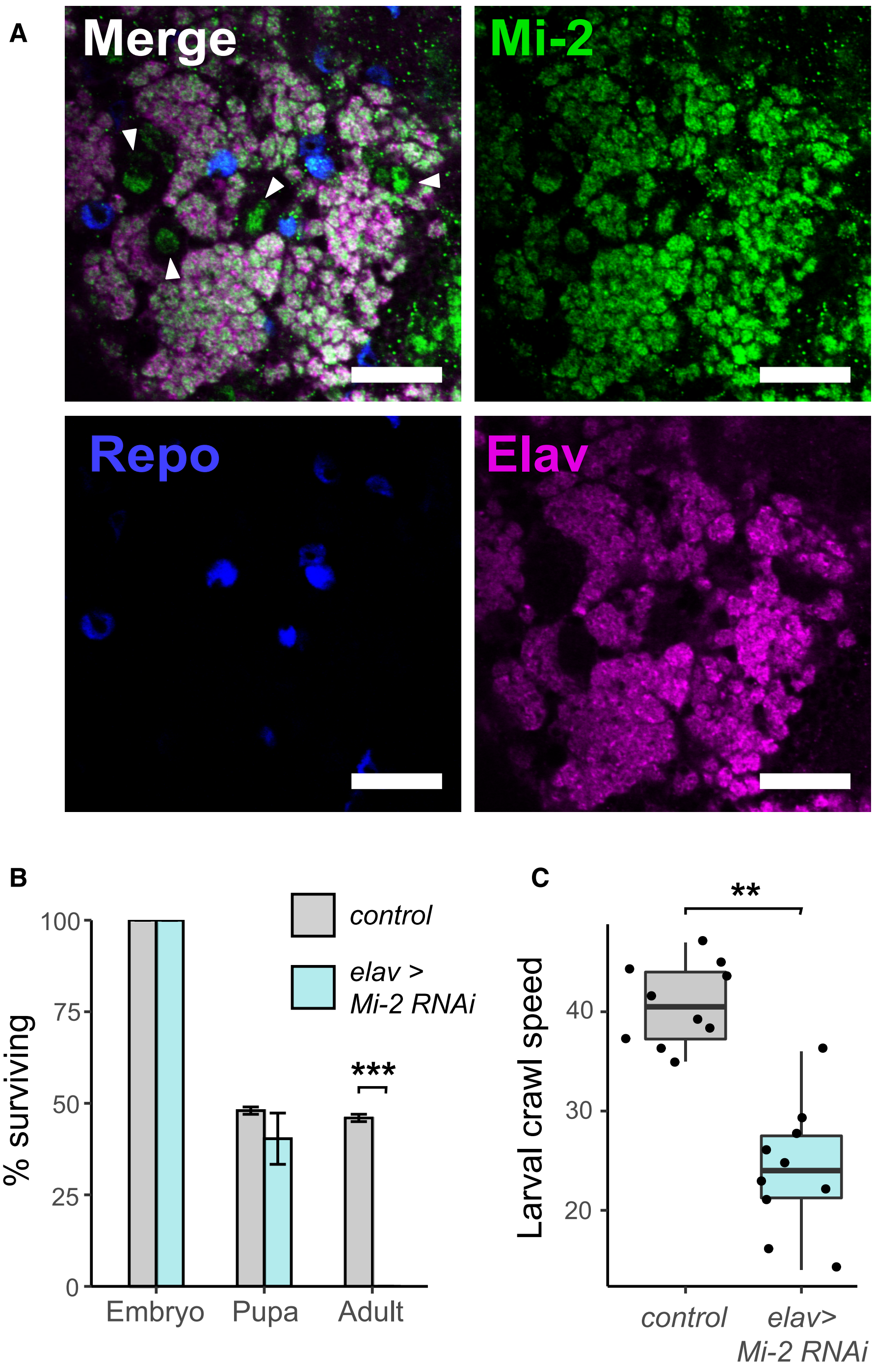

Figure 1. Critical role of *Mi-2* RNAi in *Drosophila* neurons.

A Third instar larval CNS of *Mi-2* GFP-trap line (scale bar = 20 μm). White arrows highlight NSC nuclei.

B Survival of *Mi-2* RNAi and control (*mCherry-RNAi*) flies during development (***$P < 0.001$).

C Larval crawl speed (cm/min) in *Mi-2* RNAi and *mCherry* RNAi controls (**$P < 0.01$).

throughout the genome; however, a relatively low proportion of HDAC1 peaks intersected with Mi-2 peaks (Fig 2B and C), indicating that Mi-2 is found in association with chromatin without HDAC1 or MTA1-like at the majority of loci.

We observed that around half of Mi-2 peaks coincided with either MTA1-like or HDAC1 with fewer still having all three detected at the same site (around a quarter of total Mi-2 peaks). We also saw that a smaller proportion of HDAC1 peaks were shared with Mi-2

© 2023 The Authors

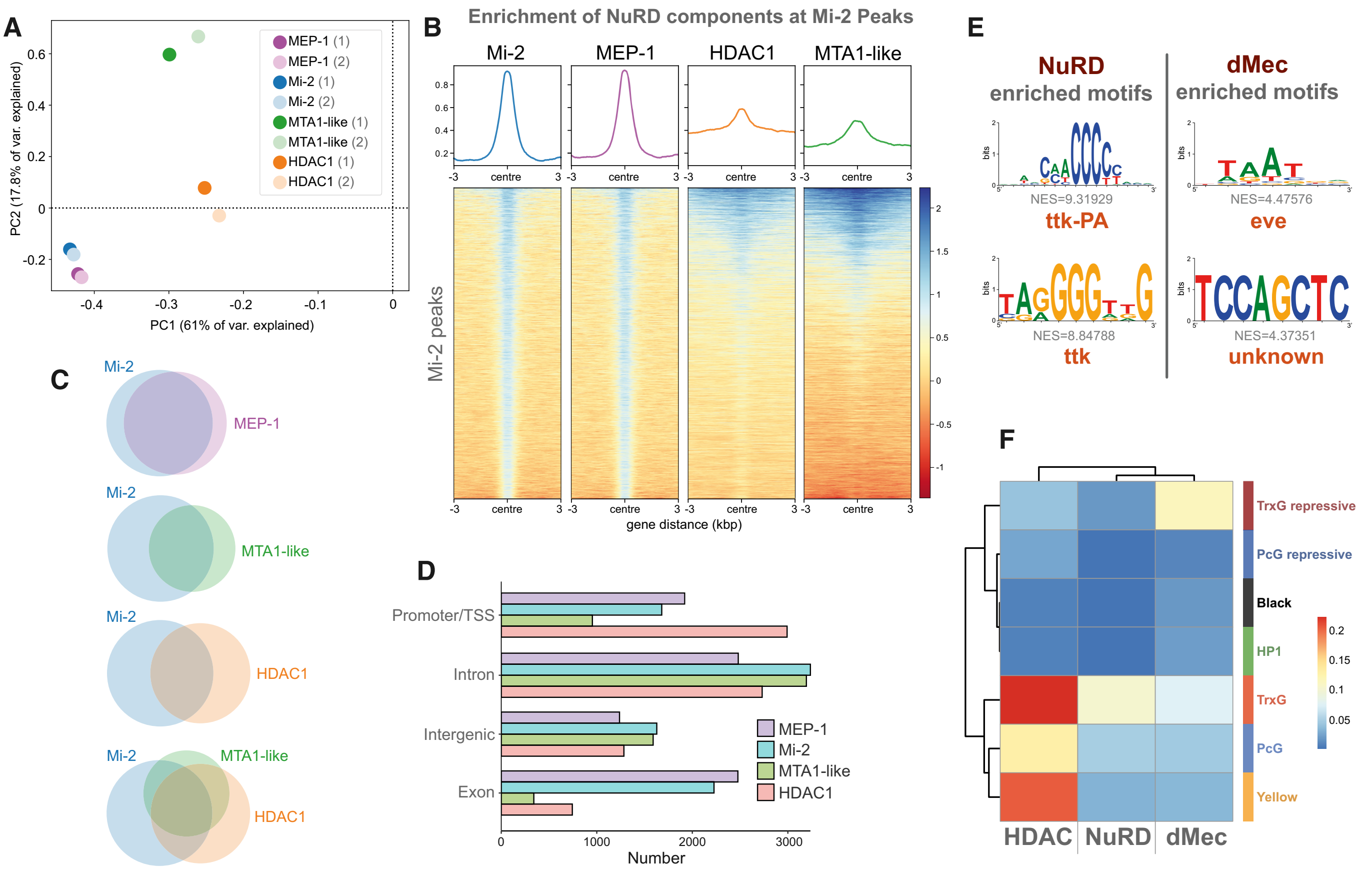

Figure 2. Genomic binding of NuRD components.

A Principal component analysis of all NuRD component binding in neurons including replicates. Mi-2/MEP-1 (blue/purple) cluster independently of MTA1-like (green) and HDAC1 (orange) reflecting greater similarity in their binding profiles.

B Heatmap and enrichment plots indicating binding at Mi-2 peaks.

C Venn diagrams indicating the number of overlapping peaks between NuRD subunits. All intersections between peaks occur at statistically significant frequencies (Fisher's exact test $P < 10^{-24}$).

D Peak annotation for Mi-2, MEP-1, MTA-1like and HDAC1 peaks.

E Top enriched motifs at NuRD and dMec peaks.

F Heatmap indicating jaccard similarity statistic between chromatin states and NuRD peaks (Mi-2, MEP-1, MTA-1like and HDAC1) and dMEC (Mi-2 and MEP-1 only), or HDAC1 peaks.

compared with MTA1-like presumably due to the inclusion of HDAC1 in other chromatin modifying complexes (Fig 2C). We also observed some MTA1-like peaks that did not overlap with Mi-2 or HDAC1, suggesting that MTA1-like may have undefined roles outside of NuRD. As previously indicated, MEP-1 and Mi-2 peaks intersected at almost all loci. Inspection of the genomic features at which the various peaks were found to bind demonstrated further disparity between Mi-2/MEP-1 and MTA1-like/HDAC1. We found that whilst HDAC1 was found to be enriched at promoters and TSS, Mi-2 and MEP-1 were comparatively enriched in gene bodies, particularly in exons (Fig 2D). Together these data suggest that multiple Mi-2-containing complexes interact with chromatin in neurons that may or may not possess histone deacetylase activity. In line with previous reports, we refer to sites bound by Mi-2 and MEP-1 as "dMEC" hereafter (Kunert *et al*, 2009), whilst sites with significant binding of all four profiled components are considered "NuRD" target sites.

dMec and NuRD regulate different genes in neuronal differentiation

Examining the gene ontology (GO) enrichment of genes bound by either NuRD or dMec revealed clear differences in the processes that were regulated by each complex. We found that NuRD-regulated genes were enriched for GO terms related to organismal development, with "nervous system development" amongst the top enriched terms (Fig EV1B). In contrast, genes with dMec peaks were enriched for terms relating to cilia function (Lattao *et al*, 2017) (Fig EV1C) (Appendix Fig S1) reflecting the prevalence of genes that are usually enriched in testis and not expressed in the CNS (although it should be noted that it is possible that some of these genes may also be expressed in fully differentiated sensory neurons). Terms relating to synaptic function were also enriched for genes bound by dMec, which may be expected of a group of genes the expression of which is regulated in neurons. However,

 © 2023 The Authors

inspection of the genes comprising these categories reveals that many of these genes are expressed post-synaptically outside the CNS (*GluRIIA*, *GluRIIC*) or in later-stage pupal CNS in which neurons are in a more differentiated state (e.g. *slo*, *Syt12*). These results are consistent with a model in which dMEC is largely responsible for repression of gene expression, whilst NuRD is involved in coordinating appropriate transcription of neuronal genes.

We examined NuRD and dMec-bound sequences for enrichment of sequence motifs to see if we could identify potential cofactors that may be involved in recruitment or regulation of these complexes. For sequences bound by NuRD, we found that the most highly enriched sequence motifs corresponded well to the consensus sequences for *tramtrack* (*ttk*) (Fig 2E). ttk has previously been shown to interact with MEP-1 to recruit the NuRD complex (Reddy *et al*, 2010), and physical interactions with other NuRD components have also been reported (Rhee *et al*, 2014). In contrast, the *ttk* motif was not enriched for sequences covered by dMec peaks. Instead, the most highly enriched motif identified was a Hox-like motif corresponding most closely to the transcriptional repressor *even-skipped* (*eve*). The differences in the underlying sequences present at NuRD and non-NuRD Mi-2-binding sites suggest that these complexes maybe recruited to target loci by distinct molecular factors.

To gain further insight into how Mi-2-containing complexes regulated gene expression, we compared binding with the state of the underlying chromatin. Chromatin can be grouped into discrete states reflecting the occupancy of key regulatory proteins (Filion *et al*, 2010). We compared the binding of HDAC1, Mi-2, MEP-1 and MTA1-like with published data describing chromatin state domains based on a seven-state model (Marshall & Brand, 2017). We found that HDAC1 binding was associated with broadly permissive chromatin states (Fig 2F). Whilst histone deacetylase activity has been thought to be associated with repression of gene expression, these observations are consistent with previous reports that have described enrichment of HDAC1 binding at active promoters (Wang *et al*, 2009). Indeed, we also observe strong enrichment of HDAC1 binding at transcription start sites (TSS) as previously reported (Fig EV1D). In contrast, Mi-2, MEP-1 and MTA1-like were found to be associated with a mixture of permissive and repressive states, in particular with Trithorax-like permissive and repressive states (Trx-G / Trx-G_repressive). Taking into consideration only regions that are bound by either Mi-2/MEP-1 (dMec), HDAC1 alone, or all four proteins (i.e. NuRD), we saw that whilst NuRD showed the greatest similarity to the Trithorax-like permissive state, Mi2/dMec more strongly coincided with the transcriptionally silent Trithorax-associated repressive state, which is characterised by the presence of Brahma as well as H1 linker histone. These observations support a model in which NuRD-independent Mi-2 binding largely mediates gene silencing/repression, whilst NuRD is involved in the regulation of actively expressed genes.

Mi-2 knockdown results in misexpression of non-CNS genes

To determine the effect of Mi-2 loss on gene expression in neurons of the developing CNS, we performed RNA-seq on larval CNS in which *Mi-2* had been knocked down in neurons by RNAi and compared with controls in which RNAi targeting mCherry was expressed using the same *elav*-GAL4 driver. We found good correlation between replicates (Fig EV2A and B) and observed that the expression of *Mi-2* itself was found to be significantly downregulated (Fig EV2C) in agreement with previous studies (Zacharioudaki *et al*, 2019). We found that knockdown of *Mi-2* resulted in significantly more upregulated genes than downregulated (397 downregulated vs 1,467 upregulated) indicating that in the context of developing neurons, *Mi-2* has a significant role in the repression of gene expression (Fig 3A–C). The top 30 upregulated and top 30 downregulated genes are shown in Fig EV2C and D.

Closer inspection of the top upregulated genes revealed the presence of ectopically expressed genes that are usually expressed in non-CNS tissues such as the germline. For example, *vasa* (*vas*, a well-characterised ovarian germline marker) expression was (~ 3.5 log2 fold-change) increased compared with controls (Fig 3B). Similarly, *gammaTub37C* – a female germline specific isoform of tubulin, was also very highly upregulated (Fig EV2E), as well as *RpS5b*, encoding a germline specific ribosomal protein (Fig EV2F). We also saw evidence of male-germline-specific genes being upregulated, for example, *CG15446* (a transcription factor with testis specific expression) was expressed at much higher levels than controls (Fig 3A). Comparison with tissue-specific transcriptomes indicates that the upregulated genes are usually expressed outside the larval nervous system from a broad range of tissues, including a large cluster of genes that have testis-enriched expression, as well as ovary, fat body and digestive system (Fig 3D and Appendix Fig S2A and B). Furthermore, Gene Ontology (GO) analysis of the significantly upregulated genes revealed enrichment of GO terms that may be associated with neuronal maturation or function, such as “ion transport”, “sensory perception” and “cell projection assembly” (Fig 3E). However, we also saw enrichment for unexpected terms such as “cilium assembly” that are not typically associated with nervous system development or function.

We also observed misexpression of neuronal lineage specific genes that are not usually highly expressed in the larval CNS but are expressed in specific neuronal populations in the adult nervous system. For example, the antennal gene *a10* was seen to be very highly upregulated upon knockdown of *Mi-2* in the larval CNS despite usually having expression restricted to the antennae in adult flies (Figs 3A and EV2C). Therefore, these data indicate that Mi-2 is required to ensure fidelity of neuronal transcriptomes by suppressing expression of spatially and temporally inappropriate genes.

Mi-2/dMEC represses ectopic gene expression independently of HDAC1

Having seen that Mi-2/NuRD components are distributed unequally in the genome, we examined how binding of NuRD components related to differentially upregulated genes. Since previous reports have suggested that dMec has a role in suppressing ectopic gene expression in cultured cells, we questioned whether this complex also plays a greater role in repressing ectopic gene expression in neurons. We found that NuRD binding (i.e. significant binding of Mi-2, MTA1-like and HDAC1) was found at a much lower proportion of upregulated genes compared to those bound by dMec (Mi-2 and MEP-1) (223 and 797 genes, respectively (Fig 3F)). This observation led us to further investigate the role of dMec in regulating gene expression compared with NuRD.

We used qPCR to confirm upregulation of a selection of highly mis-expressed candidate genes in *Mi-2* knockdown flies (Fig 4A).

© 2023 The Authors

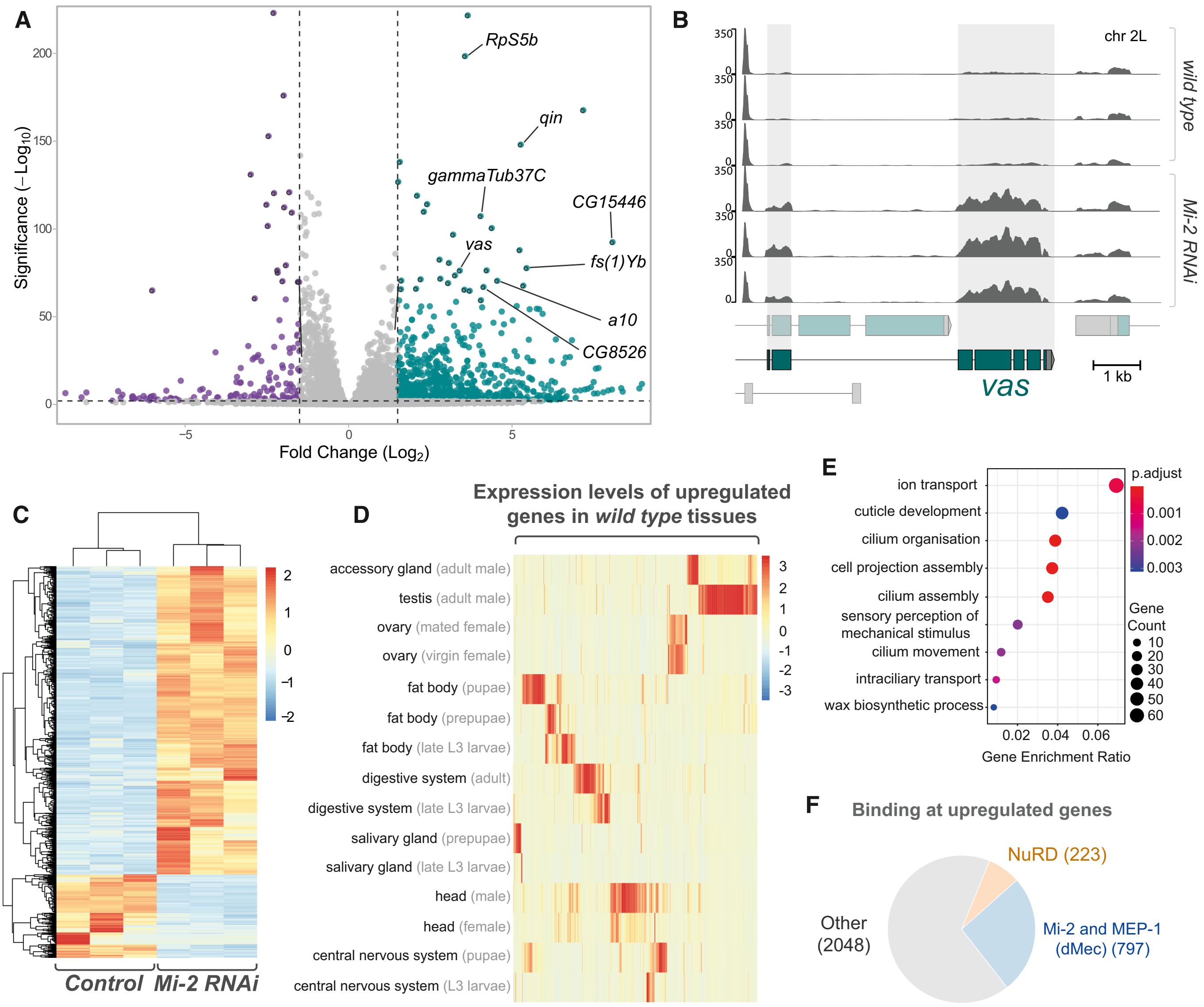

Figure 3. *Mi-2* knockdown in larval neurons results in extensive gene expression changes.

A Volcano plot showing significantly upregulated (green) and downregulated (purple) genes with fold-change. Selected mis-expressed genes are annotated.

B RNA-seq tracks showing *vas* mRNA expression in third instar larval CNS (Y-axis is RPKM).

C Heatmap showing relative changes in normalised read-counts between *Mi-2* RNAi and control replicates for all significantly differentially expressed genes (*P*-adj < 0.05, log2 fold change > 1 or < -1). Significantly more genes are upregulated (1467) than downregulated (397).

D Heatmap showing relative expression levels of upregulated genes across selected *Drosophila* tissues (full version in Appendix Fig S2).

E Enriched gene ontology terms for genes that are upregulated in *Mi-2* RNAi knockdown.

F Proportion of differentially upregulated genes that directly intersect with Mi-2, MEP-1, MTA-1like and HDAC1 peaks, and differentially upregulated genes intersecting with exclusively Mi-2/MEP-1 or all NuRD subunits.

Since RNAi-mediated knockdowns have previously been associated with off-target effects, we repeated these experiments with a second RNAi line which recapitulated the upregulation of ectopic genes previously observed (Appendix Fig S3). Since we saw distinct binding of NuRD subunits, we first asked whether *HDAC1* was required for repression of ectopic genes. We found that depletion of *HDAC1* by RNAi in neurons resulted in lethality during pupal stages similarly to *Mi-2* (Appendix Fig S4). However, *HDAC1* knockdown with two independent RNAi lines had no effect on expression of any of the genes we tested that were upregulated upon *Mi-2* knockdown. Therefore, whilst neuronal *HDAC1* is essential for survival, HDAC1 is not required for repression of some *Mi-2*-induced ectopic gene expression in larval neurons (Fig 4A and Appendix Fig S3).

We questioned whether the expression of ectopic genes in *Mi-2* knockdown was dependent on NuRD (albeit without HDAC1 activity) or mediated by Mi-2 via NuRD-independent mechanisms. To address this, we knocked down *MBD-like*, a core NuRD component thought to bridge the nucleosome remodelling and histone

 © 2023 The Authors

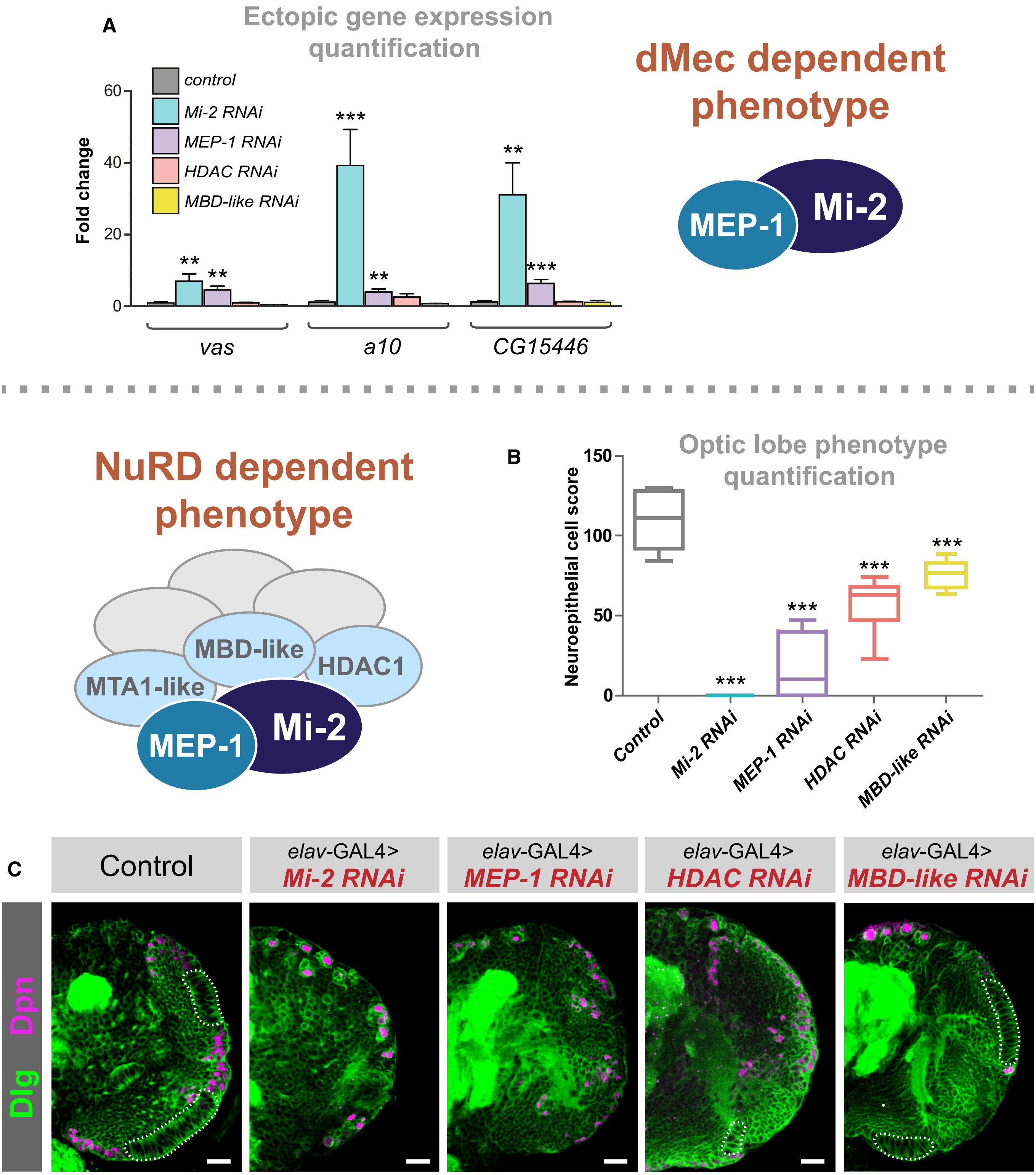

Figure 4. *dMec* knockdown causes ectopic gene expression and *NuRD* knockdown in neurons causes an optic lobe development phenotype.

A Quantification of candidate gene expression (selected from upregulated genes in RNA-seq data) in knockdown genotypes (three biological replicates for each group). $^{**}P < 0.01$ and $^{***}P < 0.001$ (one-tailed student's *t*-test or Welch's *t*-test against control) Represented as mean ± SEM.

B Quantification of optic lobe neuroepithelial cells of genotypes shown in panel C ($n >= 9$ for each group). $^{***}P < 0.001$ (one-tailed student's *t*-test or Welch's *t*-test against control). Represented as a box plot.

C Representative images of control (*elav*-GAL4;; *mCherry*^{RNAi}), *Mi-2* knockdown, *MEP-1* knockdown, *HDAC1* knockdown and *MBD-like* knockdown in the third instar larval CNS (all scale bars = 20 μm). Discs large (Dlg) = green, Deadpan (Dpn) = magenta. Neuroepithelial cells are highlighted by dashed lines.

© 2023 The Authors

deacetylation subunits. In *MBD-like* RNAi knockdown neurons, no difference in gene expression of ectopic target genes was observed (Fig 4A), providing further evidence of *Mi-2* acting independently of NuRD to mediate gene repression. Since MEP-1 binding in the genome appeared to coincide with Mi-2 at almost every locus, we hypothesised that MEP-1 must also be part of the same complex that confers repression of ectopic gene expression. Consistent with this idea, we saw that knockdown of MEP-1 with *elav*-GAL4 was sufficient to cause upregulation of *vas* along with other target genes tested (Fig 4A). Therefore, it appears that dMEC represses the expression of some genes in an MEP-1 and Mi-2-dependent manner, without the requirement for HDAC1-mediated histone deacetylation or other members of the NuRD complex.

NuRD disruption in larval CNS causes severe optic lobe growth defects

Performing GO analysis for downregulated genes from our Mi-2 knockdown RNA-seq indicated enrichment of multiple terms related to cell division and stem cell function (Fig EV3A). This is surprising since we knocked down *Mi-2* in neurons, which are exclusively post-mitotic and do not express genes related to cell-division – therefore, it is unlikely that Mi-2 could directly downregulate the expression of these genes. To further understand the consequences of *Mi-2* depletion in neurons, we examined dissected third instar larval CNS using confocal microscopy. We found that *Mi-2* knockdown resulted in severe optic lobe defects. Mi-2 knockdown brains were smaller, and it was evident that all recognisable optic lobe structures (including inner and outer optic proliferation centres as well as differentiating medulla and lamina neurons) were found to be completely absent (Fig 4B and C). Interestingly, this included proliferative cells of the optic lobes and neuroepithelium (NE), including the neuroblasts. We also examined *Mi-2* knockdown ventral nerve cords (VNCs) and observed no morphological changes or differences in NSC numbers (Fig EV3B and C). Therefore, we attributed the enrichment of GO terms relating to precursor cell function to the fact that a large number of cells expressing these genes were not present in the tissue from which RNA was extracted. Due to this severe phenotype, in which a large fraction of optic lobe cells are absent, we decided that we could not reliably identify genes that were downregulated as a consequence of Mi-2 loss rather than due to absence of cells expressing those genes. However, since we still saw far fewer downregulated than upregulated genes, this reinforces our conclusion that Mi-2 mainly acts to suppress gene expression in this context.

Having seen NuRD-independent effects of *Mi-2* on gene expression, we questioned whether knockdown of other NuRD complex components would phenocopy the optic lobe phenotype we observed when *Mi-2 was* knocked down with *elav*-GAL4. In *MEP-1*, *HDAC1* and *MBD-like* knockdowns, the larval CNS displayed a similar optic lobe defect to that of *Mi-2* (Fig 4B and C). Quantification of NE cells indicated *that MEP-1*, *HDAC1* and *MBD-like* knockdowns had significantly fewer NE cells (Fig 4C); however, the phenotype was less severe with a small number being present in most brains (although this may be due to less efficient RNAi-mediated knockdowns in these lines; Burgold *et al*, 2019). We also observed an optic lobe phenotype with knockdown of *MTA-1* (Fig EV3D and E). Therefore, due to the similarity of phenotypes observed with knockdowns of multiple NuRD complex members, we concluded that whilst *Mi-2* functions to repress some ectopic genes in an NuRD-independent manner, its effect on optic lobe development involves the activity of the NuRD holocomplex (although it remains unclear whether dMEC also contributes to this phenotype). Conversely, the ectopic upregulation of genes is independent of NuRD/histone deacetylation and is not required to produce optic lobe developmental defects in the larva (although since the phenotypes vary in severity, there may be some contribution of dMec-induced ectopic gene expression on NE survival).

Fully differentiated neurons do not require Mi-2 for silencing of ectopic gene expression or proper optic lobe development in larvae

Factors involved in the maintenance of neuronal transcriptomes during early neuronal maturation can have less severe phenotypes in fully differentiated neurons in which synapses have been formed (Southall *et al*, 2014; Hassan *et al*, 2020). This correlates with observed changes to the underlying chromatin landscape which is dynamic during differentiation and neuronal maturation (Marshall & Brand, 2017; Aughey *et al*, 2018). Notch-induced hyperplasia in neuronal lineages is enhanced by the knockdown of *Mi-2*; however, this effect is diminished as cells become more differentiated (Zacharioudaki *et al*, 2019). In our previous knockdown experiments, we employed the *elav*-GAL4 driver to target all neurons in the *Drosophila* larval CNS. This includes a substantial proportion of neurons that are post-mitotic cells, but not fully differentiated to the point at which synaptic proteins are expressed. In fact, it has been suggested that the majority of larval brain *elav*-expressing neurons contribute relatively little to neuronal function (Eichler *et al*, 2017), since they do not possess axons or dendrites or express ion channels associated with neurotransmission (Ravenscroft *et al*, 2020), despite comprising a large proportion of the total cells in the larval brain (preprint: Jiao *et al*, 2021). Therefore, we questioned whether Mi-2-dependent optic lobe phenotypes or de-repression of silenced genes would be observed when Mi-2 was knocked down in either progenitor cells or fully differentiated neurons.

The larval CNS consists of mostly immature neurons that are produced post-embryonically. However, there is a sizeable population of fully differentiated neurons that persist from the embryo that express the mature neuron marker nSyb. The majority of these are found outside of the optic lobes in the central brain and VNC; however, there are a very small number of nSyb cells found in the optic lobe lamina (Jiao *et al*, 2021). To rule out the contribution of signalling from these mature neurons being responsible for the morphological phenotypes, we examined brains in which *Mi-2* RNAi was driven with *nSyb*-GAL4. Knockdown of Mi-2 using *nSyb*-GAL4 did not result in any detectable change in *vas* mRNA levels (Fig 5A). We also tested whether depletion of Mi-2 in adult neurons was sufficient to de-repress *vas* expression using a temperature-sensitive GAL80 to de-repress *Mi-2* RNAi in neurons using *elav*-GAL4 for a 24-h period after eclosing. As in third instar larvae, no change in *vas* expression was detectable in adult heads (Fig EV4A). We also tested for expression of *a10* and *CG15446*, which also did not show any increased expression in adult neurons, indicating that Mi-2 is only required for the repression of ectopic gene expression in the early stages of neuronal development. We used *wor*-GAL4, to drive *Mi-2*

 © 2023 The Authors

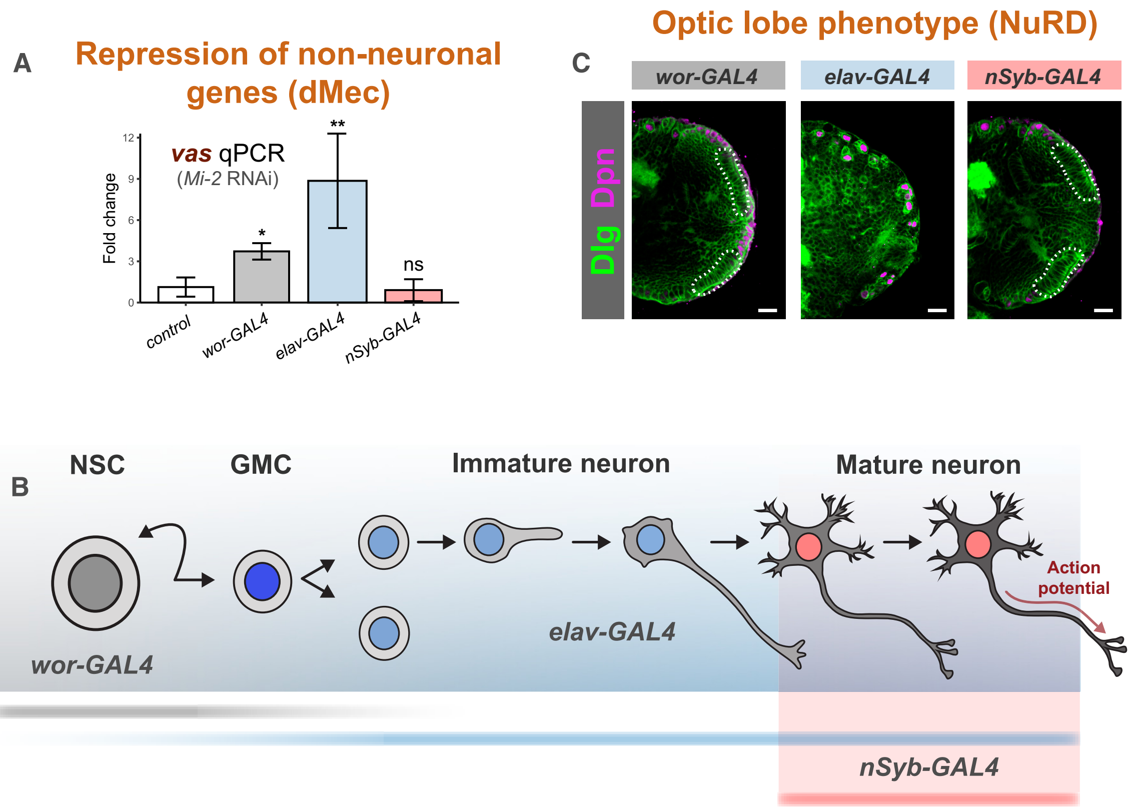

Figure 5. Differential requirements of Mi-2 during neuronal differentiation.

A Ectopic expression of *vas* when *Mi-2* is knocked down at different stages of differentiation (three biological replicates for each group). *$P < 0.05$ and **$P < 0.01$ (one-tailed student's *t*-test against control) Represented as mean ± SEM.

B Representation of expression of GAL4 drivers used.

C Larval brain optic lobe morphology when *Mi-2* is knocked down at different stages of differentiation Elav = green, Dpn = magenta.

RNAi in NSCs (it should be noted that this driver is likely to also affect the newly born GMC and early differentiating neurons due to perdurance; Johnson *et al*, 2018; Fig 5B). Using *wor*-GAL4 to knock down *Mi-2* in NSCs, we performed qPCR to detect changes in gene expression. When *Mi-2* was knocked down in NSCs, the expression of female germline marker *vas* was significantly upregulated (Fig 5A), as previously seen with *elav*-GAL4 knockdowns. *vas* expression was upregulated to a lower extent than when induced with *elav*-GAL4 (~ 4 fold increase compared with ~ 10 fold); however, this may be due to the lower number of affected cells expressing *wor*-GAL4 rather than reflecting a difference in the requirement for Mi-2 for repression of *vas* in NSCs. It should also be noted that it is possible that a small amount of perdurance of GAL4 into NSC progeny may be responsible for de-repression of non-neuronal target genes in immature neurons or GMCs, rather than due to an effect exclusively in NSCs.

Surprisingly, we found that *Mi-2* knockdown in NSCs showed no apparent defects in optic lobe development, with brain lobes having a similar morphology and neuroepithelial cell numbers to controls (Figs 5C and EV4B and C). In contrast to when *Mi-2* was depleted in neurons (Fig 1D), loss of *Mi-2* in NSCs did not result in larval locomotion defects (Fig EV4D). However, *Mi-2* knockdown in NSCs did result in lethality, with 100% dying as pupae. Therefore, since brain size, morphology and movement are normal in these animals, these results suggest that Mi-2 is not required for stem cell division or differentiation into neurons. Nevertheless, Mi-2 function in NSCs is required for the correct functioning of subsequently born neurons.

Having seen that expression of *Mi-2* RNAi in neuronal precursors was not responsible for the optic lobe phenotype, we questioned whether other unexpected expression may be responsible. *elav*-GAL4 may drive some expression in embryonic glial cells (Berger *et al*, 2007); therefore, we questioned whether unexpected glial knockdown of *Mi-2* may have resulted in a disruption of signalling that maintains the optic lobe stem cells. To address this, we repeated the *Mi-2* knockdown with an *elav*-GAL4 driver combined with *repo*-GAL80, to repress unwanted expression of the RNAi hairpin in glia. In these animals, we observed the same severe optic lobe phenotype as previously with *elav*-GAL4 alone (Fig EV4E). These data indicate that *Mi-2* knockdown in neurons may affect optic lobe growth via a non-cell-autonomous mechanism. This may be partly explained by the requirement for innervation by photoreceptor neurons to initiate neurogenesis in the developing visual centres (Selleck & Steller, 1991; Huang & Kunes, 1996), which would be affected by *elav*-GAL4 knockdown of *Mi-2*. We also examined first instar *Mi-2* knockdown brains and found that the typical rosette of NE cells is present (Fig EV4F). Therefore, the NE is present at earlier

© 2023 The Authors

stages of development; however, it is not persisting to later stages. As *elav*-GAL4 has never been reported to be expressed in NEs, the role of any glial expression of *Mi-2* RNAi has been eliminated (Fig EV4E), and knockdown of *Mi-2* in neuroblasts has no phenotype (Fig EV4B and C), this strongly suggests that there is a non-cell-autonomous mechanism occurring. However, a full explanation of this phenotype is beyond the scope of this study, and further work must be conducted to determine the mechanism by which the optic lobe defect occurs.

In contrast to knockdown in all neurons including immature neurons with *elav*-GAL4, depletion of *Mi-2* exclusively in mature neurons did not produce overt morphological phenotypes, and no significant difference in neuroepithelial cell numbers was detected when compared with controls (Figs 5C and EV5A and B). As with the other drivers used, *Mi-2* knockdown with *nSyb*-GAL4 resulted in 100% lethality during pupal stages. Therefore, whilst Mi-2 is required in mature neurons, the morphological defects observed in the larvae do not arise from loss of Mi-2 function in fully differentiated neurons in the larval CNS.

Dynamic Mi-2 binding during neuronal differentiation

We reasoned that varying consequences of *Mi-2* loss at different stages of neuronal differentiation may be reflected by changes to Mi-2 binding in different cell types. To examine differences in Mi-2 genome occupancy in precursor cells vs fully differentiated cells, we profiled Mi-2 (using TaDa) in two further populations of cells in the larval brain. Firstly, in NSCs, *wor*-GAL4 – this driver has been used previously to produce Targeted DamID profiles that are distinguishable from those produced from GMCs/newly born neurons. However, it should be noted that due to perdurance of GAL4, these profiles likely represent the early stages of neurogenesis more generally rather than specifically NSCs. We then also profiled mature neurons (*nSyb*-GAL4) in the larval CNS, in which we saw no change in *vas* expression upon *Mi-2* knockdown. Since these neurons do not reflect the direct descendants of those produced by the *wor*-expressing NSCs in the larval brain (being mostly derived from earlier embryonic neurogenesis), they may also display lineage-specific changes in Mi-2 binding.

Binding profiles between cell types were compared and regions of differential binding identified (Fig 6A and B). Total neurons (*elav*-GAL4) show the largest number of significantly bound peak regions (10,420), with mature neurons binding 7,054 genes (Fig 6B). However, the number of peaks common to each cell type is relatively low. Therefore, Mi-2 binding is highly dynamic during neuronal differentiation. In many individual genes, sites with enriched binding in NSCs showed a different set of enriched Mi-2-binding sites in immature neurons (Fig 6A and C). A similar change occurs from immature to mature neurons, with 2,144 genes that contain both immature-vs-mature and mature-vs-immature regions of Mi-2 enrichment (Fig 6C and the example of *chn* in Fig 6A).

Motif enrichment analysis of the differentially bound regions identified transcription factor binding sites (Fig 6D). Binding sites for l(3)neo38, crol and ttk88 are present in the NSC-vs-immature comparison, suggesting that these sites are occluded in NSCs but available for binding in neural progeny. Interestingly, l(3)neo38 occupies a set of putative neuronal enhancers in the developing embryo and is required for the proper formation of the larval brain (Reddington *et al*, 2020). l(3)neo38 and crol have both been implicated in the regulation of heterochromatin silencing (Schneiderman, 2010; Swenson, 2016). While ttk88 sites are enriched for binding in NSC-vs-immature neurons, ttk69 sites (a different splice isoform) are enriched in the immature-vs-mature comparison. Finally, fd102C and Cf2 sites have greater Mi-2 binding in mature-vs-immature neurons. Fd102C is an uncharacterised forkhead transcription factor highly expressed in the developing and adult nervous system (Krause *et al*, 2022), and Cf2 is a zinc-finger factor involved in follicle cell fate and myogenic gene expression (Hsu *et al*, 1996; Bagni *et al*, 2002). These data indicate that a dynamic repertoire of transcription factors may direct or be influenced by dynamic Mi-2/NuRD binding during neuronal maturation. It is clear from the broad changes in binding that Mi-2 coordinates a distinct gene expression programme in early neuronal lineages compared with fully differentiated neurons corresponding to the differences in phenotypic outcomes observed (although some of the observed changes may be due to embryonic vs larval neuronal lineage differences).

Su(H) (Notch co-factor) motifs were enriched in both immature-vs-NSC and immature-vs-mature regions (Fig 6D). This aligns with previous work showing that Mi-2 is required to deactivate notch-responsive stem cell enhancers in neuronal progeny (Zacharioudaki *et al*, 2019). Mi-2 was implicated in the silencing of genes in the *E(spl)-C* locus, with broad binding of Mi-2 across this locus having been demonstrated in *Drosophila* cell lines (Ho *et al*, 2014; Kreher *et al*, 2017). Consistent with this result we saw strong binding of Mi-2 across the *E(spl)-C* locus in both NSCs and immature neurons (Fig EV5C). This study also showed that in more mature neurons, Mi-2 depletion was not sufficient to enhance notch-induced overgrowth. We saw no significant binding of Mi-2 in mature neurons across the entire region (approx. 50 kb), supporting the conjecture that in more fully differentiated cells, continued chromatin remodelling of the *E(spl)* genes by Mi-2 is not required for their repression.

Discussion

Our data provide evidence for Mi-2 activity in repressing ectopic gene expression independently of NuRD. Mi-2 from *Drosophila* or humans is sufficient for the remodelling of nucleosomes *in vitro*, in the absence of other NuRD components (Brehm *et al*, 2000; Wang & Zhang, 2001). In mouse embryos, Mi-2 orthologue CHD4 was shown to be necessary for lineage specification in a manner that was independent of MBD proteins that are required for a functional NuRD complex (O'Shaughnessy-Kirwan *et al*, 2015). Similarly, loss of CHD4 in human erythroid cells resulted in de-repression of fetal *γ-globin*, which occurred independently of NuRD (Amaya *et al*, 2013). Furthermore, similarly to what we observe in neurons, MBD3 was found to bind at less than half of all CHD4 sites in mouse ESCs and were anti-correlated to histone markers associated with transcription (Bornelov *et al*, 2018). Therefore, it is likely that NuRD-independent Mi-2 regulation of gene expression is a conserved feature in animal cells.

A previous study concluded that Mi-2 in complex with MEP-1 (dMec) was the dominant Mi-2 containing complex in *Drosophila* Kc cells (Kunert *et al*, 2009). From this observation it could be inferred that NuRD plays a relatively minor role in the regulation of

© 2023 The Authors

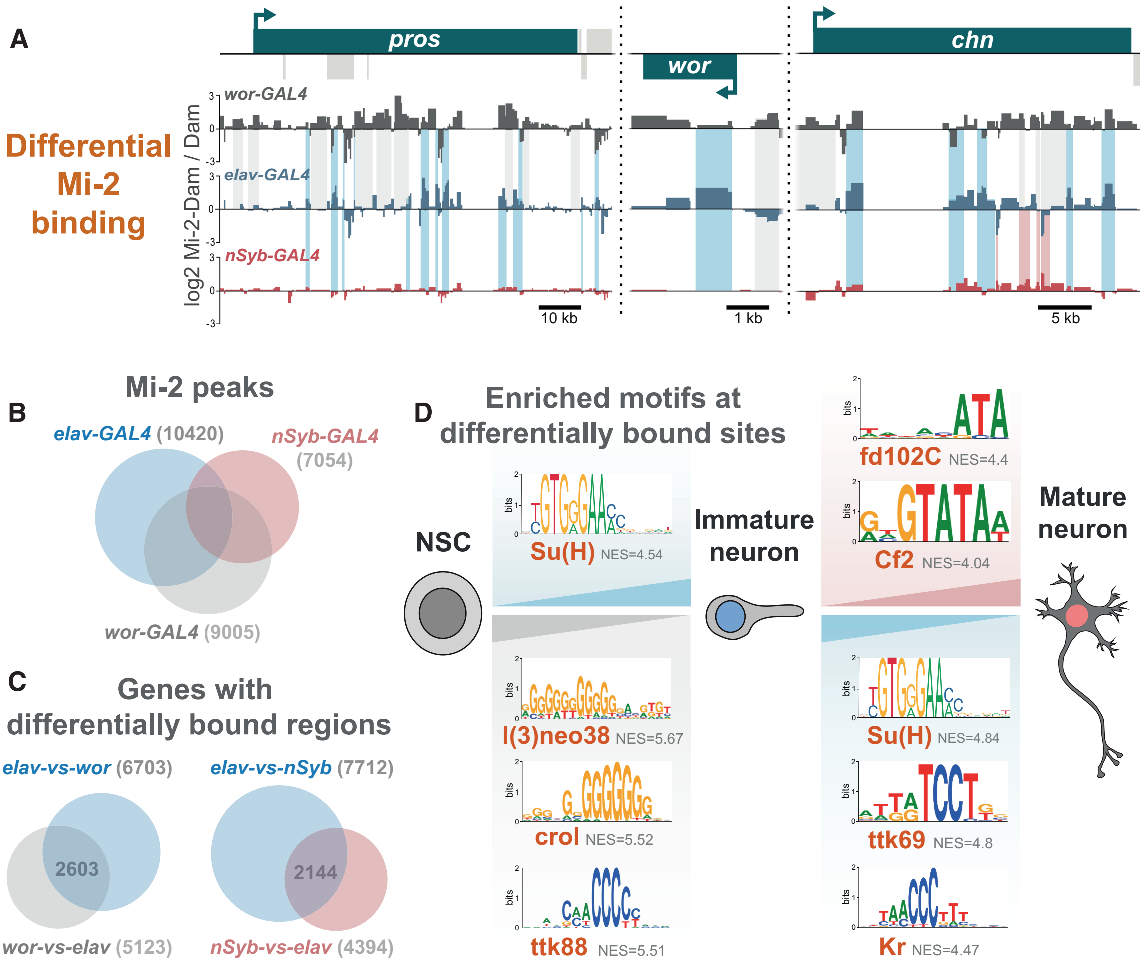

Figure 6. Dynamic Mi-2 binding during neuronal differentiation.

A Examples of differential genomic binding of Mi-2 at neural genes.
B Overlap of Mi-2 binding peaks in NSCs, immature neurons and mature neurons.
C Number and overlap of genes with differential Mi-2 binding.
D Motif analysis of differentially bound regions.

gene expression. However, this conclusion was drawn based on biochemical interactions and relative stoichiometries of NuRD components. We find that in contrast to the abundance of dMec, around a third of Mi-2 peaks intersect with MTA1-like, indicating that NuRD is prevalent at a large number of loci. Furthermore, severe optic lobe phenotypes were observed with multiple NuRD components, which did not always include de-repression of lineage-inappropriate genes. These data indicate that despite being present at lower amounts than dMec, NuRD plays a critical role in nervous system development. Whilst it is possible that the proportions of NuRD/dMec may differ between the CNS and cultured cells, our data suggest that the relative amounts of protein detected by biochemical means may not be reflective of either the distribution of sites at which complexes are associated in the genome or the severity of consequences resulting from their loss. It is currently unclear whether an analogous complex to dMEC exists in mammals. MEP-1 does not share direct orthology with any single mammalian transcription factors; however, since it is clear that CHD4 is capable of conferring NuRD-independent activities in mammalian cells, it is possible that there are unidentified mammalian genes that act in an analogous role to MEP-1.

Our data indicate that Mi-2 represses non-neuronal genes such as those normally expressed in the germline and that this is largely independent of NuRD. Interestingly, Mi-2/dMec depletion resulted in proneural gene expression in Kc cells (Kunert *et al*, 2009), suggesting that Mi-2 may repress ectopic gene expression in multiple tissue types dependent on context. A similar phenotype has also been reported in *C. elegans* in which MEP-1/Mi-2 knockdown resulted in expression of germline genes in somatic tissues, indicating that this may be a conserved function of Mi-2 (Unhavaithaya *et al*, 2002). It is possible that in mammals, NuRD-independent CHD4 function is also required for gene repression since loss of CHD4 in lymphocytes results in upregulation of non-haematopoietic genes (Arends *et al*, 2019). However, it remains unclear whether this transcriptional repression is dependent on histone deacetylase activity/NuRD or NuRD-independent CHD4 activity.

Mi-2 is only required to suppress ectopic gene expression up to the early stages of neuronal development, whereas in more fully differentiated cells, it is dispensable. Therefore, it is likely that other molecular factors are required for the repression of these genes following synaptogenesis. Mi-2/dMEC may be required to establish a stable epigenetic state following recruitment by sequence specific transcription factor binding, after which these states are maintained by separate chromatin modifying proteins or complexes. The degree of differential binding of Mi-2 observed during neuronal differentiation is consistent with the idea that Mi-2 coordinates a distinct gene expression programme during early neuronal differentiation compared with mature neurons. The identification of different transcription factor binding sites during development suggests that either the cognate transcription factor is recruiting Mi-2 to these sites or that Mi-2 is independently positioned there to establish a chromatin state to prevent the binding of the factor. Since it seems that Mi-2 exhibits at least two independent modes of interacting with chromatin (i.e. via dMEC/NuRD), either or both of these models may be true depending on the presence of other subunits and wider genomic context. Su(H)-binding sites are found at sites enriched in immature neurons versus both NSCs and mature neurons (Fig 6D). Furthermore, l(3)neo38-binding sites, which have been identified at neural enhancers (Reddington *et al*, 2020), are more strongly bound in NSCs compared with immature neurons. Therefore, for at least Su(H) and l(3)neo38, Mi-2 appears to be blocking binding of these factors (loci specific) at stages of differentiation where their function is not required/wanted.

Together, our data highlight that the activity of Mi-2 is dependent on both its molecular and developmental context. These data highlight the importance of detailed analyses of individual subunits in inferring functions of chromatin modifying complexes in complex multicellular tissues to fully understand their role in gene regulation.

Materials and Methods

Fly stocks

Unless stated otherwise, flies were kept on standard fly food media at 25 degrees. A full list of fly stocks used in this study can be found in Appendix Table S1. Mixed sex larvae/flies were used for all experiments.

Immunohistochemistry and imaging

Third instar larval CNS was dissected in PBS and fixed for 20 min with 4% formaldehyde in PBS. The following antibodies were used in this study: rat anti-Elav (7E8A10 – Developmental Studies Hybridoma Bank), chicken anti-GFP (600-901-215 – Thermo scientific), mouse anti-DLG (4F3 – Developmental Studies Hybridoma Bank) and guinea pig anti-Dpn (kind gift from A. Brand). Samples were imaged using a Zeiss LSM510 confocal microscope and images processed using LSM Image Browser and image J. To quantify an OPC NE cell score for each genotype, third instar larval brains were mounted dorsal side down, and full image stacks (2 μm intervals) of the brain lobe were taken using the DLG channel. The number of OPC NE cells were counted at image slices at ¼, ½ and ¾ through the stack (identified by position and morphology) and added together to give the score. To quantify the number of Dpn-positive cells in VNCs, whole VNC image stacks were analysed using the 3D Objects Counter in Fiji (Bolte & Cordelieres, 2006). For each genotype, a Shapiro–Wilks test was performed to calculate if the experimental and control data had normal distributions. If they did, a one-tailed student's *t*-test was applied, and if not, a Welch's *t*-test was applied.

Generation of targeted DamID lines

MEP-1 and *Mi-2* were amplified by PCR from cDNA with primers including 30 bp complementary sequence to *pUASTattB-LT3-Dam* (Southall *et al*, 2013; see Appendix Table S2). Linearised *pUAST-LT3-Dam* (digested with NotI and XbaI) was combined with either *MEP-1* or *Mi-2* PCR-amplified coding sequences in a Gibson assembly reaction using NEBuilder® HiFi DNA Assembly kit (NEB) following manufacturers protocols. Resulting N-terminal fusion constructs were injected into *attP2* flies (*y w P{y[+t7.7] = nos-phiC31\int.NLS}X #12;; P{y[+t7.7] = CaryP}attP2* – Department of Genetics Microinjection Service, Cambridge) for integration on the third chromosome.

UAS-MTA-1-like-Dam and *UAS-HDAC1-Dam* were generated using the FlyORF-TaDa cassette exchange protocol as previously described (Aughey *et al*, 2021). Briefly, *UAS-HDAC1* and *UAS-MTA1-like* FlyORF lines were crossed to *hsFLP; TaDa-FlyORF*. Progeny were heat-shocked at 37 degrees for 45 min at 96 h following larval hatching. Resulting males were crossed to *Dr/TM6B* and white-eyed F2 males and female virgins used to create a stable stock.

Targeted DamID

Dam fusion or Dam-only males were crossed to virgins of the appropriate driver line for expression of Dam in the desired cell-types. For expression in NBs, all larval neurons (including immature neurons), or mature neurons only – the drivers *wor*-GAL4*; tub*-GAL80^{ts}, *elav*-GAL4*; tub*-GAL80^{ts}, *and nSyb*-GAL4*; tub*-GAL80^{ts}, were used, respectively. Embryos were collected for 4 h and then raised at 18°C to prevent premature Dam expression. Animals were transferred to 29°C at 8 days after embryo deposition for 24 h. Sixty larval central nervous systems were dissected and stored at −80 degrees. Genomic DNA extraction, methylated DNA enrichment and library preparation were carried out as previously described (Marshall *et al*, 2016). Two biological replicates were generated for each experiment.

Libraries were sequenced with 50 bp single-end sequencing on Illumina HiSeq or NovaSeq platforms. More than 15 million reads were obtained for each library. Data were processed using a previously described Perl pipeline (Marshall & Brand, 2015; https://github.com/owenjm/damidseq_pipeline).

Peak calling was performed using a previously described Perl program (available at https://github.com/tonysouthall/Peak_calling_DamID) which allows for the identification of broadly bound regions that characterise DamID data (Estacio-Gomez *et al*, 2020). Briefly, false discovery rate (FDR) was calculated for regions consisting of > 1 GATC bounded fragments for each replicate. Significant (FDR < 0.01%) regions present in all replicates are merged to form a final peak file. Enriched motifs discovery was performed using i-cisTarget on significant peak regions (Herrmann

© 2023 The Authors

et al, 2012). Intersecting regions for comparison of binding between Mi-2, MEP-1, MTA1-like or HDAC1 were computed using bedtools intersect utility (Quinlan & Hall, 2010). Similarity between binding peaks was determined using the bedtools jaccard utility, which calculates the ratio between the number of intersecting base pairs and the union between two coordinate sets (Favorov *et al*, 2012).

Larval crawling assays

Larval crawling assays were conducted as in Nichols *et al* (2012). Briefly, individual third instar wandering larva were placed on room temperature agar with a 10 mm^2 grid placed underneath as a distance reference. Larvae were left to acclimatise for 1 min after which larval movement was tracked visually. Ten animals were used per genotype with three technical replicates performed per animal.

Developmental survival assays

Flies of the appropriate genotype were left in cages containing standard food media supplemented with yeast and left to acclimatise over three nights at 25°C. Food media was exchanged for apple juice agar, and flies left to lay for 5 h. Following the laying period, 100 embryos were transferred to fresh food vials to allow for counting of surviving adults and pupae.

RNA extraction

Three biological replicates were used for knockdown or control samples. Third instar larval CNS was dissected from 30 animals per replicate and total RNA extracted using TRIzol reagent (https://tools.thermofisher.com/content/sfs/manuals/trizol_reagent.pdf) following suppliers protocols. Library preparation and sequencing were subsequently performed by Beijing Genomics Institute (BGI). Sequencing data were mapped to the *Drosophila* genome (release 6.22) using STAR (Dobin *et al*, 2013). Mapped files were collected in a matrix using featureCounts from the Rsubread package (Liao *et al*, 2019). Differential expression analysis and MA plots were carried out using the Deseq2 R package (Love *et al*, 2014). Genes that had an adjusted *P*-value < 0.05 and a log2 fold change > 1 (for upregulated) or less than -1 (for downregulated) were classified as significant. Heatmap generated using pheatmap package in R. Analysis of tissue specific gene enrichment was performed using DGET (Hu *et al*, 2017).

qRT-PCR

Following RNA extraction, reverse transcription was performed using the iScript cDNA synthesis kit (Bio-Rad), following manufacturer's protocols. qRT-PCR was carried out using iTaq Universal SYBR Green Supermix (Bio-Rad). Expression of target genes was normalised to *Ribosomal Protein L4* (*Rpl4*) and compared with expression in control samples expressing RNAi targeting *mCherry*. Primers amplifying sequences < 200 bp were designed to span exon junctions where possible (sequences used can be found in Appendix Table S2). Three biological replicates were carried out for each genotype, with at least two technical replicates for each biological replicate. For each genotype, a Shapiro–Wilks test was performed to calculate if the experimental and control data had normal distributions. If they did, a one-tailed student's *t*-test was applied, and if not, a Welch's *t*-test was applied.

Data availability

All raw sequence files and processed files have been deposited in the National Center for Biotechnology Information Gene Expression Omnibus (GSE199146; http://www.ncbi.nlm.nih.gov/geo/query/acc.cgi?acc=GSE199146).

Expanded View for this article is available online.

Acknowledgements
We would like to thank the Southall lab and Imperial College fly community for help and feedback on this project. We would like to thank Andrea Brand for generously providing fly stocks and the Dpn antibody. We also thank the Bloomington *Drosophila* Stock Center (NIH P40OD018537) for fly stocks. This work was funded by a Wellcome Trust Investigator grant 104567/Z/14/Z to TDS and a BBSRC grant BB/P017924/1 to TDS and GNA.

Author contributions
Gabriel N Aughey: Conceptualization; data curation; formal analysis; supervision; investigation; visualization; methodology; writing – original draft; writing – review and editing. **Elhana Forsberg:** Formal analysis; investigation. **Krista Grimes:** Formal analysis; investigation. **Shen Zhang:** Formal analysis; investigation. **Tony D Southall:** Conceptualization; data curation; formal analysis; supervision; funding acquisition; investigation; visualization; methodology; project administration; writing – review and editing.

Disclosure and competing interests statement
The authors declare that they have no conflict of interest.

References

Amaya M, Desai M, Gnanapragasam MN, Wang SZ, Zu Zhu S, Williams DC Jr, Ginder GD (2013) Mi2beta-mediated silencing of the fetal gamma-globin gene in adult erythroid cells. *Blood* 121: 3493–3501

Arends T, Dege C, Bortnick A, Danhorn T, Knapp JR, Jia H, Harmacek L, Fleenor CJ, Straign D, Walton K *et al* (2019) CHD4 is essential for transcriptional repression and lineage progression in B lymphopoiesis. *Proc Natl Acad Sci U S A* 116: 10927–10936

Aughey GN, Estacio Gomez A, Thomson J, Yin H, Southall TD (2018) CATaDa reveals global remodelling of chromatin accessibility during stem cell differentiation *in vivo*. *Elife* 7: e32341

Aughey GN, Cheetham SW, Southall TD (2019) DamID as a versatile tool for understanding gene regulation. *Development* 146: dev173666

Aughey GN, Delandre C, McMullen JPD, Southall TD, Marshall OJ (2021) FlyORF-TaDa allows rapid generation of new lines forin vivocell-type-specific profiling of protein–DNA interactions in *Drosophila melanogaster*. *G3 (Bethesda)* 11: 11

Bagni C, Bray S, Gogos JA, Kafatos FC, Hsu T (2002) The *Drosophila* zinc finger transcription factor CF2 is a myogenic marker downstream of MEF2 during muscle development. *Mech Dev* 117: 265–268

Berger C, Renner S, Luer K, Technau GM (2007) The commonly used marker ELAV is transiently expressed in neuroblasts and glial cells in the *Drosophila* embryonic CNS. *Dev Dyn* 236: 3562–3568

Bolte S, Cordelieres FP (2006) A guided tour into subcellular colocalization analysis in light microscopy. *J Microsc* 224: 213 – 232

Bornelov S, Reynolds N, Xenophontos M, Gharbi S, Johnstone E, Floyd R, Ralser M, Signolet J, Loos R, Dietmann S *et al* (2018) The nucleosome remodeling and Deacetylation complex modulates chromatin structure at sites of active transcription to fine-tune gene expression. *Mol Cell* 71: 56 – 72

Brehm A, Langst G, Kehle J, Clapier CR, Imhof A, Eberharter A, Muller J, Becker PB (2000) dMi-2 and ISWI chromatin remodelling factors have distinct nucleosome binding and mobilization properties. *EMBO J* 19: 4332 – 4341

Burgold T, Barber M, Kloet S, Cramard J, Gharbi S, Floyd R, Kinoshita M, Ralser M, Vermeulen M, Reynolds N *et al* (2019) The nucleosome remodelling and deacetylation complex suppresses transcriptional noise during lineage commitment. *EMBO J* 38: e100788

Dobin A, Davis CA, Schlesinger F, Drenkow J, Zaleski C, Jha S, Batut P, Chaisson M, Gingeras TR (2013) STAR: ultrafast universal RNA-seq aligner. *Bioinformatics* 29: 15 – 21

Eichler K, Li F, Litwin-Kumar A, Park Y, Andrade I, Schneider-Mizell CM, Saumweber T, Huser A, Eschbach C, Gerber B *et al* (2017) The complete connectome of a learning and memory centre in an insect brain. *Nature* 548: 175 – 182

Estacio-Gomez A, Hassan A, Walmsley E, Le LW, Southall TD (2020) Dynamic neurotransmitter specific transcription factor expression profiles during *Drosophila* development. *Biol Open* 9: bio052928

Favorov A, Mularoni L, Cope LM, Medvedeva Y, Mironov AA, Makeev VJ, Wheelan SJ (2012) Exploring massive, genome scale datasets with the GenometriCorr package. *PLoS Comput Biol* 8: e1002529

Filion GJ, van Bemmel JG, Braunschweig U, Talhout W, Kind J, Ward LD, Brugman W, de Castro IJ, Kerkhoven RM, Bussemaker HJ *et al* (2010) Systematic protein location mapping reveals five principal chromatin types in *Drosophila* cells. *Cell* 143: 212 – 224

Gomez-Del Arco P, Perdiguero E, Yunes-Leites PS, Acin-Perez R, Zeini M, Garcia-Gomez A, Sreenivasan K, Jimenez-Alcazar M, Segales J, Lopez-Maderuelo D *et al* (2016) The chromatin remodeling complex Chd4/NuRD controls striated muscle identity and metabolic homeostasis. *Cell Metab* 23: 881 – 892

Hassan A, Araguas Rodriguez P, Heidmann SK, Walmsley EL, Aughey GN, Southall TD (2020) Condensin I subunit cap-G is essential for proper gene expression during the maturation of post-mitotic neurons. *Elife* 9: e55159

Herrmann C, van de Sande B, Potier D, Aerts S (2012) i-cisTarget: an integrative genomics method for the prediction of regulatory features and cis-regulatory modules. *Nucleic Acids Res* 40: e114

Ho JW, Jung YL, Liu T, Alver BH, Lee S, Ikegami K, Sohn KA, Minoda A, Tolstorukov MY, Appert A *et al* (2014) Comparative analysis of metazoan chromatin organization. *Nature* 512: 449 – 452

Hoffmann A, Spengler D (2019) Chromatin remodeling complex NuRD in neurodevelopment and neurodevelopmental disorders. *Front Genet* 10: 682

Hsu T, Bagni C, Sutherland JD, Kafatos FC (1996) The transcriptional factor CF2 is a mediator of EGF-R-activated dorsoventral patterning in *Drosophila* oogenesis. *Genes Dev* 10: 1411 – 1421

Hu Y, Comjean A, Perrimon N, Mohr SE (2017) The *Drosophila* gene expression tool (DGET) for expression analyses. *BMC Bioinformatics* 18: 98

Huang Z, Kunes S (1996) Hedgehog, transmitted along retinal axons, triggers neurogenesis in the developing visual centers of the *Drosophila* brain. *Cell* 86: 411 – 422

Janssens J, Aibar S, Taskiran II, Ismail JN, Gomez AE, Aughey G, Spanier KI, De Rop FV, Gonzalez-Blas CB, Dionne M *et al* (2022) Decoding gene regulation in the fly brain. *Nature* 601: 630 – 636

Jiao W, Spreemann G, Ruchti E, Banerjee S, Shi Y, Stowers RS, Hess K, McCabe BD (2021) Intact *Drosophila* whole brain cellular quantitation reveals sexual dimorphism. *bioRxiv* https://doi.org/10.1101/2021.11.03.467146 [PREPRINT]

Johnson PW, Doe CQ, Lai SL (2018) *Drosophila* nucleostemin 3 is required to maintain larval neuroblast proliferation. *Dev Biol* 440: 1 – 12

Krause SA, Overend G, Dow JAT, Leader DP (2022) FlyAtlas 2 in 2022: enhancements to the *Drosophila melanogaster* expression atlas. *Nucleic Acids Res* 50: D1010 – D1015

Kreher J, Kovac K, Bouazoune K, Macinkovic I, Ernst AL, Engelen E, Pahl R, Finkernagel F, Murawska M, Ullah I *et al* (2017) EcR recruits dMi-2 and increases efficiency of dMi-2-mediated remodelling to constrain transcription of hormone-regulated genes. *Nat Commun* 8: 14806

Kunert N, Wagner E, Murawska M, Klinker H, Kremmer E, Brehm A (2009) dMec: a novel Mi-2 chromatin remodelling complex involved in transcriptional repression. *EMBO J* 28: 533 – 544

Lattao R, Kovacs L, Glover DM (2017) The centrioles, centrosomes, basal bodies, and cilia of *Drosophila melanogaster*. *Genetics* 206: 33 – 53

Liao Y, Smyth GK, Shi W (2019) The R package Rsubread is easier, faster, cheaper and better for alignment and quantification of RNA sequencing reads. *Nucleic Acids Res* 47: e47

Love MI, Huber W, Anders S (2014) Moderated estimation of fold change and dispersion for RNA-seq data with DESeq2. *Genome Biol* 15: 550

Low JK, Webb SR, Silva AP, Saathoff H, Ryan DP, Torrado M, Brofelth M, Parker BL, Shepherd NE, Mackay JP (2016) CHD4 is a peripheral component of the nucleosome remodeling and deacetylase complex. *J Biol Chem* 291: 15853 – 15866

Marshall OJ, Brand AH (2015) damidseq_pipeline: an automated pipeline for processing DamID sequencing datasets. *Bioinformatics* 31: 3371 – 3373

Marshall OJ, Brand AH (2017) Chromatin state changes during neural development revealed by in vivo cell-type specific profiling. *Nat Commun* 8: 2271

Marshall OJ, Southall TD, Cheetham SW, Brand AH (2016) Cell-type-specific profiling of protein-DNA interactions without cell isolation using targeted DamID with next-generation sequencing. *Nat Protoc* 11: 1586 – 1598

Nichols CD, Becnel J, Pandey UB (2012) Methods to assay *Drosophila* behavior. *J Vis Exp* 61: 3795

Nitarska J, Smith JG, Sherlock WT, Hillege MM, Nott A, Barshop WD, Vashisht AA, Wohlschlegel JA, Mitter R, Riccio A (2016) A functional switch of NuRD chromatin remodeling complex subunits regulates mouse cortical development. *Cell Rep* 17: 1683 – 1698

O'Shaughnessy-Kirwan A, Signolet J, Costello I, Gharbi S, Hendrich B (2015) Constraint of gene expression by the chromatin remodelling protein CHD4 facilitates lineage specification. *Development* 142: 2586 – 2597

Preissl S, Fang R, Huang H, Zhao Y, Raviram R, Gorkin DU, Zhang Y, Sos BC, Afzal V, Dickel DE *et al* (2018) Single-nucleus analysis of accessible chromatin in developing mouse forebrain reveals cell-type-specific transcriptional regulation. *Nat Neurosci* 21: 432 – 439

Quinlan AR, Hall IM (2010) BEDTools: a flexible suite of utilities for comparing genomic features. *Bioinformatics* 26: 841 – 842

Ragheb R, Gharbi S, Cramard J, Ogundele O, Kloet SL, Burgold T, Vermeulen M, Reynolds N, Hendrich B (2020) Differential regulation of lineage commitment in human and mouse primed pluripotent stem cells by the nucleosome remodelling and deacetylation complex. *Stem Cell Res* 46: 101867

Rais Y, Zviran A, Geula S, Gafni O, Chomsky E, Viukov S, Mansour AA, Caspi I, Krupalnik V, Zerbib M *et al* (2013) Deterministic direct reprogramming of somatic cells to pluripotency. *Nature* 502: 65 – 70

© 2023 The Authors

Ravenscroft TA, Janssens J, Lee PT, Tepe B, Marcogliese PC, Makhzami S, Holmes TC, Aerts S, Bellen HJ (2020) *Drosophila* voltage-gated sodium channels are only expressed in active neurons and are localized to distal axonal initial segment-like domains. *J Neurosci* 40: 7999 – 8024

Reddington JP, Garfield DA, Sigalova OM, Karabacak Calviello A, Marco-Ferreres R, Girardot C, Viales RR, Degner JF, Ohler U, Furlong EEM (2020) Lineage-resolved enhancer and promoter usage during a time course of embryogenesis. *Dev Cell* 55: 648 – 664

Reddy BA, Bajpe PK, Bassett A, Moshkin YM, Kozhevnikova E, Bezstarosti K, Demmers JA, Travers AA, Verrijzer CP (2010) *Drosophila* transcription factor Tramtrack69 binds MEP1 to recruit the chromatin remodeler NuRD. *Mol Cell Biol* 30: 5234 – 5244

Rhee DY, Cho DY, Zhai B, Slattery M, Ma L, Mintseris J, Wong CY, White KP, Celniker SE, Przytycka TM *et al* (2014) Transcription factor networks in *Drosophila melanogaster*. *Cell Rep* 8: 2031 – 2043

Schneiderman JI, Goldstein S, Ahmad K (2010) Perturbation analysis of heterochromatin-mediated gene silencing and somatic inheritance. *PLoS Genet* 6: e1001095

Selleck SB, Steller H (1991) The influence of retinal innervation on neurogenesis in the first optic ganglion of *Drosophila*. *Neuron* 6: 83 – 99

Southall TD, Gold KS, Egger B, Davidson CM, Caygill EE, Marshall OJ, Brand AH (2013) Cell-type-specific profiling of gene expression and chromatin binding without cell isolation: assaying RNA pol II occupancy in neural stem cells. *Dev Cell* 26: 101 – 112

Southall TD, Davidson CM, Miller C, Carr A, Brand AH (2014) Dedifferentiation of neurons precedes tumor formation in Lola mutants. *Dev Cell* 28: 685 – 696

Swenson JM, Colmenares SU, Strom AR, Costes SV, Karpen GH (2016) The composition and organization of *Drosophila* heterochromatin are heterogeneous and dynamic. *eLife* 5: e16096

Tie F, Furuyama T, Prasad-Sinha J, Jane E, Harte PJ (2001) The *Drosophila* Polycomb group proteins ESC and E(Z) are present in a complex containing the histone-binding protein p55 and the histone deacetylase RPD3. *Development* 128: 275 – 286

Tong JK, Hassig CA, Schnitzler GR, Kingston RE, Schreiber SL (1998) Chromatin deacetylation by an ATP-dependent nucleosome remodelling complex. *Nature* 395: 917 – 921

Unhavaithaya Y, Shin TH, Miliaras N, Lee J, Oyama T, Mello CC (2002) MEP-1 and a homolog of the NURD complex component Mi-2 act together to maintain germline-soma distinctions in *C. elegans*. *Cell* 111: 991 – 1002

Wade PA, Jones PL, Vermaak D, Wolffe AP (1998) A multiple subunit Mi-2 histone deacetylase from *Xenopus laevis* cofractionates with an associated Snf2 superfamily ATPase. *Curr Biol* 8: 843 – 846

Wang HB, Zhang Y (2001) Mi2, an auto-antigen for dermatomyositis, is an ATP-dependent nucleosome remodeling factor. *Nucleic Acids Res* 29: 2517 – 2521

Wang Z, Zang C, Cui K, Schones DE, Barski A, Peng W, Zhao K (2009) Genome-wide mapping of HATs and HDACs reveals distinct functions in active and inactive genes. *Cell* 138: 1019 – 1031

Xue Y, Wong J, Moreno GT, Young MK, Cote J, Wang W (1998) NURD, a novel complex with both ATP-dependent chromatin-remodeling and histone deacetylase activities. *Mol Cell* 2: 851 – 861

Yamada T, Yang Y, Hemberg M, Yoshida T, Cho HY, Murphy JP, Fioravante D, Regehr WG, Gygi SP, Georgopoulos K *et al* (2014) Promoter decommissioning by the NuRD chromatin remodeling complex triggers synaptic connectivity in the mammalian brain. *Neuron* 83: 122 – 134

Yoshida T, Hazan I, Zhang J, Ng SY, Naito T, Snippert HJ, Heller EJ, Qi X, Lawton LN, Williams CJ *et al* (2008) The role of the chromatin remodeler mi-2beta in hematopoietic stem cell self-renewal and multilineage differentiation. *Genes Dev* 22: 1174 – 1189

Zacharioudaki E, Falo Sanjuan J, Bray S (2019) Mi-2/NuRD complex protects stem cell progeny from mitogenic notch signaling. *Elife* 8: e41637

Zhang Y, LeRoy G, Seelig HP, Lane WS, Reinberg D (1998) The dermatomyositis-specific autoantigen Mi2 is a component of a complex containing histone deacetylase and nucleosome remodeling activities. *Cell* 95: 279 – 289

Zhang W, Aubert A, Gomez de Segura JM, Karuppasamy M, Basu S, Murthy AS, Diamante A, Drury TA, Balmer J, Cramard J *et al* (2016) The nucleosome remodeling and deacetylase complex NuRD is built from preformed catalytically active sub-modules. *J Mol Biol* 428: 2931 – 2942

Ziffra RS, Kim CN, Ross JM, Wilfert A, Turner TN, Haeussler M, Casella AM, Przytycki PF, Keough KC, Shin D *et al* (2021) Single-cell epigenomics reveals mechanisms of human cortical development. *Nature* 598: 205 – 213

License: This is an open access article under the terms of the Creative Commons Attribution License, which permits use, distribution and reproduction in any medium, provided the original work is properly cited.

Expanded View Figures

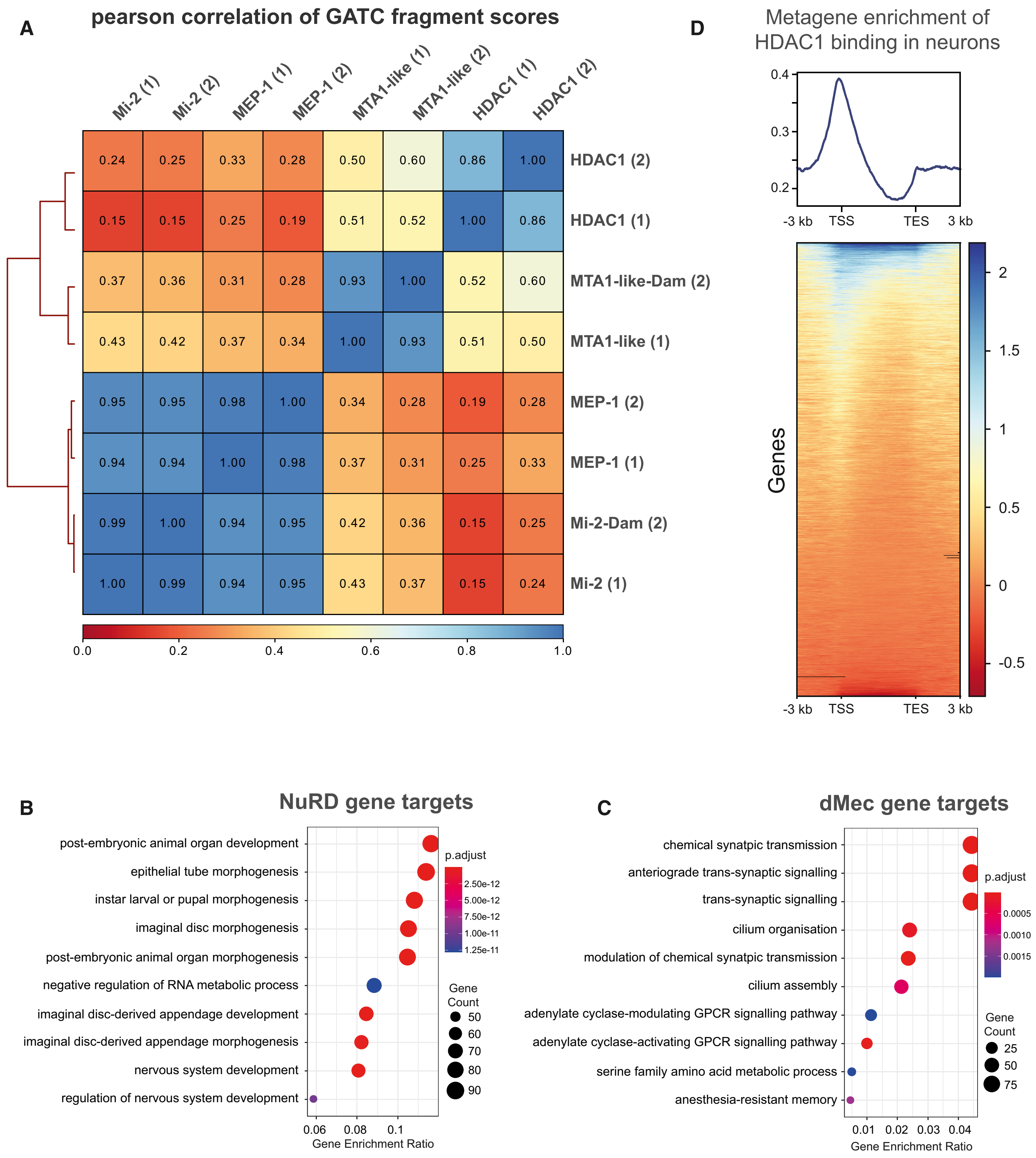

Figure EV1.

Figure EV1. Genomic binding of NuRD components.

A Correlation between NuRD subunits (including replicates) in larval neurons (*elav*-GAL4). Very strong correlations are seen between Mi-2 and MEP-1 binding while the correlation between Mi-2/MEP-1 and either MTA-1like or HDAC1 is relatively lower. Good correlations are exhibited between replicates for each subunit (spearman's correlation r^2 = 0.85–0.95).

B GO analysis of NuRD gene targets.

C GO analysis of dMec gene targets genes.

D Metagene analysis of HDAC1 binding. TSS is Transcriptional Start Site, and TES is Transcriptional End Site.

Figure EV2. RNA-seq quality control and differentially expressed genes.

A Heatmap showing Pearson correlation of RNA-seq reads between biological replicates and control and experimental groups. Very strong correlations are observed between replicates ($R^2 > 0.97$) with slightly lower correlations between knockdown and controls reflecting the changes in *Mi-2* RNAi transcriptome.

B Genome browser tracks indicating RNA-seq read coverage at the *Mi-2* locus. *Mi-2* reads are clearly depleted in *Mi-2 RNAi* compared with *elav*-GAL4 × *mCherry RNAi* controls (adjusted P value $< 2.2 \times 10^{-227}$).

C Heatmap showing relative changes in normalised gene expression for the thirty most significantly upregulated genes.

D Heatmap showing relative changes in normalised gene expression for the 30 most significantly down-regulated genes.

E, F Read coverage for (E) *gammaTub37C* and (F) *RpS5b* loci, which are non-CNS genes ectopically expressed in *Mi-2* knockdown.

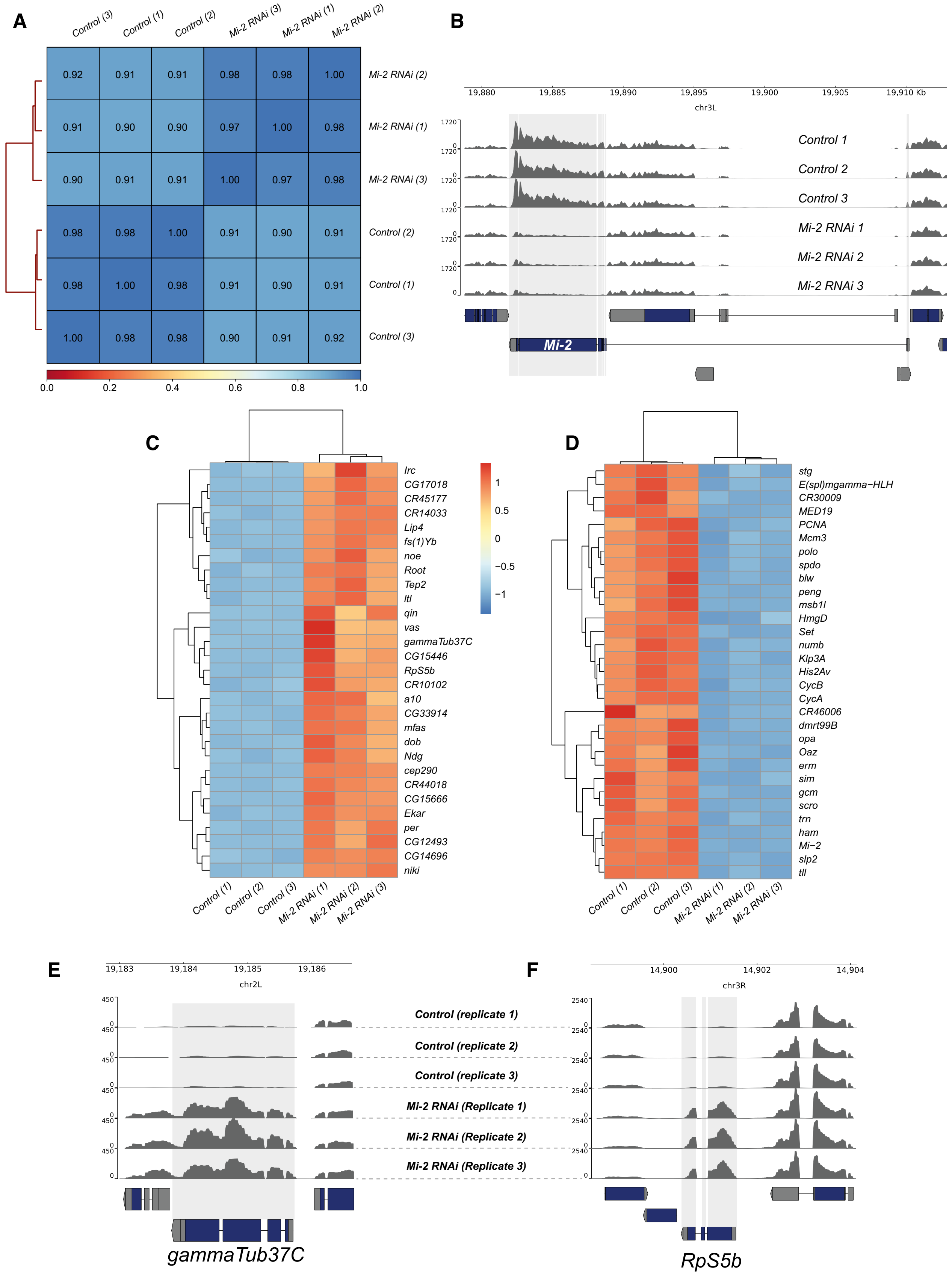
A
Control (3)
Control (1)
Control (2)
Mi-2 RNAi (3)
Mi-2 RNAi (1)
Mi-2 RNAi (2)
B
chr3L
Control 1
Control 2
Control 3
Mi-2 RNAi 1
Mi-2 RNAi 2
Mi-2 RNAi 3
Mi-2
C
D
E
chr2L
Control (replicate 1)
Control (replicate 2)
Control (replicate 3)
Mi-2 RNAi (Replicate 1)
Mi-2 RNAi (Replicate 2)
Mi-2 RNAi (Replicate 3)
gammaTub37C
F
chr3R
RpS5b

Figure EV2.

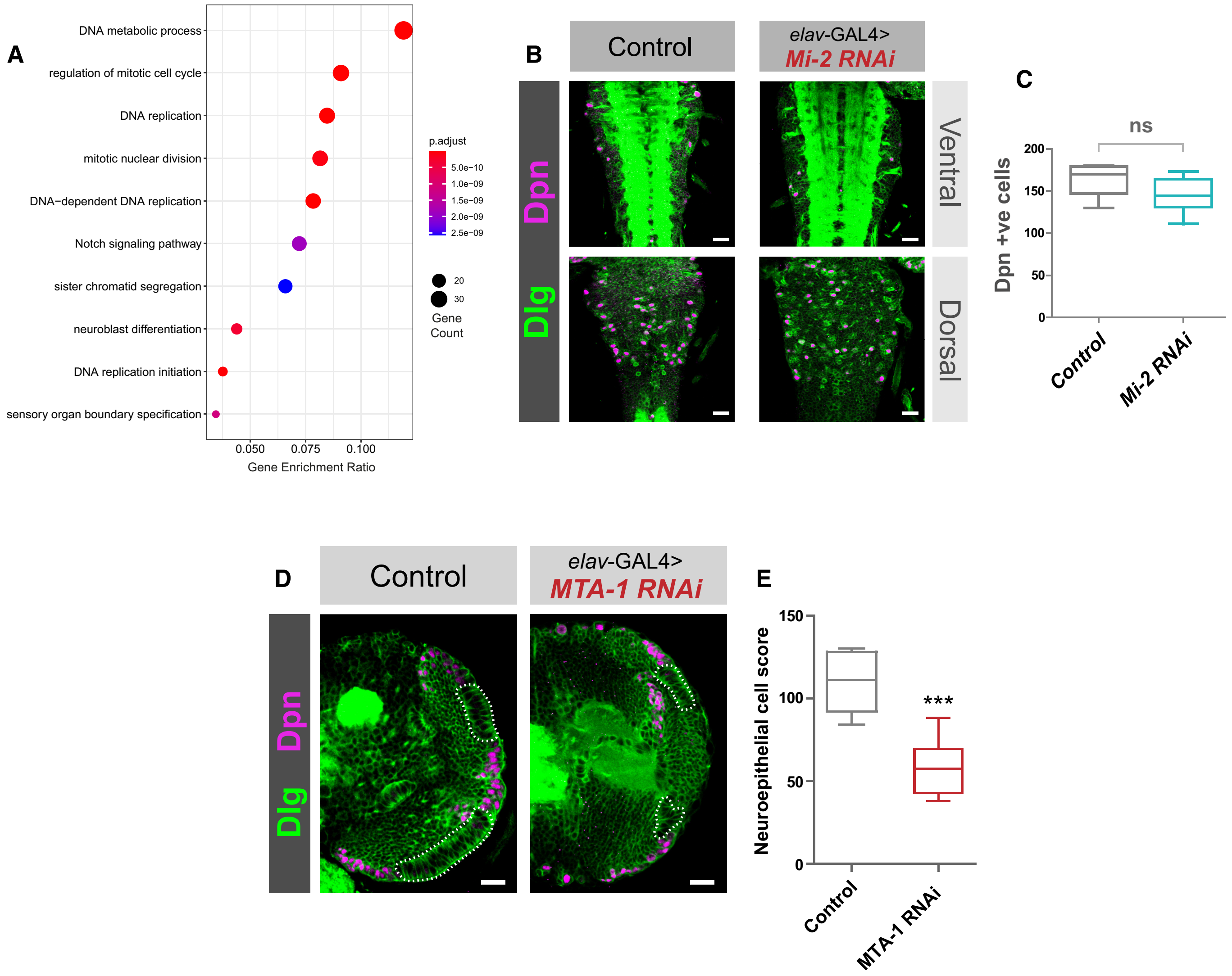

Figure EV3. ***NuRD* knockdown larval brain phenotypes.**

A GO analysis of downregulated genes.

B *Mi-2* knockdown does not affect NSC numbers in the VNC. (B) Example images from ventral and dorsal sections of the VNC. Scale bars = 20 µm.

C Quantification of Dpn-positive cells (no significant difference). Six VNCs measured for each genotype. No significant difference (two-tailed student's *t*-test). Represented as a box plot.

D *MTA1-like* knockdown causes an optic lobe phenotype. (D) Representative images of control (*elav*-GAL4;; *mCherry RNAi*), and *MTA1-like* knockdown in the third instar larval CNS (all scale bars = 20 µm). Discs large (Dlg) = green, Deadpan (Dpn) = magenta. Neuroepithelial cells are highlighted by dashed lines.

E Quantification of optic lobe neuroepithelial cells of genotypes shown in panel A. At least eight brains measured for each genotype. ***$P < 0.001$ (one-tailed student's *t*-test). Represented as a box plot.

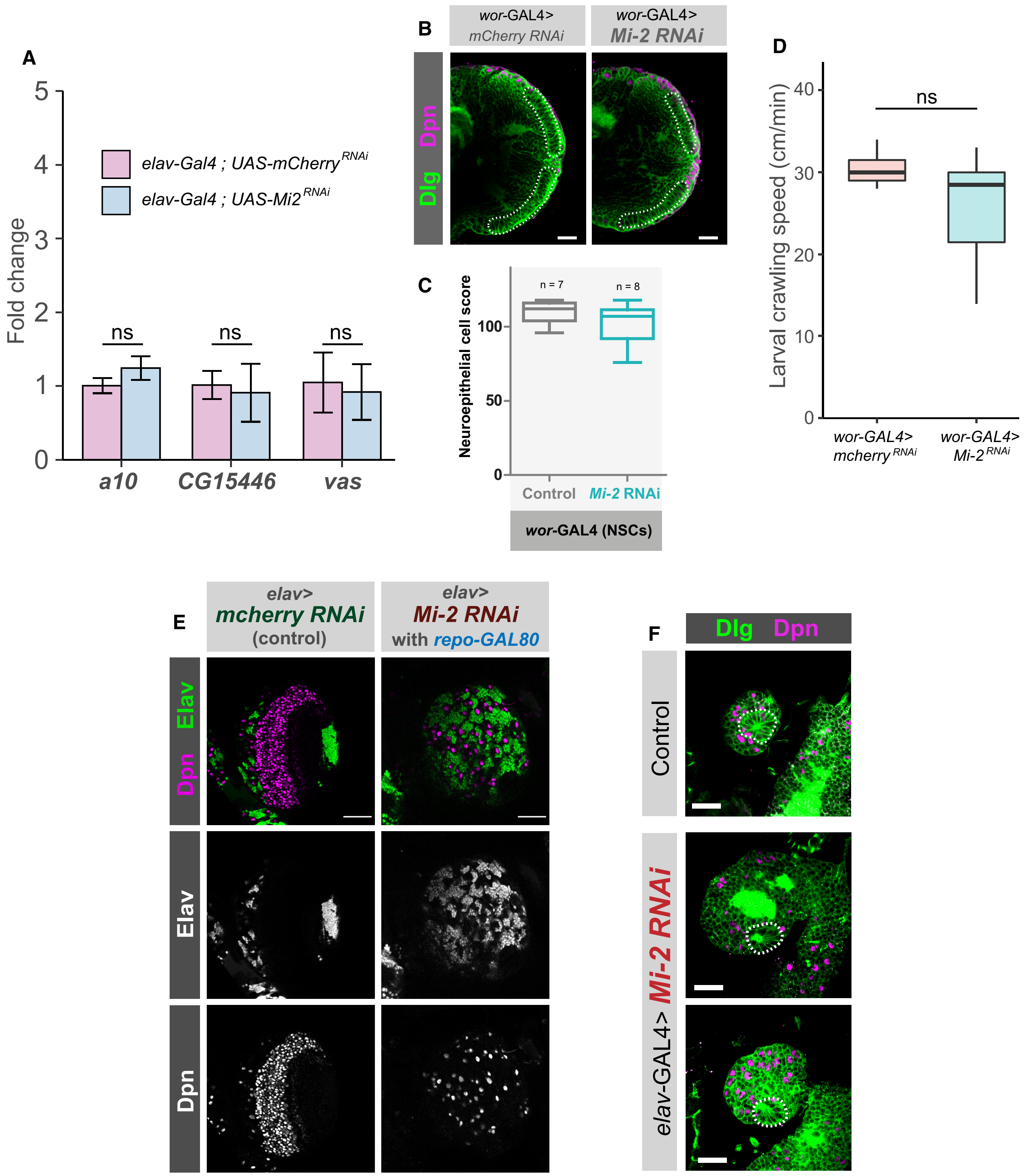
A
Fold change
elav-Gal4 ; UAS-mCherry RNAi
elav-Gal4 ; UAS-Mi2 RNAi
ns
ns
ns
a10
CG15446
vas
B
wor-GAL4> mCherry RNAi
wor-GAL4> Mi-2 RNAi
Dlg Dpn
C
Neuroepithelial cell score
n = 7
n = 8
Control
Mi-2 RNAi
wor-GAL4 (NSCs)
D
Larval crawling speed (cm/min)
ns
wor-GAL4> mcherry RNAi
wor-GAL4> Mi-2 RNAi
E
elav> mcherry RNAi (control)
elav> Mi-2 RNAi with repo-GAL80
Dpn Elav
Elav
Dpn
F
Dlg Dpn
Control
elav-GAL4> Mi-2 RNAi

Figure EV4.

◀ **Figure EV4. Differential requirements of Mi-2 during neuronal differentiation.**

- A Knockdown of *Mi-2* in adult brain neurons does not cause ectopic expression of genes. qPCR measurement of gene expression in adult heads. *Mi-2*^{RNAi} is induced for 24 h using GAL80^{ts}. No significant changes in expression of either *vas*, *a10* or *CG15446* were observed (one-tailed students *t*-test, represented as mean ± SEM.). Three biological replicates per genotype.
- B Representative images of optic lobes for control and *Mi-2* knockdown in NSCs.
- C Quantification of optic lobe neuroepithelial cells of genotypes shown in panel B ($n >= 7$ for each group). Represented as a box plot. No significant difference found (one-tailed student's *t*-test).
- D Larval crawl speed (cm/min) in *wor*-GAL4 × *Mi-2* RNAi and *wor*-GAL4 × *mCherry* RNAi controls. No significant changes in crawl speed were observed (two-tailed students *t*-test, $n = 10$ animals per genotype). Represented as a box plot.
- E Optic lobe phenotype is not due to *Mi-2* knockdown in glial cells. Optic lobe of third instar larvae in which *repo*-GAL80 represses glial GAL4 expression. Loss of neuroepithelial cells is still observed as in *elav*-GAL4; *Mi-2 RNAi* brains. Scale bar = 50 µm.
- F Characteristic rosette structure of the neuroepithelial cells is present in the control first instar optic lobe and in *elav*-GAL4; *Mi-2 RNAi* 1st instar optic lobes. Scale bar = 20 µm.

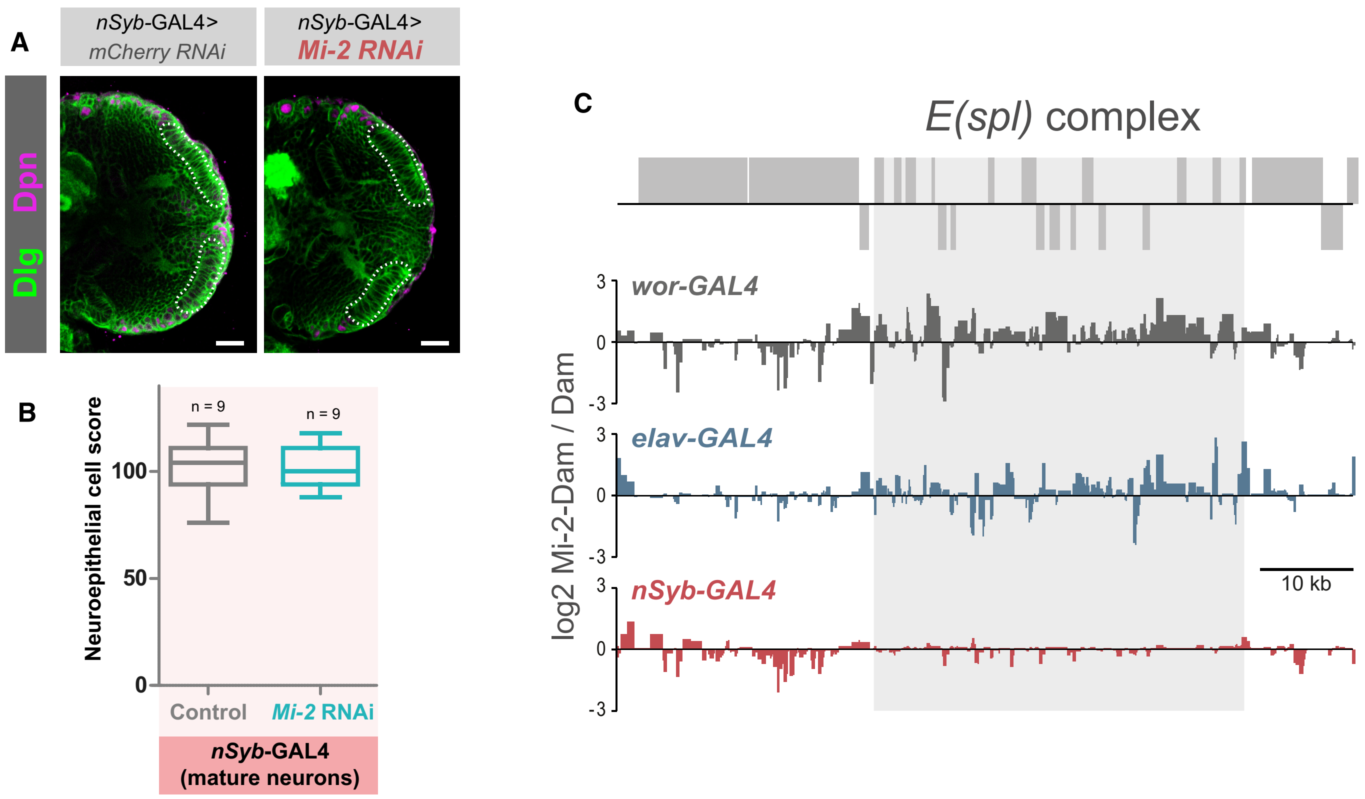

Figure EV5. Differential requirements and binding of Mi-2 during neuronal differentiation.

- A Representative images of optic lobes for control and *Mi-2* knockdown in mature neurons.
- B Quantification of optic lobe neuroepithelial cells of genotypes shown in panel B ($n >= 7$ for each group). Represented as a box plot. No significant difference found (one-tailed student's *t*-test).
- C Mi-2 binding at the *E(spl)* complex is lost in mature neurons. Grey boxes represent genes.

NuRD independent Mi-2 activity represses ectopic gene expression during neuronal maturation.

Gabriel N Aughey‡, Elhana Forsberg†, Krista Grimes†, Shen Zhang†, Tony D Southall*

†Equal contribution

*Correspondence to t.southall@imperial.ac.uk

Department of Life Sciences, Imperial College London, Sir Ernst Chain Building, London, UK

‡Current address: Department of Clinical and Experimental Epilepsy, UCL Queen Square Institute of Neurology, London, United Kingdom.

Appendix information

Contents:

Appendix Figure S1. Tissue enrichment analysis for differentially upregulated genes associated with the "cilium organisation" GO term.

Appendix Figure S2. Expression of differentially expressed genes in other tissues.

Appendix Figure S3. Verification of *Mi-2* and *HDAC1* RNAi lines.

Appendix Figure S4. Pupal lethality in NuRD component RNAi knockdown experiments

Appendix Table S1. Fly stocks used.

Appendix Table S2. DNA oligos used in this study.

Appendix References

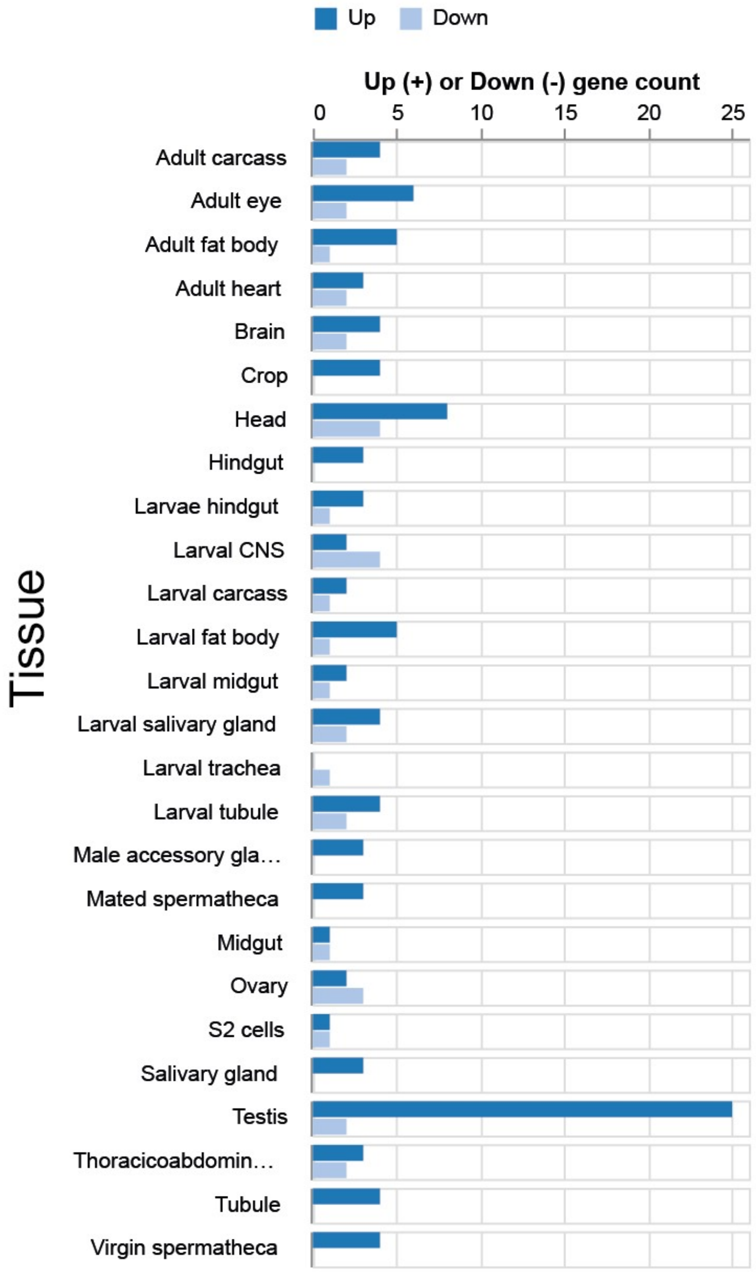

Appendix figure S1. Tissue enrichment analysis for differentially upregulated genes associated with the "cilium organisation" GO term. The majority of these genes are highly upregulated in testis. "Up" and "Down" refer to genes in the set that are significantly up or downregulated in the respective tissue.

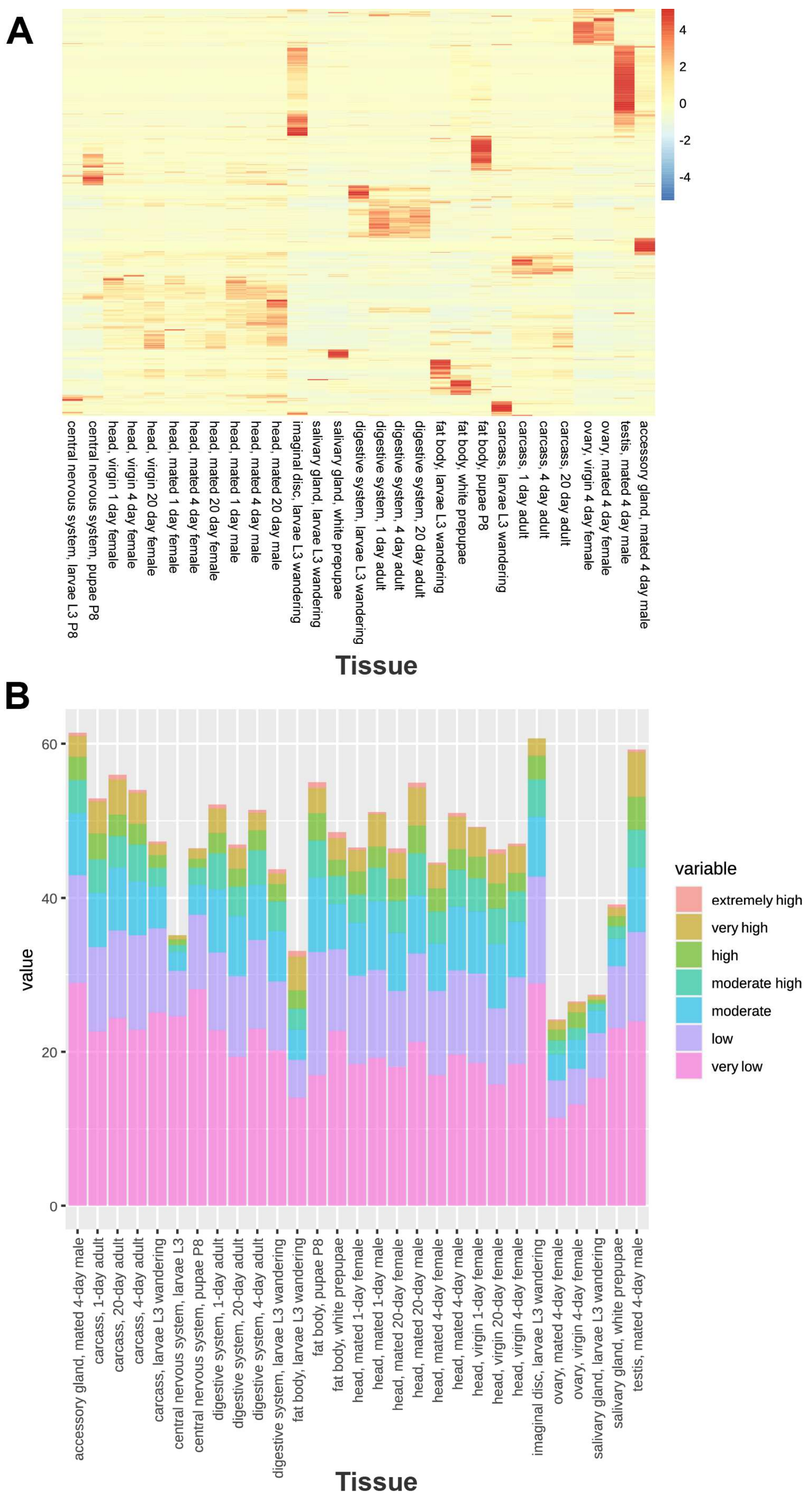

Appendix figure S2. Expression of differentially expressed genes in other tissues. A) Heatmap showing relative expression levels of upregulated genes across all tissues. B) Stacked bar plot showing percentage of upregulated genes that are expressed at different levels in specific tissues. Note that many upregulated genes are expressed at “very low” levels in the fly CNS, whilst upregulated genes are frequently expressed at “moderate” to very high levels in germline and other non-CNS tissues.

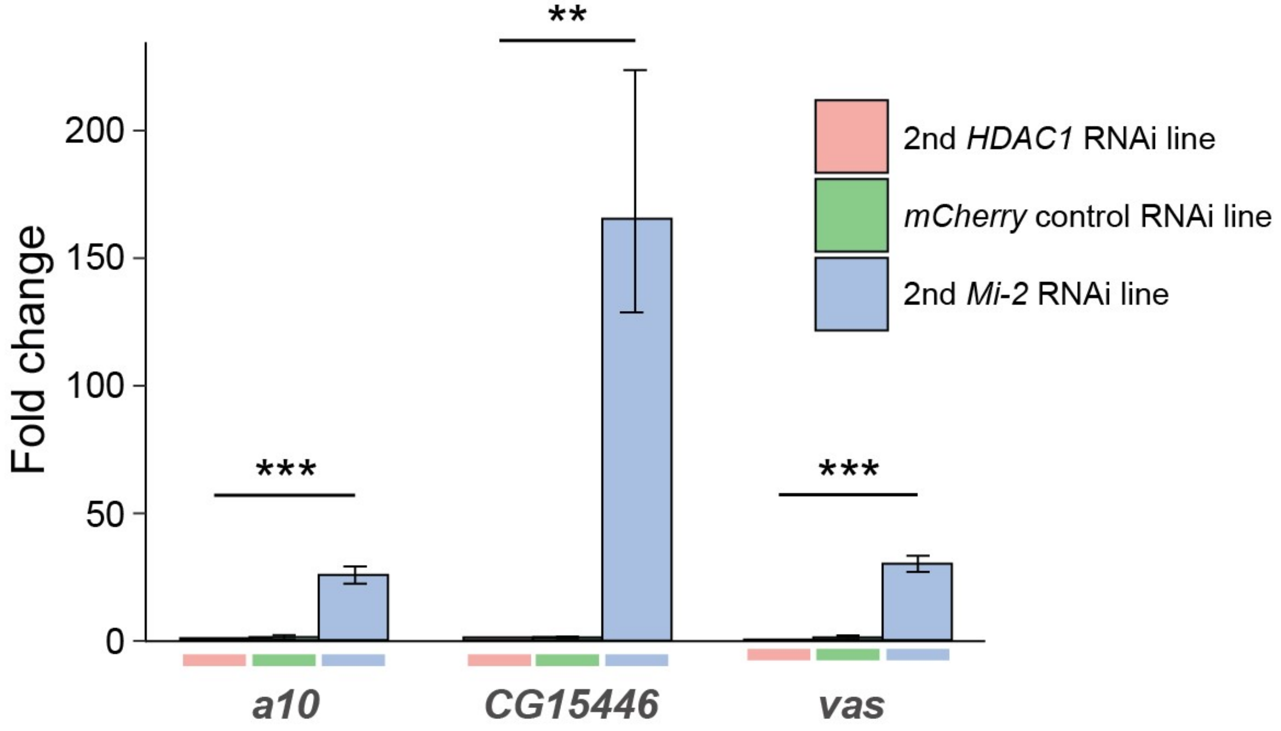

Appendix figure S3. Verification of *Mi-2* and *HDAC1* RNAi lines. qPCR measurement of non-neuronal genes in larval brains using alternative RNAi lines for *Mi-2* and *HDAC* (n = 3). These confirm the results obtained using the original RNAi lines. **P < 0.005, ***P < 0.0005 (one-tailed student's t-test). Error bars show standard deviation. Represented as mean ± SEM.

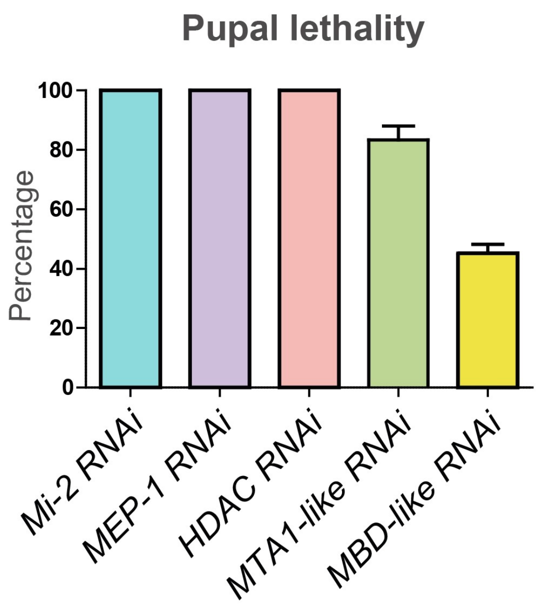

Appendix figure S4. Pupal lethality in NuRD component RNAi knockdown experiments. Percentage of pupae that die when NuRD subunits are knocked down in neurons (driven by *elav*-GAL4) during development. 3 independent experiments were performed for each genotype.

Appendix Table S1. Fly stocks used.

Genotype	Source	Reference	Description
y^1w^*; Mi{PT-GFSTF.1}Mi-2$^{MI07934-GFSTF.1}$ Su(Tpl)$^{MI07934-GFSTF.1-X}$/TM6C, Sb^1 Tb^1	BDSC #63188	(Nagarkar-Jaiswal et al., 2015)	Mi-2 GFP trap
y^1 w^*; Mi{PT-GFSTF.1}MTA1-like$^{MI01790-GFSTF.1}$	BDSC #63161	(Nagarkar-Jaiswal et al., 2015)	MTA GFP trap
UAS-LT3-Dam	Andrea Brand	(Southall et al., 2013)	
UAS- Dam-MEP-1	This study		
UAS-Dam-Mi-2	This study		
UAS-MTA1-like-Dam	This study		flyORF TaDa
UAS-HDAC-Dam	This study		flyORF TaDa
wor-GAL4; *tub*-GAL80^{ts}	Andrea Brand	(Albertson et al., 2004)	
wor-GAL4	Andrea Brand	(Albertson et al., 2004)	
elav-GAL4*;* *tub*-GAL80^{ts}	Andrea Brand		
nSyb-GAL4	BDSC #51941		
hs-flp; UAS-flyORF.TaDa	BDSC #91637	(Aughey et al., 2021)	
UAS-HDAC1	FlyORF F000675	(Bischof et al., 2013)	
UAS-MTA1-like	FlyORF F001892	(Bischof et al., 2013)	
UAS-Mi2 RNAi	BDSC #51774		
UAS-Mi2 RNAi (2nd line)	BDSC #35398		
UAS-MTA1-like RNAi	BDSC #34624		
UAS-HDAC1 RNAi	BDSC #33725		
UAS-HDAC1 RNAi (2nd line)	BDSC #34846		
UAS-MEP-1 RNAi	BDSC #62180	(Perkins et al., 2015)	
UAS-MBD-like RNAi	VDRC #9261	(Dietzl et al., 2007)	
mCherry-RNAi (control)	BDSC #35787		
repo-GAL80	Manolis Fanto	(Awasaki et al., 2008)	
elav-GAL4 ; *repo*-GAL80 / *TM6*	This study		

52 **Appendix Table S2. DNA oligos used in this study.**

Primer name	Primer sequence
vas qRT-PCR forward	TGTCTGACGACTGGGATGATG
vas qRT-PCR reverse	ATTTCCTCCTTGGTAGCCGC
CG17566 qRT-PCR forward	TTGGGCCAATGTGGCAATCA
CG17566 qRT-PCR reverse	TGCGATCCTGTCCATCGGT
qin qRT-PCR forward	TCCCTTTCTACTGGGATGCG
qin qRT-PCR reverse	GAGCTGGACTATGGCACACG
RpS5b qRT-PCR forward	ACTACATTGCCGTAAAGGAGAAG
RpS5b qRT-PCR reverse	CATTGGGCCTTGCGGAATC
CG8526 qRT-PCR forward	GATTTGATTGAGTTCTGCCCACT
CG8526 qRT-PCR reverse	CTTGTATGTCTTTCCCACTTCGT
CG15446 qRT-PCR forward	CGGAAACGGCTACCCATGT
CG15446 qRT-PCR reverse	CCCCGACTTACCTTCATCTTCG
a10 qRT-PCR forward	ATCCTTAACCAAGAGCGACTGT
a10 qRT-PCR reverse	TCACCTTTTCAGCACCATACC
RpL4 qRT-PCR forward	TCCACCTTGAAGAAGGGCTA
RpL4 qRT-PCR reverse	TTGCGGATCTCCTCAGACTT
*Dam-Mi2*_forward	gaagaggatctggccggcgcagatctgcggATGGCATCGGAGGAAGAGAATGACGATAAT
*Dam-Mi2*_reverse	aagtaaggttccttcacaaagatcctctagCTAGACGCCGGAATTATTCGATAGCTGGCC
*Dam-MEP-1*_forward	gaagaggatctggccggcgcagatctgcggATGACTGAAGTTGATGTCGTTTTGCCGGAG
*Dam-MEP-1*_reverse	aagtaaggttccttcacaaagatcctctagTTAATCTATGACATGACTCTCCATATTTG

Appendix References

Albertson, R., Chabu, C., Sheehan, A., and Doe, C.Q. (2004). Scribble protein domain mapping reveals a multistep localization mechanism and domains necessary for establishing cortical polarity. *J Cell Sci* 117, 6061-6070.

Aughey, G.N., Delandre, C., McMullen, J.P.D., Southall, T.D., and Marshall, O.J. (2021). FlyORF-TaDa allows rapid generation of new lines for in vivo cell-type-specific profiling of protein-DNA interactions in Drosophila melanogaster. *G3 (Bethesda)* 11.

Awasaki, T., Lai, S.L., Ito, K., and Lee, T. (2008). Organization and postembryonic development of glial cells in the adult central brain of Drosophila. *J Neurosci* 28, 13742-13753.

Bischof, J., Bjorklund, M., Furger, E., Schertel, C., Taipale, J., and Basler, K. (2013). A versatile platform for creating a comprehensive UAS-ORFeome library in Drosophila. *Development* 140, 2434-2442.

Dietzl, G., Chen, D., Schnorrer, F., Su, K.C., Barinova, Y., Fellner, M., Gasser, B., Kinsey, K., Oppel, S., Scheiblauer, S., *et al.* (2007). A genome-wide transgenic RNAi library for conditional gene inactivation in Drosophila. *Nature* 448, 151-156.

Nagarkar-Jaiswal, S., Lee, P.T., Campbell, M.E., Chen, K., Anguiano-Zarate, S., Gutierrez, M.C., Busby, T., Lin, W.W., He, Y., Schulze, K.L., *et al.* (2015). A library of MiMICs allows tagging of genes and reversible, spatial and temporal knockdown of proteins in Drosophila. *Elife* 4.

Perkins, L.A., Holderbaum, L., Tao, R., Hu, Y., Sopko, R., McCall, K., Yang-Zhou, D., Flockhart, I., Binari, R., Shim, H.S., *et al.* (2015). The Transgenic RNAi Project at Harvard Medical School: Resources and Validation. *Genetics* 201, 843-852.

Southall, T.D., Gold, K.S., Egger, B., Davidson, C.M., Caygill, E.E., Marshall, O.J., and Brand, A.H. (2013). Cell-type-specific profiling of gene expression and chromatin binding without cell isolation: assaying RNA Pol II occupancy in neural stem cells. *Dev Cell* 26, 101-112.
