# Supplementary material for: NuRD‐independent Mi‐2 activity represses ectopic gene expression during neuronal maturation
Source: EMBO Rep. 2023 Feb 1;24(4):e55362. doi: 10.15252/embr.202255362 (PMC10074086; doi:10.15252/embr.202255362)
Supplement: Supplementary file 4 [file EMBR-24-e55362-s003.pdf]

EMBO reports

NuRD independent Mi-2 activity represses ectopic gene expression during neuronal maturation

Gabriel Aughey, Elhana Forsberg, Krista Grimes, Shen Zhang, and Tony Southall
DOI: 10.15252/embr.202255362

Corresponding author(s): Tony Southall (t.southall@imperial.ac.uk)

Review Timeline:

Submission Date:	6th May 22
Editorial Decision:	30th Jun 22
Revision Received:	16th Nov 22
Editorial Decision:	3rd Jan 23
Revision Received:	10th Jan 23
Accepted:	16th Jan 23

Editor: Esther Schnapp

Transaction Report:

(Note: With the exception of the correction of typographical or spelling errors that could be a source of ambiguity, letters and reports are not edited. Depending on transfer agreements, referee reports obtained elsewhere may or may not be included in this compilation. Referee reports are anonymous unless the Referee chooses to sign their reports.)

1st Editorial Decision 30th Jun 2022

Dear Dr. Southall,

Thank you for the submission of your manuscript to EMBO reports. We have now received the full set of referee reports that is pasted below.

As you will see, the referees acknowledge that the findings are potentially interesting. However, they also have several suggestions for how the study should be improved. I think all suggestions should be addressed, but please let me know if you disagree, and we can discuss the revisions further.

I would thus like to invite you to revise your manuscript with the understanding that the referee concerns must be fully addressed and their suggestions taken on board. Please address all referee concerns in a complete point-by-point response. Acceptance of the manuscript will depend on a positive outcome of a second round of review. It is EMBO reports policy to allow a single round of major revision only and acceptance or rejection of the manuscript will therefore depend on the completeness of your responses included in the next, final version of the manuscript.

We realize that it is difficult to revise to a specific deadline. In the interest of protecting the conceptual advance provided by the work, we recommend a revision within 3 months (30th Sep 2022). Please discuss the revision progress ahead of this time with the editor if you require more time to complete the revisions.

You can either publish the study as a short report or as a full article. For short reports, the revised manuscript should not exceed 27,000 characters (including spaces but excluding materials & methods and references) and 5 main plus 5 expanded view figures. The results and discussion sections must further be combined, which will help to shorten the manuscript text by eliminating some redundancy that is inevitable when discussing the same experiments twice. For a normal article there are no length limitations, but it should have more than 5 main figures and the results and discussion sections must be separate. In both cases, the entire materials and methods must be included in the main manuscript file.

IMPORTANT NOTE: we perform an initial quality control of all revised manuscripts before re-review. Your manuscript will FAIL this control and the handling will be DELAYED if the following APPLIES:
1) A data availability section providing access to data deposited in public databases is missing. If you have not deposited any data, please add a sentence to the data availability section that explains that.
2) Your manuscript contains statistics and error bars based on n=2. Please use scatter blots in these cases. No statistics should be calculated if n=2.

When submitting your revised manuscript, please carefully review the instructions that follow below. Failure to include requested items will delay the evaluation of your revision.

1) a .docx formatted version of the manuscript text (including legends for main figures, EV figures and tables). Please make sure that the changes are highlighted to be clearly visible.

2) individual production quality figure files as .eps, .tif, .jpg (one file per figure). See https://wol-prod-cdn.literatumonline.com/pb-assets/embo-site/EMBOPress_Figure_Guidelines_061115-1561436025777.pdf for more info on how to prepare your figures.

3) We replaced Supplementary Information with Expanded View (EV) Figures and Tables that are collapsible/expandable online. A maximum of 5 EV Figures can be typeset. EV Figures should be cited as 'Figure EV1, Figure EV2" etc... in the text and their respective legends should be included in the main text after the legends of regular figures.

- For the figures that you do NOT wish to display as Expanded View figures, they should be bundled together with their legends in a single PDF file called *Appendix*, which should start with a short Table of Content. Appendix figures should be referred to in the main text as: "Appendix Figure S1, Appendix Figure S2" etc. See detailed instructions regarding expanded view here: <https://www.embopress.org/page/journal/14693178/authorguide#expandedview>

- Additional Tables/Datasets should be labeled and referred to as Table EV1, Dataset EV1, etc. Legends have to be provided in a separate tab in case of .xls files. Alternatively, the legend can be supplied as a separate text file (README) and zipped together with the Table/Dataset file.

4) a .docx formatted letter INCLUDING the reviewers' reports and your detailed point-by-point responses to their comments. As part of the EMBO Press transparent editorial process, the point-by-point response is part of the Review Process File (RPF), which will be published alongside your paper.

5) a complete author checklist, which you can download from our author guidelines <https://www.embopress.org/page/journal/14693178/authorguide>. Please insert information in the checklist that is also reflected in the manuscript. The completed author checklist will also be part of the RPF.

6) Please note that all corresponding authors are required to supply an ORCID ID for their name upon submission of a revised manuscript (<https://orcid.org/>). Please find instructions on how to link your ORCID ID to your account in our manuscript tracking system in our Author guidelines
<https://www.embopress.org/page/journal/14693178/authorguide#authorshipguidelines>

7) Before submitting your revision, primary datasets produced in this study need to be deposited in an appropriate public database (see https://www.embopress.org/page/journal/14693178/authorguide#datadeposition). Please remember to provide a reviewer password if the datasets are not yet public. The accession numbers and database should be listed in a formal "Data Availability" section placed after Materials & Method (see also https://www.embopress.org/page/journal/14693178/authorguide#datadeposition). Please note that the Data Availability Section is restricted to new primary data that are part of this study. * Note - All links should resolve to a page where the data can be accessed. *
If your study has not produced novel datasets, please mention this fact in the Data Availability Section.

8) We would also encourage you to include the source data for figure panels that show essential data. Numerical data should be provided as individual .xls or .csv files (including a tab describing the data). For blots or microscopy, uncropped images should be submitted (using a zip archive if multiple images need to be supplied for one panel). Additional information on source data and instruction on how to label the files are available at
<https://www.embopress.org/page/journal/14693178/authorguide#sourcedata>.

9) Our journal also encourages inclusion of *data citations in the reference list* to directly cite datasets that were re-used and obtained from public databases. Data citations in the article text are distinct from normal bibliographical citations and should directly link to the database records from which the data can be accessed. In the main text, data citations are formatted as follows: "Data ref: Smith et al, 2001" or "Data ref: NCBI Sequence Read Archive PRJNA342805, 2017". In the Reference list, data citations must be labeled with "[DATASET]". A data reference must provide the database name, accession number/identifiers and a resolvable link to the landing page from which the data can be accessed at the end of the reference. Further instructions are available at https://www.embopress.org/page/journal/14693178/authorguide#referencesformat

10) Regarding data quantification (see Figure Legends: https://www.embopress.org/page/journal/14693178/authorguide#figureformat)

The following points must be specified in each figure legend:

- the name of the statistical test used to generate error bars and P values,

- the number (n) of independent experiments (please specify technical or biological replicates) underlying each data point,

- the nature of the bars and error bars (s.d., s.e.m.),

- If the data are obtained from n {less than or equal to} 2, use scatter blots showing the individual data points.

Discussion of statistical methodology can be reported in the materials and methods section, but figure legends should contain a basic description of n, P and the test applied.

- Please also include scale bars in all microscopy images.

11) The journal requires a statement specifying whether or not authors have competing interests (defined as all potential or actual interests that could be perceived to influence the presentation or interpretation of an article). In case of competing interests, this must be specified in your disclosure statement. Further information: https://www.embopress.org/competing-interests

We would also welcome the submission of cover suggestions, or motifs to be used by our Graphics Illustrator in designing a cover.

As part of the EMBO publication's Transparent Editorial Process, EMBO reports publishes online a Review Process File (RPF) to accompany accepted manuscripts. This File will be published in conjunction with your paper and will include the referee reports, your point-by-point response and all pertinent correspondence relating to the manuscript.

You are able to opt out of this by letting the editorial office know (emboreports@embo.org). If you do opt out, the Review Process File link will point to the following statement: "No Review Process File is available with this article, as the authors have chosen not to make the review process public in this case."

I look forward to seeing a revised form of your manuscript when it is ready. Please use this link to submit your revision:

https://embor.msubmit.net/cgi-bin/main.plex

Yours sincerely,

Esther Schnapp, PhD
Senior Editor
EMBO reports

Referee #1:

In this study, the authors investigate the role of Mi-2 during the course of neuronal differentiation. Mi-2 is a core member of the chromatin remodeling complex NuRD. In Drosophila, some studies have proposed that this highly conserved factor may also be present in another complex called dMec. The precise role of Mi-2, as a component of the NuRD or dMec complexes, in the developing nervous system is not fully understood. Here the authors used Drosophila genetics and TaDa to investigate the function and the genome-wide binding profile of Mi-2 during neuronal differentiation. They show that Mi-2 acts in two distinct complexes during this process to regulate different target genes. In particular, Mi-2 associates with MEP-1 to form the dMec complex and repress a set of germline genes during the early stages of neuronal maturation. Therefore Mi-2 has an important role during neurogenesis, outside of its canonical role in the NuRD complex, to maintains the repression of inappropriate genes. This study elegantly combines genetics and chemistry in Drosophila to reveal new insights about the dynamic and specific roles of a key chromatin protein during neurogenesis. There are a few points that need to be clarified before publication.

1. The phenotype caused by the loss of Mi-2 and NuRD complex genes in the optic lobe is minimally described. The authors suggests that the underlying cause of the smaller optic lobe could be the loss of the neuroepithelium. Can you show the neuroepithelium more clearly in all conditions? It would help to determine how the different components of the complex contribute to this precise phenotype.

2. The authors mention that knockdown of MBD-like and HADC1 leads to similar phenotypes as Mi-2 knockdown in the optic lobe. But the phenotype in Fig 3A appears somehow mild for the MBD-like knockdown with medulla NBs being still present. Is the neuroepithelium still present? What is your validation that the knockdown is efficient?

3. Throughout the manuscript, the authors used three GAL4 drivers presumably allowing the expression of various RNAi transgenes in distinct cell types (NBs, all neurons, mature neurons). However, they don't clearly show where these drivers are expressed. In particular, Elav-GAL4 is known to be also expressed in NBs in the central brain. Is it also expressed in optic lobe NBs? How about its expression in the neuroepithelium? A leaky activity of this driver in the neuroepithelium could explain the small optic lobe phenotype when crossed with the Mi-2-RNAi line and provide an alternative explanation to the "non-cell autonomous" one. The "non-cell autonomous" effect could also be due to defective photoreceptor neurons from the retina, in which elav-GAL4 is active and that are known to regulate the growth and differentiation of the neuroepithelium during optic lobe development.

4. The germline gene Vasa is up-regulated upon Mi-2-RNAi when using wor-GAL4 or elav-GAL4 but not nSyb-GAL4 suggesting that derepression occurs in NBs and or immature neurons. Can you validate this cell type-specific phenotype using immunostainings ? There are anti-Vasa antibodies available at DSHB.

5. The qPCR experiments show that Vasa is also up-regulated in progenitors and immature neurons (but not mature neurons) but upon loss of Mi-2. Does this correlates with an absence or presence of Mi-2 binding on the gene in these different cell types?

6. Because inactivation of members of the NuRD complex leads to similar defects without inducing ectopic expression of germline genes, the authors conclude that "the ectopic regulation of gene is independent of NuRD/histone deacetylation, and does not contribute to optic lone developmental defects." I think this conclusion is overstated as it could be that germline genes also contribute to the phenotype (what happens if you mis-express them in neurons?). I would suggest rephrasing in something like: "The ectopic up-regulation of germline genes is not necessary for causing the optic lobe phenotype in the larva."

7. The TaDa experiments show that Mi-2 and Mep-1 have the same binding sites therefore suggesting that they belong to the same complex. If true, their knockdown should lead to the same phenotype. What is the phenotype of Mep-1 knockdown in the optic lobe? Do you see upregulation of germline genes?

Figures:
2B: please indicate the genotype for each track
3A: there are mistakes in the panels for Elav and Dpn
5: the first panel is not numbered and has no legend
The title of the panel 5B is confusing. Why is dMec mentioned here?

The RNAi flystocks used to knock-down MBD-like are not referenced in Table S1. Only one RNAi stock for the knock-down of Mi-2 is mentioned while the authors claim to have used two lines.

The "vas" abbreviation is not specified in the text

Referee #2:

Summary
Aughey et al. have studied the role of the protein Mi-2 in neuronal differentiation in Drosophila. The authors show that Mi-2 protein is expressed in all stages of neurogenesis including the Neuroblasts, GMCs, and postmitotic neurons. They KD Mi-2 with the elav-Gal4 driver and revealed a decrease in fly viability and larval mobility. These phenotypes are linked to a spatio-temporal de-repression in the CNS of CNS genes and more strikingly of non-CNS genes. They also characterized the optic lobe phenotype in KD Mi-2 flies. The phenotype of the optic lobe is characterized by a reduction of its size correlated with a reduction of the number of proliferative cells (the reduction of post mitotic neurons is not clearly mentioned). The study of the function of components of the NuRD complex such as MBD-like, MTA1-like and HDAC1 revealed that the function of Mi-2 on gene repression seems independent of the NuRD complex. However the individual KD of MBD-like, MTA1-like and HDAC1 induce same optic lobe defect as Mi-2. These results suggest that Mi-2 might act through another complex to repress genes. To address this possibility, they performed TaDa on Mi-2, components of NuRD complex and MEP-1, a known protein associate of the dMEC complex that includes Mi-2. The results show that different complexes bind to the DNA, including MEP-1 and Mi-2 which have the closest similarity in their binding profile. The analysis of the binding site and the chromatin state suggest that NuRD mediates activation, while Mi2/dMEC facilitate silencing. Then Aughey et al. used different drivers to knock down Mi-2 in the NB and mature neurons and determined the impact of such manipulation on survival, optic lobe structure and gene regulation. They conclude that Mi-2 has a different function in gene regulation in NB vs immature neurons vs differentiated neurons. The comparison of Mi TaDa experiments in NBs, immature neurons vs differentiated neurons show variation on Mi-2 binding in these different types of cells that could explain the different functions of mi-2 in different cell subtypes.

In my opinion the main message of the paper that is supported by sufficient data, is that Mi2 represses the expression of several non-neuronal genes in the CNS independently of the NurD complex. This message is of broad interest.

However some conclusions put forth by the authors are not supported by strong experimental results and are in my opinion too speculative. The main conclusion proposed which must be reevaluated or supported by new experiments is:
Mi-2 has different functions (fly survival/optic lobe structure/gene regulation) in NB/GMC vs immature neurons vs immature MNs. The genetic experiments are not strong enough to support such conclusion. Below more factual arguments concerning this point and some minor points:

Related to figure 1.
Minor point: The authors claims that the mi-2::GFP is expressed in the neuroblasts, GMCs and post-mitotic neurons. The expression in NBs is convincing, since these cells can be recognized by their size. The expression in the neurons seems also convincing since many cells expressed the GFP. However it difficult to know if all post-mitotic neurons expressed mi-2::GFP and it is impossible to know if mi-2::GFP is expressed in the GMCs. If the authors want to claim that all cell types in the CNS expressed mi-2::GFP, I recommend that the authors perform co-staining with Elav, Dpn and Repo. Postmitotic neurons will be (Elav+), neuroblasts (Dpn +), glia (repo +) and GMCs (Elav -, Dpn and Repo-).

Minor point: The authors also say (page 3) 'with progeny cells adjacent to NSCs exhibiting particularly high expression'. It is true that the GFP intensity in these cells (Figure 1 B) is high compared to the neuroblast. I am curious to know why the authors make this point. The expression in the NB is weaker but this could simply be the consequence of the size of the NB (that in the figure is in the interphase phase) that can make mi-GFP protein more diluted.

Minor point: The authors wrote that Mi-2 activity in neurons is required throughout development at all life-stages for normal CNS function'. This sentence is a bit too 'strong' since Elav is also expressed in the PNS.

Major point: The authors wrote in the title of the paragraph (page 3), that Mi-2 is required in neurons for survival and larval locomotion. The driver used, elav-gal4, has been and it is still sometime used as a neuronal postmitotic driver. However this driver is also expressed in the NB, GMCs and some glia (Berger et al., 2017). elav-gal4 can not be used to distinguish the function of Mi-2 in NBs/GMCs vs postmitotic neurons vs glia.

Related to figure 2.
Minor points: The authors wrote we performed RNA-seq on larval brains. The sentence should be (unless I missed something in the materials and methods): we performed RNA-seq on larval CNS (which includes the VNC).

Related to Figure 3.
Minor points: The DPN and Elav panel have been inverted in control vs Mi-2 RNAi. The dashed lines do not only label NBs as indicated in the figure legend. I think they indicate medulla NB and lamina neurons.

Minor point. The phenotypes induce by Mi-2 KD could be better characterized. Only the surface of the optic lobe is characterized in micrometers. The optic lobe is a 3D structure and should be measured in µm3 and not in µm2. I didn't find any information in Materials and Methods on how the authors quantified the surface. Do they quantify the surface in each focal plane of a full stack?

Major point: The author wrote in page 6: in Mi-2 knockdown resulted in severe optic lobe Mi-2 knockdown brains were smaller, and it was evident that all recognizable optic lobe structures (including inner and outer optic proliferation center as well as differentiating medulla and lamina neurons) were found to be completely absent. It is difficult to see all these phenotypes in a single confocal section in figure 3. Can the authors include confocal sections showing these defects?

Major point: The reduction of the size of the optic lobe is linked to a reduction of the number of NB and other proliferative cells as written in the main text (page 6). This should be quantified in figure 3. Are the number of elav+ cells also reduced? It looks like the number of Elav cells is increased in all experiments of the paper using the elav-gal drivers (main figures and sup figures). This should be clarified in the text and figure 3. In summary, I recommend that the authors quantify the number of cells: NB, vs glia vs neurons)

Minor point: Are the phenotypes described in the optic lobe specific to the optic lobe or is it a more general effect of Mi2 KD?

Major point: More importantly the authors argue that the reduction in the number of NB in elav>Mi2 KD is due to a non-autonomous effect of Mi2. As written above, elav Gal4 is expressed in the NB/GMC (Berger et al., 2017). To make such conclusions the authors should use 'clean' post mitotic drivers such as drivers implicated in neurotransmitter pathway that are expressed only in postmitotic neurons and at early stage.

Major point: The experiment with repo-gal80 is very nice to rule out a function of Mi2 in glia in the maintenance of NB. I suppose the results are described in fig S5 and not S1 (please ref correctly all sup figures, none of them are correctly annotated). Some controls in the sup figures should be added to be sure that Repo-gal80 represses elav-Gal4. Repo-gal80 might not be strong enough to repress elav-gal4 for two reasons: first repo is weakly expressed in some glia sub types such as ensheathing glia or astrocytes, second direct expression of a gene is always weaker than the UAS/GAL4 binary system. To be sure that the experiments were well designed, the authors should check the expression of Mi2-GFP in all glia cell types in the elav>Mi2 KD repo-gal80 animals and add this control in sup Figures.

Minor point: The authors wrote: HDAC1 by RNAi in neurons resulted in lethality during pupal stages similarly to Mi-2. I couldn't fine any graph in the main figure or sup figure supporting this sentence.

Minor point: The authors claim that histone deacetylation thought HDAC1is not required for repression of Mi-2 induced ectopic gene expression in larval. I recommend the authors to be more careful in the sentence, the authors only tested few genes, to write such conclusions the authors should profile the CNS in HDAC1 RNAi.

Minor point. Is the lethality affected in MBD-like RNAi knockdown? This should be commented on in the text.
Related to Figure 4.

Major point: Is the function of Mi-2 in repressing genes going thought the dMEC complex (Mi-2/MEP)? An analysis of the MEP-1 knocking down and the effect on fly survival, lobe optic structure and gene expression should be done.

Related to Figure 4.
Major point: The authors used two drivers wor-gal4 and nsyb-gal4 to temporally define the function of Mi-2 in neuronal differentiation. I have two comments about these drivers. Wor-gal4 is expressed in all NBs but also in the young progeny, especially in the optic lobe, and cannot be considered as a specific NB marker. The graph representing the worl-gal4 expression in Figure 5 is by consequence not correct. This is critical if the authors want to conclude that Mi-2 acts in the young progeny.

The authors also used nSyb-Gal4 to KD Mi-2. I don't understand the goal of the experiment nor the conclusion: whilst Mi-2 is still required in mature neurons, the morphological defects observed are restricted to a critical window of early neuronal maturity. The optic lobe phenotypes described concern the neurons produced during the second wave of neurogenesis. The authors describe a phenotype in a third instar larva and at this stage neurons are still immature and probably most if not all neurons do not express nSyb yet, so RNAi will not be expressed (at this stage it will be expressed only in the neurons produced during embryogenesis). Control experiments of wor-gal4 and nsyb-gal4 UASmcd8::GFP should be done to better define the expression of these drivers and argue against my comments if the authors want to conclude that Mi-2 has different functions at different stages of neuronal differentiation.

Major point: Moreover by using nSyb the authors are not comparing two stages of development but two different population of

neurons, embryonic and larval neurons, born at different stages. If the authors want to analyze the consequence of nsyb-gal4 Mi-2 KD in the optic lobe structure the phenotype should be analysed at later stages (pupa).

Major point: The authors used QPCR to quantify the expression of vasa in wor-gal4 and nsyb-gal4 Mi-D KD. They conclude that Mi-2 represses vasa in NBs. Again wor-gal4 is also expressed in the young progeny. smFISH to quantify vasa in the NB in wor-gal4 Mi-D KD should be done to conclude if Mi-2 plays a role in the NBs. The authors show that no detectable changes were observed in nsyb-gal4 Mi-D KD. As written before maybe it is because nSyb is not yet expressed. Maybe the authors will see changes in vasa expression at later stages when the nSyb driver is highly expressed? The decrease in vasa expression nsyb-gal4 Mi-D KD only shows that Mi-D does not regulate vasa reduction in mature neurons born during embryogenesis which could be the case of adult mature neurons.

Related to Figure 5.
Minor point: Figure 4 A, add in the figure legend (Elav, green, Dpn purple)

Major point: I have the same comment as mentioned before for the use elav- gal4, wor-gal4 and nsyb-gal4 in the TaDa experiment. Elav-gal4 will label embryonic and larval neurons + NB/GMC/glia. wor-gal4: NBs/GMCs/ new born neurons. nsyb-gal4: embryonic born neurons. In conclusion these drivers cannot be used to obtain the temporal and spatial precision that the authors want to achieve.

Related to the discussion
I think the discussion could be more concise and some sentence shaper : We find that in 454
contrast to the abundance of dMec, we find that around a third of Mi-2 peaks intersect with MTA1-like.

Referee #3:

Mi-2 (Chd4) is the catalytic subunit of the NuRD complex, a chromatin remodelling complex whose function is still not fully clear. Originally linked most strongly to gene repression, it is present at many active genes and appears to have a more modulatory role. The components are highly conserved between species, making it extremely valuable to have a detailed genomic profiling from developmental contexts. In addition, previous studies in Drosophola have identified a second Mi-2 containg complex, dMec. Although the existence of the two Mi2 containing complexes was reported previously, the roles of each are quite poorly understood. By mapping the binding sites of Mi-2 and its partners in Drosophila neural lineages, using the Dam-ID approach, Southall and co-workers shed new light on its role in these two complexes. This is the first study that compares their genome wide binding and really demonstrates that two co-exist and have different targets. This is an important contribution.

The quality of the data are very good and the study is thorough. The main criticism is that the structure of the paper is confusing and does not play to its stength- namely the DamID and more specific profiling at different stages. This could be solved with some judicious pruning and restructuring.

Comments:

1. It is valuable to have the initial overview of the phenotypes, but this could be presented in a much more condensed manner to emphasise the key points that Mi-2 (i) represses many non-neural genes (ii) appears to have NuRD dependent and independent activities.
Indeed, they could also consider reorganizing the results, to put the DamID more to the fore-- the phenotypic data would in that case substantiate those very robust results rather than vice versa. They may also be able to tie it up better with the later phenotypic analysis. This would be an advantage for the more general reader.

2. For the interpretations of the phenotypes in the early sections, there is also a concern that much weight is placed on Elav-Gal4 driver being expressed in neurons and not at all in the optic lobe neural stem cells (NBs) e.g. in interpreting the phenotypes as non-autonomous. They should be cautious about this-- have they double checked using a GFP or lacZ reporter that the Gal4 driver is not expressed at low levels in NBs?

3. Figure 4D would benefit from some numbers (as in 5D), it is impossible to guage what the venn diagrams mean without knowing the peak numbers in each case. It is hard to relate the legend to the pie chart in 4F, what is "other" (it would also benefit from numbers).

4. They should include the phenotype form Elav-Gal4 Mep2 RNAi in comparison to the others shown in figure 3, given the overall conclusions about the 2 complexes,

5. it is unclear how these findings relate to the functions of mammalian Mi-2 homologue. I don't find evidence for an analogous complex in the literature, although there is evidence for CHD4 having functions outside of NuRD. It's possible that the more thorough investigation into dMec might spark interest in probing more deeply into other complexes formed by Mi-2 in other organisms. It would be useful for the paper to draw attention to this point, which it does not do at present.

6. The discussion is currently quite long and diffuse. It could be better focussed on the major implications including the point above (5).

Dear Editor,

Please find attached the revised version of our manuscript. We have considered the reviewers comments, and made substantial changes, which we believe has greatly improved the clarity and scientific rigour of our story.

In particular we have changed the focus of our study to highlight the main point regarding the presence of two complexes with distinct activities (as suggested by reviewer 3). In support of this we have also managed to generate new data showing that MEP-1 phenocopies both the optic lobe defect and gene expression phenotype seen with *Mi-2* knockdown.

We have also added extensive new data describing the phenotypes in the larval brain, although we would like to highlight that our intention in including these data is to demonstrate that there are distinct observable phenotypes from the two Mi-2 containing complexes, therefore we refrain from speculating too much or providing a lot more data regarding the aetiology of this phenotype, which we believe to be beyond the scope of this study.

Referee #1:

In this study, the authors investigate the role of Mi-2 during the course of neuronal differentiation. Mi-2 is a core member of the chromatin remodeling complex NuRD. In Drosophila, some studies have proposed that this highly conserved factor may also be present in another complex called dMec. The precise role of Mi-2, as a component of the NuRD or dMec complexes, in the developing nervous system is not fully understood. Here the authors used Drosophila genetics and TaDa to investigate the function and the genome-wide binding profile of Mi-2 during neuronal differentiation. They show that Mi-2 acts in two distinct complexes during this process to regulate different target genes. In particular, Mi-2 associates with MEP-1 to form the dMec complex and repress a set of germline genes during the early stages of neuronal maturation. Therefore Mi-2 has an important role during neurogenesis, outside of its canonical role in the NuRD complex, to maintains the repression of inappropriate genes.
This study elegantly combines genetics and chemistry in Drosophila to reveal new insights about the dynamic and specific roles of a key chromatin protein during neurogenesis. There are a few points that need to be clarified before publication.

1. The phenotype caused by the loss of Mi-2 and NuRD complex genes in the optic lobe is minimally described. The authors suggests that the underlying cause of the smaller optic lobe could be the loss of the neuroepithelium. Can you show the neuroepithelium more clearly in all conditions? It would help to determine how the different components of the complex contribute to this precise phenotype.

In our original submission we had shied away from describing this phenotype in too much detail since we did not want to distract from our main conclusions. However, we agree that more details would be helpful to illustrate the extent of this phenotype. As the reviewer has suggested, we have quantified the neuroepithelial cell numbers to provide a more meaningful description of the optic lobes (Figure 4, Figure EV2 and Appendix figure S11). We have also provided different illustrative sections to communicate this. In producing these new results, we realised that our previous measures of optic lobe size were subject to a great deal of variability due to mounting conditions, so we prefer to omit these in the updated version in favour of the more accurate cell quantitation. We also demonstrate that neuroepithelial cells are present in the first instar stage, indicating that these cells are subsequently lost in the more developed animal.

2. The authors mention that knockdown of MBD-like and HADC1 leads to similar phenotypes as Mi-2 knockdown in the optic lobe. But the phenotype in Fig 3A appears somehow mild for the MBD-like knockdown with medulla NBs being still present. Is the neuroepithelium still present? What is your validation that the knockdown is efficient?

We suspect that the knockdown with the MBD RNAi line is not completely effective. However, since we still observe a phenotype reminiscent of the other NuRD complex members (Figure 4), we are able to draw the conclusion that it is likely acting as part of the complex to confer this phenotype. We would prefer to avoid speculation on the precise nature of MBD-like activity since this is beyond the scope of this study. The role of MBD in *Drosophila* is likely divergent from mammals since it is thought to recognise CpG methylation (which is absent in *Drosophila*). For these reasons, we also include data on the MTA1-like subunit, which produces a phenotype with similar severity to HDAC (Appendix Figure S10), and is known to be a core NuRD component. As the reviewer has mentioned, the phenotype appears to be less severe with both MTA1-like and HDAC1. This could well be as a consequence of inefficient knockdown, however, even if the lnockdown is inefficient, we still see similar phenotypes with all NuRD components tested, we conclude that the NuRD complex rather than dMEC (or as well as), is required to prevent this phenotype.

3. Throughout the manuscript, the authors used three GAL4 drivers presumably allowing the expression of various RNAi transgenes in distinct cell types (NBs, all neurons, mature neurons). However, they don't clearly show where these drivers are expressed. In particular, Elav-GAL4 is known to be also expressed in NBs in the central brain. Is it also expressed in optic lobe NBs? How about its expression in the neuroepithelium? A leaky activity of this driver in the neuroepithelium could explain the small optic lobe phenotype when crossed with the Mi-2-RNAi line and provide an alternative explanation to the "non-cell autonomous" one. The "non-cell autonomous" effect could also be due to defective photoreceptor neurons from the retina, in which elav-GAL4 is active and that are known to regulate the growth and differentiation of the neuroepithelium during optic lobe development.

While elav-GAL4 can show low levels of expression in NBs, this would not explain why we see the phenotype with elav-GAL4 (Figure 4) but not with the NB wor-GAL4 driver (Figure EV2). If leaky expression in NBs was causing the phenotype, it should be observable with wor-GAL4. The C155 elav-GAL4 driver we use in this study has never been shown to be expressed in the neuroepithelium by us, or others in the field. We wondered whether leaky elav-GAL4 expression very early during specification/early life of the neuroepithelium could cause the phenotype, however, when we checked 1st instar Mi-2 knockdown brain lobes, the neuroepithelium is present (Appendix figure S10). We acknowledge that there is still a small possibility that leaky expression of the GAL4 driver line may be responsible for the phenotypes we observe. However, we would like to highlight that this does not undermine the main conclusions of our manuscript since we are using the optic lobe defect primarily as a scorable defect pointing towards coordinated action of these proteins in contrast to the ectopic gene upregulation we observe with only Mi-2 with MEP-1.

Since we see no phenotype in *wor*-GAL4 *Mi-2* knockdowns (Figure EV2), continue to see the phenotype when *repo*-GAL80 is included (Appendix figure S9), and the neuroepithelium is present in 1st instar brains (Appendix figure S10), we think that a non-cell-autonomous phenotype remains a possible explanation – indeed the photoreceptor mediated pathway that the reviewer has alerted us to is one compelling explanation. However, we have updated the text to reflect the fact that more work must be done to fully explain this phenotype.

4. The germline gene Vasa is up-regulated upon Mi-2-RNAi when using wor-GAL4 or elav-GAL4 but not nSyb-GAL4 suggesting that derepression occurs in NBs and or immature neurons. Can you validate this cell type-specific phenotype using immunostainings ? There are anti-Vasa antibodies available at DSHB.

We are primarily interested in the changes in transcript abundance which has been verified by both qPCR and RNA-seq. Since we also did not see increased vasa expression in the adult brain, we can say with confidence that fully differentiated neurons are unaffected. It is possible that vasa protein may be undetectable, due to it being at low levels (albeit many times higher than in wild-type neurons – i.e. completely absent), or due to a differing post-transcriptional environment in neurons compared to the germline. In this section, we would primarily like

to highlight that mature neurons no longer have the requirement for Mi-2 to repress these genes, in comparison to cells earlier in development. We have softened our conclusions on this point.

5. The qPCR experiments show that Vasa is also up-regulated in progenitors and immature neurons (but not mature neurons) but upon loss of Mi-2. Does this correlates with an absence or presence of Mi-2 binding on the gene in these different cell types?

We observed very different binding in elav-GAL4 compared to wor-Gal4 and nSyb-GAL4 (although Mi2 binding appears to be very similar between wor and nSyb). However, it is difficult to tell whether there is a particular site that is responsible for Mi2 regulation of the vasa locus. Using our stringent peak calling method, we did detect any significant peaks in the gene region. This is further complicated by the fact that the vasa locus is particularly complicated, containing a second nested gene within the large intron and a third gene with shared exons. However, there is at least one area with upregulated Mi-2 binding in the vas intron which is notably depleted in wor and nsyb conditions, which may be responsible for Mi2 binding leading to vasa misregulation. It is also possible that this locus could be affected by disruption of a distal enhancer or via a knock-on effect from a directly regulated gene. Indeed, it seems that this is the case for the majority of affected genes since most upregulated genes do not overlap with NuRD or dMEC binding sites (as seen in figure 3F).

6. Because inactivation of members of the NuRD complex leads to similar defects without inducing ectopic expression of germline genes, the authors conclude that "the ectopic regulation of gene is independent of NuRD/histone deacetylation, and does not contribute to optic lone developmental defects." I think this conclusion is overstated as it could be that germline genes also contribute to the phenotype (what happens if you mis-express them in neurons?). I would suggest rephrasing in something like: "The ectopic up-regulation of germline genes is not necessary for causing the optic lobe phenotype in the larva."

We agree that this is a more accurate way to describe our conclusions of this experiments and have now changed this in our updated manuscript as suggested.

7. The TaDa experiments show that Mi-2 and Mep-1 have the same binding sites therefore suggesting that they belong to the same complex. If true, their knockdown should lead to the same phenotype. What is the phenotype of Mep-1 knockdown in the optic lobe? Do you see upregulation of germline genes?

We have examined the optic lobe phenotype of MEP-1 knockdown with RNAi and seen that it does indeed phenocopy Mi-2. We observe a severe loss of neuroepithelial cells in most brains. These data have now been added to Figure 4. Furthermore, we have also performed new qPCR experiments to show that MEP-1 knockdown also results in the upregulation of the target genes also seen to be upregulated in Mi-2 knockdowns. Therefore, these data support the conclusions from our TaDa experiments regarding the presence of MEP-1 and Mi-2 in a distinct complex with unique activities.

Figures:
2B: please indicate the genotype for each track - Done.
3A: there are mistakes in the panels for Elav and Dpn - We have a new figure without these mistakes.
5: the first panel is not numbered and has no legend - Done.

The title of the panel 5B is confusing. Why is dMec mentioned here? - This has been moved to Figure 3.

The RNAi flystocks used to knock-down MBD-like are not referenced in Table S1. Only one RNAi stock for the knock-down of Mi-2 is mentioned while the authors claim to have used two lines. – All stocks are now listed in Appendix Table S1.

The "vas" abbreviation is not specified in the text - Now fixed.

Referee #2:

Summary

Aughey et al. have studied the role of the protein Mi-2 in neuronal differentiation in Drosophila. The authors show that Mi-2 protein is expressed in all stages of neurogenesis including the Neuroblasts, GMCs, and postmitotic neurons. They KD Mi-2 with the elav-Gal4 driver and revealed a decrease in fly viability and larval mobility. These phenotypes are linked to a spatio-temporal de-repression in the CNS of CNS genes and more strikingly of non-CNS genes. They also characterized the optic lobe phenotype in KD Mi-2 flies. The phenotype of the optic lobe is characterized by a reduction of its size correlated with a reduction of the number of proliferative cells (the reduction of post mitotic neurons is not clearly mentioned). The study of the function of components of the NuRD complex such as MBD-like, MTA1-like and HDAC1 revealed that the function of Mi-2 on gene repression seems independent of the NuRD complex. However the individual KD of MBD-like, MTA1-like and HDAC1 induce same optic lobe defect as Mi-2. These results suggest that Mi-2 might act through another complex to repress genes. To address this possibility, they performed TaDa on Mi-2, components of NuRD complex and MEP-1, a known protein associate of the dMEC complex that includes Mi-2. The results show that different complexes bind to the DNA, including MEP-1 and Mi-2 which have the closest similarity in their binding profile. The analysis of the binding site and the chromatin state suggest that NuRD mediates activation, while Mi2/dMEC facilitate silencing. Then Aughey et al. used different drivers to knock down Mi-2 in the NB and mature neurons and determined the impact of such manipulation on survival, optic lobe structure and gene regulation. They conclude that Mi-2 has a different function in gene regulation in NB vs immature neurons vs differentiated neurons. The comparison of Mi TaDa experiments in NBs, immature neurons vs differentiated neurons show variation on Mi-2 binding in these different types of cells that could explain the different functions of mi-2 in different cell subtypes.

In my opinion the main message of the paper that is supported by sufficient data, is that Mi2 represses the expression of several non-neuronal genes in the CNS independently of the NurD complex. This message is of broad interest.

However some conclusions put forth by the authors are not supported by strong experimental results and are in my opinion too speculative. The main conclusion proposed which must be reevaluated or supported by new experiments is:

Mi-2 has different functions (fly survival/optic lobe structure/gene regulation) in NB/GMC vs immature neurons vs immature MNs. The genetic experiments are not strong enough to support such conclusion. Below more factual arguments concerning this point and some minor points:

Related to figure 1.

Minor point: The authors claims that the mi-2::GFP is expressed in the neuroblasts, GMCs and post-mitotic neurons. The expression in NBs is convincing, since these cells can be recognized by their size. The expression in the neurons seems also convincing since many cells expressed the GFP. However it difficult to know if all post-mitotic neurons expressed mi-2::GFP and it is impossible to know if mi-2::GFP is expressed in the GMCs. If the authors want to claim that all cell types in the CNS expressed mi-2::GFP, I recommend that the authors perform co-staining with Elav, Dpn and Repo. Postmitotic neurons will be (Elav+), neuroblasts (Dpn +), glia (repo +) and GMCs (Elav -, Dpn and Repo-).

We agree that our original images could have been clearer on this point. We have produced a new image as suggested. We see clear Mi-2 expression in all Elav marked cells, as well as at low levels in some glia. It remains difficult to say whether Mi-2::GFP is present in GMCs. The text has been updated to reflect this.

Minor point: The authors also say (page 3) 'with progeny cells adjacent to NSCs exhibiting particularly high

expression'. It is true that the GFP intensity in these cells (Figure 1 B) is high compared to the neuroblast. I am curious to know why the authors make this point. The expression in the NB is weaker but this could simply be the consequence of the size of the NB (that in the figure is in the interphase phase) that can make mi-GFP protein more diluted.

We agree that this is a reasonable explanation for this observation. We have amended the text of our manuscript to remove this point.

Minor point: The authors wrote that Mi-2 activity in neurons is required throughout development at all life-stages for normal CNS function'. This sentence is a bit too 'strong' since Elav is also expressed in the PNS.

We have updated our manuscript accordingly.

Major point: The authors wrote in the title of the paragraph (page 3), that Mi-2 is required in neurons for survival and larval locomotion. The driver used, elav-gal4, has been and it is still sometime used as a neuronal postmitotic driver. However this driver is also expressed in the NB, GMCs and some glia (Berger et al., 2017). elav-gal4 can not be used to distinguish the function of Mi-2 in NBs/GMCs vs postmitotic neurons vs glia.

Whilst it is true that some expression of elav-GAL4 has been reported in non-neuronal cells, we are confident that it is suitable for our purposes here. With regards to potential NB expression – we do not see any larval locomotion phenotype (Appendix Figure S13) or optic lobe defect (Figure EV2) when using wor-GAL4 (expressed in neuroblasts), therefore we can be confident that any affects arising from *elav* knockdown are not due to unexpected knockdown in NBs. In a previous study we determined that there was no detectable effect on neuroblast divisions when targeting a cell cycle related protein with elav-GAL4 (Hassan et al, elife 2020). Therefore, we suspect that while it is prudent to be cautious of NB expression with this driver its ability to affect NB function is extremely limited.

We have also performed an experiment with repo-GAL80 to rule out the influence of glial expression (Appendix Figure S13).

Related to figure 2.
Minor points: The authors wrote we performed RNA-seq on larval brains. The sentence should be (unless I missed something in the materials and methods): we performed RNA-seq on larval CNS (which includes the VNC).

We have updated the manuscript accordingly.

Related to Figure 3.
Minor points: The DPN and Elav panel have been inverted in control vs Mi-2 RNAi. The dashed lines do not only label NBs as indicated in the figure legend. I think they indicate medulla NB and lamina neurons.

We have made a completely new figure for the optic lobe phenotype (Figure 4).

Minor point. The phenotypes induce by Mi-2 KD could be better characterized. Only the surface of the optic lobe is characterized in micrometers. The optic lobe is a 3D structure and should be measured in µm3 and not in µm2. I didn't find any information in Materials and Methods on how the authors quantified the surface. Do they quantify the surface in each focal plane of a full stack?

We have provided a more detailed analysis in which we quantify the numbers of neuroepithelial cells present in the optic lobes. We believe this quantification better reflects the extent of the observed phenotypes.

Major point: The author wrote in page 6: in Mi-2 knockdown resulted in severe optic lobe Mi-2 knockdown brains were smaller, and it was evident that all recognizable optic lobe structures (including inner and outer optic proliferation center as well as differentiating medulla and lamina neurons) were found to be completely

absent. It is difficult to see all these phenotypes in a single confocal section in figure 3. Can the authors include confocal sections showing these defects?

We have provided alternative confocal sections that better illustrate the nature of the optic lobe defect along with quantification of neuroepithelial cell numbers.

Major point: The reduction of the size of the optic lobe is linked to a reduction of the number of NB and other proliferative cells as written in the main text (page 6). This should be quantified in figure 3. Are the number of elav+ cells also reduced? It looks like the number of Elav cells is increased in all experiments of the paper using the elav-gal drivers (main figures and sup figures). This should be clarified in the text and figure 3. In summary, I recommend that the authors quantify the number of cells: NB, vs glia vs neurons)

We agree with the reviewer that our characterisation of these phenotypes could be clearer. Upon re-examining these data we realised that the preparation of the samples was causing some variation in the measurements of optic lobe area (as reported in our initial submission). As the reviewer suggested, we acquired multiple sections from which we more accurately quantified the defect. Our analysis focused on the neuroepithelial cells since these appear to be entirely/severely lost in some samples (Mi-2, MEP-1) and only partially affected in others (HDAC/MTA1-like). The difference that we see in severity of this phenotype agrees well with our main conclusion regarding the difference between NuRD and dMEC activities (although there may still be some contribution from the relative strength of the knockdowns). Since the optic lobes had the most striking phenotype, this is the part of the brain that we focused our analysis on.

Minor point: Are the phenotypes described in the optic lobe specific to the optic lobe or is it a more general effect of Mi2 KD?

We examined the VNC in the Mi-2 knockdown and there are no gross morphology changes, and the number of NB is the same as control (Appendix Figure S9).

Major point: More importantly the authors argue that the reduction in the number of NB in elav>Mi2 KD is due to a non-autonomous effect of Mi2. As written above, elav Gal4 is expressed in the NB/GMC (Berger et al., 2017). To make such conclusions the authors should use 'clean' post mitotic drivers such as drivers implicated in neurotransmitter pathway that are expressed only in postmitotic neurons and at early stage.

We would foremost like to highlight that the main conclusion we wish to draw from these experiments is that members of the NuRD complex have similar phenotypes, which provides some evidence for functioning together in the same complex in contrast to the gene expression changes which are only seen with Mi-2 knockdown. We believe that the way we structured our initial submission to put undue emphasis on the phenotypes associated with these knockdowns. In our updated manuscript we have restructured our results to highlight our main findings and have taken pains to highlight that the optic lobe phenotype is largely used as a readout for NuRD component function in this context.

Since we see no phenotype with wor-GAL4 knockdown, continue to see the phenotype with repo-GAL80, and the neuroepithelium is present in 1st instar Mi-2 brain lobes (and elav-GAL4 does not drive expression in the larval neuroepithelium), we think that a non-neuron-autonomous effect is a possible explanation. However, we accept that there are alternative explanations for this phenotype as indicated by the reviewer. We have addressed the question of unwanted NB expression in a previous comment.

Major point: The experiment with repo-gal80 is very nice to rule out a function of Mi2 in glia in the maintenance of NB. I suppose the results are described in fig S5 and not S1 (please ref correctly all sup figures, none of them are correctly annotated). Some controls in the sup figures should be added to be sure that Repo-gal80 represses elav-Gal4. Repo-gal80 might not be strong enough to repress elav-gal4 for two reasons: first repo is weakly expressed in some glia sub types such as ensheathing glia or astrocytes, second direct expression of a gene is always weaker than the UAS/GAL4 binary system. To be sure that the experiments were well designed, the

authors should check the expression of Mi2-GFP in all glia cell types in the elav>Mi2 KD repo-gal80 animals and add this control in sup Figures.

We accept that there may be residual low-level expression in some glial cells. It would be interesting to better characterise the aetiology of this phenotype and potential contribution from non-neuronal cell-types. However, the main purpose of these experiments in the context of our study was to identify similarities and differences between phenotypes arising from knockdowns of different NuRD complex members. Therefore, if there is some contribution of glial cells to the phenotype, it does not undermine our conclusions. Furthermore, we consider it unlikely that there is significant residual glial expression due to the fact that where elav is reported to be expressed in glial cells, the expression is very low and the repo-GAL80 line has been used to effectively suppress glia-linked phenotypes in several previous studies (see References at http://flybase.org/reports/FBtp0067904.html).

Minor point: The authors wrote: HDAC1 by RNAi in neurons resulted in lethality during pupal stages similarly to Mi-2. I couldn't fine any graph in the main figure or sup figure supporting this sentence.

We have now assayed pupal lethality of all the RNAi lines when crossed with elav-GAL4 (Appendix Figure S7).

Minor point: The authors claim that histone deacetylation thought HDAC1is not required for repression of Mi-2 induced ectopic gene expression in larval. I recommend the authors to be more careful in the sentence, the authors only tested few genes, to write such conclusions the authors should profile the CNS in HDAC1 RNAi.

We agree that the way we have phrased this points to a conclusion that is too broad. We have updated the manuscript to be more specific in this regard.

Minor point. Is the lethality affected in MBD-like RNAi knockdown? This should be commented on in the text. Related to Figure 4.

We have now assayed pupal lethality of all the RNAi lines when crossed with elav-GAL4 (Appendix Figure S7).

Major point: Is the function of Mi-2 in repressing genes going thought the dMEC complex (Mi-2/MEP)? An analysis of the MEP-1 knocking down and the effect on fly survival, lobe optic structure and gene expression should be done.

We have found that MEP-1 knockdown is sufficient to phenocopy the optic lobe phenotype we observed with Mi-2 knockdown (Figure 4). In support of our conclusion that MEP-1 act together in a discrete complex with Mi-2, we also observe that MEP-1 knockdown is sufficient to cause upregulation of target genes also seen to be upregulated upon Mi-2 knockdown (Figure 4). MEP-1 knockdown also resulted in pupal lethality as seen with Mi-2.

Related to Figure 4.
Major point: The authors used two drivers wor-gal4 and nsyb-gal4 to temporally define the function of Mi-2 in neuronal differentiation. I have two comments about these drivers. Wor-gal4 is expressed in all NBs but also in the young progeny, especially in the optic lobe, and cannot be considered as a specific NB marker. The graph representing the worl-gal4 expression in Figure 5 is by consequence not correct. This is critical if the authors want to conclude that Mi-2 acts in the young progeny.

We accept that there is likely to be a small amount of perdurance of wor-GAL4 in GMCs and possibly even differentiating neurons. The graph in Figure 5 and text has been adapted to reflect this.

The authors also used nSyb-Gal4 to KD Mi-2. I don't understand the goal of the experiment nor the conclusion: whilst Mi-2 is still required in mature neurons, the morphological defects observed are restricted to a critical window of early neuronal maturity. The optic lobe phenotypes described concern the neurons produced during the second wave of neurogenesis. The authors describe a phenotype in a third instar larva and at this stage

neurons are still immature and probably most if not all neurons do not express nSyb yet, so RNAi will not be expressed (at this stage it will be expressed only in the neurons produced during embryogenesis). Control experiments of wor-gal4 and nsyb-gal4 UASmcd8::GFP should be done to better define the expression of these drivers and argue against my comments if the authors want to conclude that Mi-2 has different functions at different stages of neuronal differentiation.

Major point: Moreover by using nSyb the authors are not comparing two stages of development but two different population of neurons, embryonic and larval neurons, born at different stages. If the authors want to analyze the consequence of nsyb-gal4 Mi-2 KD in the optic lobe structure the phenotype should be analysed at later stages (pupa).

A recent study has reported that in fact there are a substantial fraction of nSyb expressing neurons in the larval CNS (around 10,000 cells) (https://elifesciences.org/articles/74968). However, as we have stated in the manuscript, this is likely to be a lower number than the elav-expressing population. From our experiments we are able to say that the phenotypes that we observe do not arise from the more fully differentiated neurons that *are* present in the CNS at this stage. Since this is the developmental stage at which we observe the phenotype, we think it is valid to test for the role of nSyb neurons during this stage. These experiments were also performed in part to be able to compare with the data from Zacharioudaki et al (Elife, 2019 - Figure 3—figure supplement 1D,G), in which experiments were similarly performed in nSyb and progenitor cells and observed a similar trend. We will update the manuscript to more clearly make the distinction that these neurons are more fully differentiated, but many are not directly differentiated from the newly born elav positive neurons in the larval CNS. We agree that it is possible that a phenotype arising from nsyb neurons may manifest later in development, however we also saw no gene expression changes arising from adult specific nSyb knockdowns..

Major point: The authors used QPCR to quantify the expression of vasa in wor-gal4 and nsyb-gal4 Mi-D KD. They conclude that Mi-2 represses vasa in NBs. Again wor-gal4 is also expressed in the young progeny. smFISH to quantify vasa in the NB in wor-gal4 Mi-D KD should be done to conclude if Mi-2 plays a role in the NBs. The authors show that no detectable changes were observed in nsyb-gal4 Mi-D KD. As written before maybe it is because nSyb is not yet expressed. Maybe the authors will see changes in vasa expression at later stages when the nSyb driver is highly expressed? The decrease in vasa expression nsyb-gal4 Mi-D KD only shows that Mi-D does not regulate vasa reduction in mature neurons born during embryogenesis which could be the case of adult mature neurons.

As stated in the previous reply, there are ~ 10,000 nSyb expressing cells in the 3rd instar larval brain, therefore it is unlikely due to a lack of cells/nSyb expression causing an undetectable signal. Also, we include our qPCR data showing that there is no effect on vasa expression when Mi-2 is knocked down in adult brains (Appendix figure S12). We believe that these data effectively show that Mi-2 is not required in mature neurons for repression of the target genes examined. As the reviewer has indicated, we cannot rule out that the increased expression seen with wor-GAL4 knockdown may be due to some expression in stem cell progeny, we have altered our manuscript to soften the conclusion that Mi-2 represses gene expression in NBs.

Related to Figure 5.
Minor point: Figure 4 A, add in the figure legend (Elav, green, Dpn purple)

These figures have been changed.

Major point: I have the same comment as mentioned before for the use elav- gal4, wor-gal4 and nsyb-gal4 in the TaDa experiment. Elav-gal4 will label embryonic and larval neurons + NB/GMC/glia. wor-gal4: NBs/GMCs/ new born neurons. nsyb-gal4: embryonic born neurons. In conclusion these drivers cannot be used to obtain the temporal and spatial precision that the authors want to achieve.

In previous publications, we and others have shown that highly distinct genomic profiles can be acquired using these GAL4 lines that feature expected trends and features (for example, expression of known target genes, changes in chromatin accessibility etc…). Furthermore, even a small amount of expression in non-neuronal cells

would be very unlikely to result in significant misleading results in these data since the signal from the far more predominant neuronal cells would comprise the dominant pool of amplicons sequenced (Aughey et al 2018, eLife, Marshal et al, 2017, Nat comms).

We have also previously compared chromatin accessibility of larval and adult neurons using nSyb GAL4 and seen that they appear very similar (Aughey et al 2018), therefore, we expect that Mi-2 binding profiles in larval nSyb neurons are similar enough to adult for them to be broadly representative of differentiated neurons.

Related to the discussion
I think the discussion could be more concise and some sentence shaper : We find that in 454
contrast to the abundance of dMec, we find that around a third of Mi-2 peaks intersect with MTA1-like.

We agree that our original discussion section was too unfocused. We have made substantial edits to improve clarity and conciseness.

Referee #3:

Mi-2 (Chd4) is the catalytic subunit of the NuRD complex, a chromatin remodelling complex whose function is still not fully clear. Originally linked most strongly to gene repression, it is present at many active genes and appears to have a more modulatory role. The components are highly conserved between species, making it extremely valuable to have a detailed genomic profiling from developmental contexts. In addition, previous studies in Drosophola have identified a second Mi-2 containg complex, dMec. Although the existence of the two Mi2 containing complexes was reported previously, the roles of each are quite poorly understood. By mapping the binding sites of Mi-2 and its partners in Drosophila neural lineages, using the Dam-ID approach, Southall and co-workers shed new light on its role in these two complexes. This is the first study that compares their genome wide binding and really demonstrates that two co-exist and have different targets. This is an important contribution.

The quality of the data are very good and the study is thorough. The main criticism is that the structure of the paper is confusing and does not play to its stength- namely the DamID and more specific profiling at different stages. This could be solved with some judicious pruning and restructuring.

Comments:

1. It is valuable to have the initial overview of the phenotypes, but this could be presented in a much more condensed manner to emphasise the key points that Mi-2 (i) represses many non-neural genes (ii) appears to have NuRD dependent and independent activities.
Indeed, they could also consider reorganizing the results, to put the DamID more to the fore-- the phenotypic data would in that case substantiate those very robust results rather than vice versa. They may also be able to tie it up better with the later phenotypic analysis. This would be an advantage for the more general reader.

We agree that our original submission was not well structured and that this is likely to lead to unnecessary confusion. We have reorganised the results as the reviewer has suggested, which we think has greatly improved the clarity of our manuscript, particularly with regards to our main conclusion that Mi-2 has NuRD dependent and independent activities. Since we present the optic lobe phenotypes largely to make this point rather than to explore the exact mechanisms by which they arise, we are happy to de-emphasise these results in the narrative.

2. For the interpretations of the phenotypes in the early sections, there is also a concern that much weight is placed on Elav-Gal4 driver being expressed in neurons and not at all in the optic lobe neural stem cells (NBs) e.g. in interpreting the phenotypes as non-autonomous. They should be cautious about this-- have they double checked using a GFP or lacZ reporter that the Gal4 driver is not expressed at low levels in NBs?

While this is a concern that has been brought up by multiple reviewers, we would like to re-iterate that when we knocked down Mi-2 using a neuroblast specific driver, we observed no optic lobe phenotype, there is no effect

on larval locomotion, and no upregulation of ectopic genes (Appendix figure S14). Therefore, even if there is some low-level expression in NBs, we know that it will not produce a phenotype in this context.

3. Figure 4D would benefit from some numbers (as in 5D), it is impossible to guage what the venn diagrams mean without knowing the peak numbers in each case. It is hard to relate the legend to the pie chart in 4F, what is "other" (it would also benefit from numbers). – We have updated this figure panel (now in Figure 3).

4. They should include the phenotype form Elav-Gal4 Mep2 RNAi in comparison to the others shown in figure 3, given the overall conclusions about the 2 complexes,

As suggested, we have performed these experiments and see that MEP-1 does indeed phenocopy the Mi-2 phenotype in the optic lobe. Furthermore, MEP-1 knockdown is also sufficient to cause upregulation of the same target genes seen to be upregulated with Mi-2 neuronal knockdown. These data are now included in Figure 4.

5. it is unclear how these findings relate to the functions of mammalian Mi-2 homologue. I don't find evidence for an analogous complex in the literature, although there is evidence for CHD4 having functions outside of NuRD. It's possible that the more thorough investigation into dMec might spark interest in probing more deeply into other complexes formed by Mi-2 in other organisms. It would be useful for the paper to draw attention to this point, which it does not do at present.

We thank the reviewer for the suggestion. We have expanded on this point in our updated discussion section.

6. The discussion is currently quite long and diffuse. It could be better focussed on the major implications including the point above (5).

We accept that the discussion in our original manuscript was not as succinct as it could be. To remedy this, we have removed several paragraphs of non-critical information. In total we have reduced the length of this section by around 25% and have endeavoured to make it more concise and relevant to the presented data.

Dear Dr. Southall,

Thank you for the submission of your revised manuscript. We have now received the enclosed reports from the referees as well as cross-comments from referees 1 and 3 on referee 2's more critical report. Referees 1 and 3 do not completely agree with referee 2, and have suggestions for how these comments could be addressed. All referees thus have several suggestions that I would like you to address and incorporate before we can proceed with the official acceptance of your manuscript.

Please co-submit with your final ms a detailed point-by-point response to all referee comments below.

A few editorial requests will also need to be addressed:

- You manuscript currently has 5 main figures but separate Results and Discussion sections. Please either combine these sections and reduce the total character count to 27.000, or add one more main figure to change this into a full article. You can find more info about our article types in our guide to authors online.

- Please add up to 5 keywords to the ms file.

- Please remove the author credits from the ms file. We now use CRediT to specify the contributions of each author in the journal submission system. CRediT replaces the author contribution section. Please use the free text box to provide more detailed descriptions, if you wish. See also guide to authors https://www.embopress.org/page/journal/14693178/authorguide#authorshipguidelines.

- The REFERENCE FORMAT needs to be corrected, the names should not be in bold. The Reference section needs to be moved to just before the Figure Legends.

- Fig 5C is called out after 5F. Fig EV1D, EV2B-D, Appendix Fig S2A+B, S5B, S9A+B, S10A+B callouts are missing. Fig EV3 does not need the 'B' panel callout as there are no panels. The Appendix figure callouts need the 'S' adding, so 'Appendix Figure S#'. Please correct.

- Please delete the Appendix figure legends from the manuscript file.

- You currently have 3 EV figures and could add 2 more. But it is also fine to keep the EV figures and the Appendix as they are.

EMBO press papers are accompanied online by A) a short (1-2 sentences) summary of the findings and their significance, B) 2-3 bullet points highlighting key results and C) a synopsis image that is exactly 550 pixels wide and 200-600 pixels high (the height is variable). You can either show a model or key data in the synopsis image. Please note that text needs to be readable at the final size. Please send us this information along with the revised manuscript.

I look forward to seeing a final version of your manuscript as soon as possible. Please use this link to submit your revision: https://embor.msubmit.net/cgi-bin/main.plex

Best regards,
Esther

Esther Schnapp, PhD
Senior Editor
EMBO reports

Referee #1:

The authors have satisfactorily addressed my concerns and strongly remodelled and improved the manuscript. It convincingly shows that Mi-2 can be part of two different complexes with distinct sets of target genes during neurogenesis. In addition, it shows that Mi-2 binding is highly dynamic during this process. This study is providing important insights about how Mi-2 contributes to the regulation of developmental programs during CNS building.

Minor revisions:
Appendix FigureS2 : can you define the legend? What represent "counts" and the color-coded axis and the X-axis ?

Please give published evidence that cilia genes are enriched in testis.

Lines 283 and 285: Figure number seems wrongly attributed

Figure 5 A: there should be a blue gradient starting from NBs to depict the graded activity of elav-GAL4 already in NBs.

Referee #2:

As written in my previous review, in my opinion, the main message of the paper that is supported by sufficient data, is that Mi2 represses the expression of several non-neuronal genes in the CNS independently of the NurD complex. This message is of broad interest to the community.
But while this part of the manuscript is of interest, a main conclusion proposed by the author needs to be reevaluated or supported by new experiments. In my view, the authors cannot conclude that Mi-2 has different functions in NB/GMC vs immature neurons vs mature neurons. The author does not have sufficient evidence to conclude: Mi-2 activity is critical for ensuring maintenance of the neuronal gene expression program during maturation, but alternative mechanisms may be employed in fully differentiated neurons.
The genetic tools used in the manuscript, with the specific drivers proposed in the paper, cannot be used to obtain the temporal and spatial precision that the authors need to achieve. The temporal expression defined in fig 5A is not correct:
Wor-Gal4 is expressed in the NB, the GMC and the immature neurons. At the stage of interest these cells are NB producing adult neurons. The expression of wor-gal4 illustrating my point has been published here:
Patrick W. Johnson, Chris Q. Doe, Sen-Lin Lai,
Drosophila nucleostemin 3 is required to maintain larval neuroblast proliferation,
Developmental Biology,
Volume 440, Issue 1,
2018,
Pages 1-12,

Elav-Gal4 is expressed in the NB/GMC and some repo + cells. The author made good experiments with repo-gal80 (even if some controls should be done) to rule out the glia function. However, elav-gal4 is transiently expressed in embryonic NB and GMC. The published expression of elav-gal4 illustrating my point can be see here:
Berger C, Renner S, Lüer K, Technau GM.
The commonly used marker ELAV is transiently expressed in neuroblasts and glial cells in the Drosophila embryonic CNS.
Dev Dyn. 2007 Dec;236(12):3562-8. doi: 10.1002/dvdy.21372. PMID: 17994541.

By consequence elav-gal4 is probably also expressed in larval NB and GMC. For example, the following paper indicates that elav-Gal4 is expressed in medulla Neuroblasts:

Suzuki T, Takayama R, Sato M. eyeless/Pax6 controls the production of glial cells in the visual center of Drosophila melanogaster. Dev Biol. 2016 Jan 15;409(2):343-53. doi: 10.1016/j.ydbio.2015.12.004. Epub 2015 Dec 6. PMID: 26670857.

syb-Gal4 is expressed in mature neurons. In a third instar larva, the neurons produced by the larval NB will not express syb-Gal4 because they are immature. At this stage it is a marker of mature neurons produced during embryogenesis. The authors argue that 10000 neurons express nsyb in the CNS. This number corresponds to the number of mature larval neurons born during embryogenesis. The optic lobe is composed by neurons born after the larval stage that do not express Syb in a third instar larva. It doesn't make sense to use this driver to study morphological phenotypes in the optic lobe at this stage and the author cannot conclude that there is no role of a given gene in mature neurons because they are not yet mature when the phenotype is being analyzed. To make such conclusion, the author should analyse the phenotype at later stages or analyse the phenotype in differentiated larval neurons (expressing Syb).

In summary in a third instar larva:

Wor-Gal4 is expressed in the NB/GMC and in adult immature neurons born during larval stage
Elav-Gal4 is expressed in the NB/GMC and in larval mature neurons born during embryogenesis and in adult immature neurons.
syb-Gal4 is expressed in mature neurons born during embryogenesis

Referee #3:

With many unanswered questions still about the functional roles of Mi-2 and the NuRD complex, the work presented makes a very valuable contribution. As indicated in my original review, the genomic data make a very strong case that Mi-2 contributes to two different complexes whose target genes and roles differ. They also show that the balance in functions differs according to the step in differentiation. Altogether the data are solid and the findings very interesting. The phenotypic data helps to substantiate this major conclusion because they find different phenotypes depending on which subunits are knocked down.

The authors have followed the suggestion to reorganize the manuscript and, as a consequence, the revised manuscript brings

out the most important points more clearly and explains the molecular analysis well. The phenotypic data are now used to support the conclusion that there are different Mi-2 functional complexes and while it still remains uncertain the basis for some of the phenotypes, this is less important given the new context. The genomic data will very likely be relevant for others in the field and make the work of broad interest. Overall the standard of the work is very good and the manuscript is appropriate for publication in EMBO Reports.

The few suggestions for final revisions are primarily textual, with one very small addition of a control for knock-downs suggested:

1. They mention in the rebuttal that they the knock down for the different factors very likely differs, hence the differing severity of the effects seen (with Mi-2 always being the strongest). As they have already cDNA samples for qPCR (e.g. Fig 4A, Appendix Fig 6) they could quantify the knockdowns to verify. Admittedly this doesn't measure the residual protein, but it may well explain some of the differences and would be a valuable control.

2. They also explain in the rebuttal why they are confident about the degree of specificity from the GAL4 lines in the profiling and knock-down experiments. They should emphasise this more clearly in the text.

3. It's unclear to me why some of the data are in an Appendix rather than in Extended view figures. I have not seen this before. Is it because of the restrictions imposed by EMBO Reports? Most of the appendix figures are quite small and many could be combined into the existing Extended view figures, if additional ones are not allowed.

4. Some of the text is a bit unwieldy and/or misleading.
For example, referring to processes that do not require HDAC1 as being "independent of histone deacetylation" is not 100% accurate. The accurate statement is that they don't require HDAC1.

The following is a bit confusing: "These activities are mediated by enzymes found in two distinct subcomplexes." given the later focus on two different protein complexes. Since the next sentences refer to subunits (Mi2 and HDAC), it may be simpler to say something like "these activities are conferred by two distinct subunits" or just to omit these few sentences as they don't add much (and there are no references cited)

This sentence is a bit unclear "A complex consisting of only Mi2 and MEP-1 (termed dMec), has been suggested to be more prevalent than NuRD in fly, and that this complex is responsible for the majority of Mi-2 dependent phenotypes (Kunert et al., 2009)."

Last part of intro could be better written to fit with the revised version.

In general a careful read through of the text for typos, missing/extra words is needed.

Cross-comments from referee 1:

From what I understand, reviewer#2 is puzzled by the fact that the authors compare Mi-2 binding in neurons produced during larval stages (using wor-GAL and elav-GAL4) with neurons produced during embryogenesis (nSyb-GAL4). The problem with that, according to Reviewer#2, is that many of the neurons produced in larval stages are coming from optic lobe NBs that are absent during embryogenesis (in contrast to central brain and ventral nerve cord NBs). Therefore mature nSyb+ optic lobe neurons are absent in larvae. Hence the difference in the results obtained using wor-GAL4 and nSyb-GAL4 may not only be due to dynamics Mi-2 binding sites during neuronal maturation, but rather due to regional differences. I agree with that point, and may be a better experiment would be to compare central brain and optic lobe immature neurons in larvae (using wor-GAL4) and central brain and optic lobe mature neurons in adults (using elav-GAL4, or nSyb-GAL4).
This way, the same population of neurons would be compared over different developmental periods.

This point is valid but to me, the fact that there are different binding patterns when comparing wor-GAL4 and elav-GAL4 (that are both active in optic lobe in larvae) already suggests that maturing neurons (enriched in the elav-GAL4 condition) exhibit a dynamic Mi-2 binding pattern.
I think the authors could mitigate their conclusion with nSyb-GAL4 and mention the fact that differences compared to wor-GAL4 and elav-GAL4 may also be due to a higher proportion of optic lobe neurons in larvae compared to embryonic neurons (and therefore due to the different origin of neurons), rather than different binding patterns in mature vs immature neurons of the same origin.

Cross-comments from referee 3:

The comments made by reviewer 2 are accurate but they do not substantially detract from the findings in my opinion.

The data speak for themselves in that there are clear differences in the Mi-2 binding profiles with the different drivers (Fig 5).

This means that there are changes in its recruitment at different stages. I can comment less well on the optic lobe phenotypes, but the fact that they obtain different phenotypes does point again to the core conclusions they have reached.

The authors have justified some of these aspects in the rebuttal but they have not made these points sufficiently clear in the manuscript. My suggestion would be that they should be more accurate in explaining the drivers expression and the caveats associated with those - reviewer 1 has also highlighted the need to modify Figure 5A which is related to this point.

In doing so, they should aim to make clear that none of the drivers are "pure" in the populations they capture, and that they do overlap to some extent. Furthermore, as pointed out, by Reviewer 2, it is not certain that nsyb-gal4 will be targeting the descendants of the Larval NBs at the time they are testing (although it is not clear to me precisely when this driver does become expressed- I don't find a clear reference that says it is not expressed in the larval NB descendants at this time).

If they modify the text and the diagrams in Figure 5, they can address the point of Reviewer 2, without detracting from their main conclusions. They may also want to remove this part about the effects of nsyb-Gal4 on the optic lobe phenotype given the reviewers comments. "Next, we examined brains in which Mi-2 RNAi was driven with nSyb-GAL4 which is exclusively350 expressed in more mature synapse forming neurons. In contrast to knockdown in all neurons35111including immature neurons with elav-GAL4, depletion of Mi-2 exclusively in mature neurons did not352
produce overt morphological phenotypes and no significant difference in neuroepithelial cell numbers353
were detected when compared to controls (Figure 5B and Figure EV3B). As with the other drivers354
used, Mi-2 knockdown with nSyb-GAL4 resulted in 100 % lethality during pupal stages. Therefore,355
whilst Mi-2 is required in mature neurons, the morphological defects observed do not arise from loss356
of Mi- 2 function in fully differentiated neurons in the larval CNS."

None of these changes impact on the overall interest and quality of the work, this is a relatively minor point and relates to the final section of the paper only.

Please co-submit with your final ms a detailed point-by-point response to all referee comments below.

A few editorial requests will also need to be addressed:

- You manuscript currently has 5 main figures but separate Results and Discussion sections. Please either combine these sections and reduce the total character count to 27.000, or add one more main figure to change this into a full article. You can find more info about our article types in our guide to authors online.

- We now have 6 main figures to change to a full article.

- Please add up to 5 keywords to the ms file. – Done.

- Please remove the author credits from the ms file. We now use CRediT to specify the contributions of each author in the journal submission system. CRediT replaces the author contribution section. Please use the free text box to provide more detailed descriptions, if you wish. See also guide to authors https://www.embopress.org/page/journal/14693178/authorguide#authorshipguidelines. – Done.

- The REFERENCE FORMAT needs to be corrected, the names should not be in bold. The Reference section needs to be moved to just before the Figure Legends. – Done.

- Fig 5C is called out after 5F. Fig EV1D, EV2B-D, Appendix Fig S2A+B, S5B, S9A+B, S10A+B callouts are missing. Fig EV3 does not need the 'B' panel callout as there are no panels. The Appendix figure callouts need the 'S' adding, so 'Appendix Figure S#'. Please correct. – Done.

- Please delete the Appendix figure legends from the manuscript file. – Done.

- You currently have 3 EV figures and could add 2 more. But it is also fine to keep the EV figures and the Appendix as they are. - We have moved most of the Appendix figures into EV figures (total of 5 EV figures).

EMBO press papers are accompanied online by A) a short (1-2 sentences) summary of the findings and their significance, B) 2-3 bullet points highlighting key results and C) a synopsis image that is exactly 550 pixels wide and 200-600 pixels high (the height is variable). You can either show a model or key data in the synopsis image. Please note that text needs to be readable at the final size. Please send us this information along with the revised manuscript. – Done.

I look forward to seeing a final version of your manuscript as soon as possible. Please use this link to submit your revision: https://embor.msubmit.net/cgi-bin/main.plex

Best regards,
Esther

Esther Schnapp, PhD
Senior Editor
EMBO reports

Referee #1:

The authors have satisfactorily addressed my concerns and strongly remodelled and improved the manuscript. It convincingly shows that Mi-2 can be part of two different complexes with distinct sets of target genes during neurogenesis. In addition, it shows that Mi-2 binding is highly dynamic during this process. This

study is providing important insights about how Mi-2 contributes to the regulation of developmental programs during CNS building.

Minor revisions:
Appendix FigureS2 : can you define the legend? What represent "counts" and the color-coded axis and the X-axis ?

Done – all data, in all the GO figures, now have clear and appropriate labels (so now no need for further clarification in the legends).

Please give published evidence that cilia genes are enriched in testis.

We noticed that many of the cilia-related genes in the differentially regulated gene set were enriched in testis – this is to be expected due to the high level of ciliogenesis in the testis. However, it has proved difficult to find a primary source to corroborate this fact explicitly. Instead, we have opted to cite an appropriate review article and provide an appendix figure indicating that the genes associated with cilia-related GO terms are enriched in testis.

Lines 283 and 285: Figure number seems wrongly attributed

- figures have been re-worked and in-text references checked.

Figure 5 A: there should be a blue gradient starting from NBs to depict the graded activity of elav-GAL4 already in NBs. – Done.

Referee #2:

As written in my previous review, in my opinion, the main message of the paper that is supported by sufficient data, is that Mi2 represses the expression of several non-neuronal genes in the CNS independently of the NurD complex. This message is of broad interest to the community.
But while this part of the manuscript is of interest, a main conclusion proposed by the author needs to be reevaluated or supported by new experiments. In my view, the authors cannot conclude that Mi-2 has different functions in NB/GMC vs immature neurons vs mature neurons. The author does not have sufficient evidence to conclude: Mi-2 activity is critical for ensuring maintenance of the neuronal gene expression program during maturation, but alternative mechanisms may be employed in fully differentiated neurons. The genetic tools used in the manuscript, with the specific drivers proposed in the paper, cannot be used to obtain the temporal and spatial precision that the authors need to achieve. The temporal expression defined in fig 5A is not correct:
Wor-Gal4 is expressed in the NB, the GMC and the immature neurons. At the stage of interest these cells are NB producing adult neurons. The expression of wor-gal4 illustrating my point has been published here:
Patrick W. Johnson, Chris Q. Doe, Sen-Lin Lai,
Drosophila nucleostemin 3 is required to maintain larval neuroblast proliferation,
Developmental Biology,
Volume 440, Issue 1,
2018,
Pages 1-12,

We acknowledge that *wor*-GAL4 perdures into progeny cells. We had attempted to demonstrate this with the fading grey region in our figure, but this was not clear enough. We have amended the figure accordingly.

While this driver does not express perfectly in NBs only, we are unsure why the reviewer thinks that it is unsuitable for our purposes. We initially use this driver to rule out the contribution of proliferating NBs and GMCs to the optic lobe phenotype. Since the driver is expressed in these cells as demonstrated by Johnson et al., this is an appropriate driver to use for this purpose. We have added further caveats to our text to reflect the specificity of *wor*-GAL4.

We next use *wor*-GAL4 to profile Mi-2 binding in NSCs. As we have previously stated, this *wor*-GAL4 has been used previously by ourselves and others for this purpose. In these instances it is possible to see clear and expected differences in the profiles obtained between *wor*-GAL4 and drivers that express in the GMCs/early neurons (Marshall and Brand, 2017 PMID: 29273756 and Aughey et al., 2018 PMID: 29481322). We expect that the perdurance is less of an issue in the context of a Targeted DamID experiment due to the fact that the Dam-fusion protein is expressed at extremely low levels, therefore it is likely to be diluted out more effectively following mitosis unlike the very highly expressed reporters that are normally visualised. Of course, it is likely that GAL4 is also still present in the progeny cells, so there will be remaining expression of the Dam-fusion – however, as previously stated this has been shown to not be a major problem. We have further amended the text to re-iterate that these profiles may represent NBs and some progeny, but we do not believe that this changes any of our conclusions regarding these experiments.

Elav-Gal4 is expressed in the NB/GMC and some repo + cells. The author made good experiments with repo-gal80 (even if some controls should be done) to rule out the glia function. However, elav-gal4 is transiently expressed in embryonic NB and GMC. The published expression of elav-gal4 illustrating my point can be see here:
Berger C, Renner S, Lüer K, Technau GM.
The commonly used marker ELAV is transiently expressed in neuroblasts and glial cells in the Drosophila embryonic CNS.
Dev Dyn. 2007 Dec;236(12):3562-8. doi: 10.1002/dvdy.21372. PMID: 17994541.

By consequence elav-gal4 is probably also expressed in larval NB and GMC. For example, the following paper indicates that elav-Gal4 is expressed in medulla Neuroblasts:

Suzuki T, Takayama R, Sato M. eyeless/Pax6 controls the production of glial cells in the visual center of Drosophila melanogaster. Dev Biol. 2016 Jan 15;409(2):343-53. doi: 10.1016/j.ydbio.2015.12.004. Epub 2015 Dec 6. PMID: 26670857.

We have agreed with the reviewer previously that *elav*-GAL4 may be expressed in other non-neuronal cells. We will further update the text in our manuscript to reflect this fact. However, we are again unsure of how the reviewer thinks this expression might undermine our conclusions. We have used *wor*-GAL4 (which is expressed in the medulla neuroblasts), to rule out a contribution of unexpected NB knockdown to the optic lobe phenotype.

For the Targeted DamID experiments, even if *elav*-GAL4 is expressed in some non-neuronal cells, these are far outnumbered by neurons meaning the majority of the signal will be derived from these cells. We have further updated the manuscript to emphasise that the profiles presented may reflect this, but we do not think this changes our overall conclusions that there are dynamic changes in Mi-2 binding between neuronal precursor cells and neurons.

syb-Gal4 is expressed in mature neurons. In a third instar larva, the neurons produced by the larval NB will not express syb-Gal4 because they are immature. At this stage it is a marker of mature neurons produced during embryogenesis. The authors argue that 10000 neurons express nsyb in the CNS. This number corresponds to the number of mature larval neurons born during embryogenesis. The optic lobe is

composed by neurons born after the larval stage that do not express Syb in a third instar larva. It doesn't make sense to use this driver to study morphological phenotypes in the optic lobe at this stage and the author cannot conclude that there is no role of a given gene in mature neurons because they are not yet mature when the phenotype is being analyzed. To make such conclusion, the author should analyse the phenotype at later stages or analyse the phenotype in differentiated larval neurons (expressing Syb).

We understand that the nSyb -expressing neurons in the larval CNS represent a different population of cells. It seems that the logic of these experiments is not entirely clear, so it may help to expand on this, both here and in the manuscript. Having seen changes in gene expression using both *wor*-GAL4 and *nSyb*-GAL4, we were interested in seeing whether this ectopic gene expression could also be seen in more fully differentiated cells which are included in the *elav*-GAL4 knockdown. It was not possible to do these experiments in later stages because they do not survive long enough. Therefore, we looked at the complement of fully differentiated neurons that were available in the larval brain to look at this –and saw no changes in gene expression, (and as previously discussed, we also used an inducible system and saw the same result in adult neurons). Having used this driver in the context of the larval CNS, it seemed appropriate to us to also check whether an optic lobe phenotype was also present. We did this not because these cells are necessarily produced at this stage, but simply because they are a population of cells that are present in the larval CNS and we thought to rule out that there was a small chance that they may be producing signals acting non-cell-autonomously on the optic lobe. Although this is not a particularly surprising result, we would rather include these data than not. We would like to emphasise that we do not conclude that there is no role for Mi-2 in mature neurons (Since we show that it causes lethality), we merely wished to say that at the point that we see the phenotype, the neurons that are present at that stage (and represent a proportion of the cells included in the *elav*-GAL4 experiments) are not contributing to its aetiology.

We are a little confused about the reviewer's assertion that we cannot draw conclusions about the contribution of mature neurons to this phenotype without looking at later stages, since these neurons do not yet exist at the stage when the phenotype is apparent (as the reviewer has rightly pointed out). It is of course possible that mature neurons derived from the larval stages of neurogenesis contribute separately to an optic lobe phenotype later in the animal, however, since they are not yet present in the animals we are looking at, we conclude that they are not involved in the larval optic lobe phenotype.

We have re-organised our manuscript again to emphasise what we think is our main result – that *nSyb*-GAL4 does not produce any upregulation of *vasa* as is seen when knocked down in either NBs or larval born neurons.

The final instance in which we use *nSyb*-GAL4 is to determine the binding of Mi-2 in mature neurons. We decided it would be better to look at the nSyb neurons in the larval CNS rather than the adult because although they are derived from the embryo rather than the larvae they still represent a population of fully differentiated neurons in which we had seen a different response the knockdown of Mi-2. We accept that some of the changes in Mi-2 binding may be due to lineage specific differences rather than due to developmental stage and we have updated the manuscript to further reflect this point.

In summary in a third instar larva:

Wor-Gal4 is expressed in the NB/GMC and in adult immature neurons born during larval stage
Elav-Gal4 is expressed in the NB/GMC and in larval mature neurons born during embryogenesis and in adult immature neurons.
syb-Gal4 is expressed in mature neurons born during embryogenesis

Referee #3:

With many unanswered questions still about the functional roles of Mi-2 and the NuRD complex, the work presented makes a very valuable contribution. As indicated in my original review, the genomic data make a very strong case that Mi-2 contributes to two different complexes whose target genes and roles differ. They also show that the balance in functions differs according to the step in differentiation. Altogether the data are solid and the findings very interesting. The phenotypic data helps to substantiate this major conclusion because they find different phenotypes depending on which subunits are knocked down.

The authors have followed the suggestion to reorganize the manuscript and, as a consequence, the revised manuscript brings out the most important points more clearly and explains the molecular analysis well. The phenotypic data are now used to support the conclusion that there are different Mi-2 functional complexes and while it still remains uncertain the basis for some of the phenotypes, this is less important given the new context. The genomic data will very likely be relevant for others in the field and make the work of broad interest. Overall the standard of the work is very good and the manuscript is appropriate for publication in EMBO Reports.

The few suggestions for final revisions are primarily textual, with one very small addition of a control for knock-downs suggested:

1. They mention in the rebuttal that they the knock down for the different factors very likely differs, hence the differing severity of the effects seen (with Mi-2 always being the strongest). As they have already cDNA samples for qPCR (e.g. Fig 4A, Appendix Fig 6) they could quantify the knockdowns to verify. Admittedly this doesn't measure the residual protein, but it may well explain some of the differences and would be a valuable control.

We agree that these experiments would help us to understand the differences in phenotypes that we observe. However, these experiments are not trivial to perform. Since we do not have spare cDNA we would have to set up new crosses and dissect larvae (which would amount to hundreds of animals for the required replicates for every genotype). This would be especially challenging as we currently lack the personnel to perform such experiments. Since this would result in a further delay of at least one month we would prefer not to expend so much effort on a relatively minor point. As previously discussed, the fact that we are able to see similar phenotypes with multiple different complex members is strong evidence to conclude that NuRD as a whole is functioning in this context. We have added a line in the text to indicate that there remains some question as to the level of contribution of each complex to the optic lobe phenotype.

2. They also explain in the rebuttal why they are confident about the degree of specificity from the GAL4 lines in the profiling and knock-down experiments. They should emphasise this more clearly in the text. – Done.

3. It's unclear to me why some of the data are in an Appendix rather than in Extended view figures. I have not seen this before. Is it because of the restrictions imposed by EMBO Reports? Most of the appendix figures are quite small and many could be combined into the existing Extended view figures, if additional ones are not allowed. – We have incorporated the majority of the Appendix figures into EV figures.

4. Some of the text is a bit unwieldy and/or misleading.
For example, referring to processes that do not require HDAC1 as being "independent of histone deacetylation" is not 100% accurate. The accurate statement is that they don't require HDAC1. – Done.

The following is a bit confusing: "These activities are mediated by enzymes found in two distinct subcomplexes." given the later focus on two different protein complexes. Since the next sentences refer to

subunits (Mi2 and HDAC), it may be simpler to say something like "these activities are conferred by two distinct subunits" or just to omit these few sentences as they don't add much (and there are no references cited) – Done.

This sentence is a bit unclear "A complex consisting of only Mi2 and MEP-1 (termed dMec), has been suggested to be more prevalent than NuRD in fly, and that this complex is responsible for the majority of Mi-2 dependent phenotypes (Kunert et al., 2009)." – Done.

Last part of intro could be better written to fit with the revised version. – Done.

In general a careful read through of the text for typos, missing/extra words is needed. – Done.

Cross-comments from referee 1:

From what I understand, reviewer#2 is puzzled by the fact that the authors compare Mi-2 binding in neurons produced during larval stages (using wor-GAL and elav-GAL4) with neurons produced during embryogenesis (nSyb-GAL4). The problem with that, according to Reviewer#2, is that many of the neurons produced in larval stages are coming from optic lobe NBs that are absent during embryogenesis (in contrast to central brain and ventral nerve cord NBs). Therefore mature nSyb+ optic lobe neurons are absent in larvae. Hence the difference in the results obtained using wor-GAL4 and nSyb-GAL4 may not only be due to dynamics Mi-2 binding sites during neuronal maturation, but rather due to regional differences. I agree with that point, and may be a better experiment would be to compare central brain and optic lobe immature neurons in larvae (using wor-GAL4) and central brain and optic lobe mature neurons in adults (using elav-GAL4, or nSyb-GAL4). This way, the same population of neurons would be compared over different developmental periods.

This point is valid but to me, the fact that there are different binding patterns when comparing wor-GAL4 and elav-GAL4 (that are both active in optic lobe in larvae) already suggests that maturing neurons (enriched in the elav-GAL4 condition) exhibit a dynamic Mi-2 binding pattern.
I think the authors could mitigate their conclusion with nSyb-GAL4 and mention the fact that differences compared to wor-GAL4 and elav-GAL4 may also be due to a higher proportion of optic lobe neurons in larvae compared to embryonic neurons (and therefore due to the different origin of neurons), rather than different binding patterns in mature vs immature neurons of the same origin.

We agree that this is a valid point, and have updated our manuscript to include this caveat. However, since we see examples of changes that fit with known roles of Mi-2 in neurogenesis (e.g. striking loss of Mi-2 binding at the *E(spl)* locus), we have some confidence that the changes we see are primarily due to differentiation stage rather than lineage. While comparing to adult neurons may have given a better indication of developmental changes, we have less idea of the phenotypes at this stage, so ultimately, we think the larval *nSyb*-GAL4 driven data provides a more coherent picture.

Cross-comments from referee 3:

The comments made by reviewer 2 are accurate but they do not substantially detract from the findings in my opinion.

The data speak for themselves in that there are clear differences in the Mi-2 binding profiles with the different drivers (Fig 5). This means that there are changes in its recruitment at different stages. I can comment less well on the optic lobe phenotypes, but the fact that they obtain different phenotypes does

point again to the core conclusions they have reached.

The authors have justified some of these aspects in the rebuttal but they have not made these points sufficiently clear in the manuscript. My suggestion would be that they should be more accurate in explaining the drivers expression and the caveats associated with those - reviewer 1 has also highlighted the need to modify Figure 5A which is related to this point.

In doing so, they should aim to make clear that none of the drivers are "pure" in the populations they capture, and that they do overlap to some extent. Furthermore, as pointed out, by Reviewer 2, it is not certain that nsyb-gal4 will be targeting the descendants of the Larval NBs at the time they are testing (although it is not clear to me precisely when this driver does become expressed- I don't find a clear reference that says it is not expressed in the larval NB descendants at this time).

If they modify the text and the diagrams in Figure 5, they can address the point of Reviewer 2, without detracting from their main conclusions. They may also want to remove this part about the effects of nsyb-Gal4 on the optic lobe phenotype given the reviewers comments. "Next, we examined brains in which Mi-2 RNAi was driven with nSyb-GAL4 which is exclusively350
expressed in more mature synapse forming neurons. In contrast to knockdown in all neurons35111including immature neurons with elav-GAL4, depletion of Mi-2 exclusively in mature neurons did not352
produce overt morphological phenotypes and no significant difference in neuroepithelial cell numbers353
were detected when compared to controls (Figure 5B and Figure EV3B). As with the other drivers354
used, Mi-2 knockdown with nSyb-GAL4 resulted in 100 % lethality during pupal stages. Therefore,355
whilst Mi-2 is required in mature neurons, the morphological defects observed do not arise from loss356
of Mi- 2 function in fully differentiated neurons in the larval CNS."

None of these changes impact on the overall interest and quality of the work, this is a relatively minor point and relates to the final section of the paper only.

As the reviewer has suggested we have further updated our manuscript to further emphasise the caveats regarding the drivers utilised. We have further explained our rationale for including certain experiments in the text.

2nd Revision - Editorial Decision 16th Jan 2023

Dr. Tony Southall
Imperial College London
Life Sciences
Sir Ernst Chain Building
Imperial College Road
London SW7 2AZ
United Kingdom

Dear Dr. Southall,

I am very pleased to accept your manuscript for publication in the next available issue of EMBO reports. Thank you for your contribution to our journal.

At the end of this email I include important information about how to proceed. Please ensure that you take the time to read the information and complete and return the necessary forms to allow us to publish your manuscript as quickly as possible.

As part of the EMBO publication's Transparent Editorial Process, EMBO reports publishes online a Review Process File to accompany accepted manuscripts. As you are aware, this File will be published in conjunction with your paper and will include the referee reports, your point-by-point response and all pertinent correspondence relating to the manuscript.

If you do NOT want this File to be published, please inform the editorial office within 2 days, if you have not done so already, otherwise the File will be published by default [contact: emboreports@embo.org]. If you do opt out, the Review Process File link will point to the following statement: "No Review Process File is available with this article, as the authors have chosen not to make the review process public in this case."

Thank you again for your contribution to EMBO reports and congratulations on a successful publication. Please consider us again in the future for your most exciting work.

Yours sincerely,

Esther Schnapp, PhD
Senior Editor
EMBO reports

****

THINGS TO DO NOW:

Please note that you will be contacted by Wiley Author Services to complete licensing and payment information. The required 'Page Charges Authorization Form' is available here: https://www.embopress.org/pb-assets/embo-site/er_apc.pdf - please download and complete the form and return to embopressproduction@wiley.com

EMBO Press participates in many Publish and Read agreements that allow authors to publish Open Access with reduced/no publication charges. Check your eligibility: https://authorservices.wiley.com/author-resources/Journal-Authors/open-access/affiliation-policies-payments/index.html

You will receive proofs by e-mail approximately 2-3 weeks after all relevant files have been sent to our Production Office; you should return your corrections within 2 days of receiving the proofs.

Please inform us if there is likely to be any difficulty in reaching you at the above address at that time. Failure to meet our deadlines may result in a delay of publication, or publication without your corrections.

All further communications concerning your paper should quote reference number EMBOR-2022-55362V3 and be addressed to emboreports@wiley.com.

Should you be planning a Press Release on your article, please get in contact with emboreports@wiley.com as early as possible, in order to coordinate publication and release dates.

EMBO Press Author Checklist

Corresponding Author Name: Tony Southall	
Journal Submitted to: EMBO Reports	
Manuscript Number: EMBOR-2022-55362V2	

USEFUL LINKS FOR COMPLETING THIS FORM
The EMBO Journal - Author Guidelines
EMBO Reports - Author Guidelines
Molecular Systems Biology - Author Guidelines
EMBO Molecular Medicine - Author Guidelines

Reporting Checklist for Life Science Articles (updated January 2022)
This checklist is adapted from Materials Design Analysis Reporting (MDAR) Checklist for Authors. MDAR establishes a minimum set of requirements in transparent reporting in the life sciences (see Statement of Task: 10.31222/osf.io/9sm4x). Please follow the journal's guidelines in preparing your manuscript.
Please note that a copy of this checklist will be published alongside your article.

Abridged guidelines for figures
1. Data
The data shown in figures should satisfy the following conditions:
- the data were obtained and processed according to the field's best practice and are presented to reflect the results of the experiments in an accurate and unbiased manner.
- ideally, figure panels should include only measurements that are directly comparable to each other and obtained with the same assay.
- plots include clearly labeled error bars for independent experiments and sample sizes. Unless justified, error bars should not be shown for technical replicates.
- if n<5, the individual data points from each experiment should be plotted. Any statistical test employed should be justified.
- Source Data should be included to report the data underlying figures according to the guidelines set out in the authorship guidelines on Data Presentation.

2. Captions
Each figure caption should contain the following information, for each panel where they are relevant:
- a specification of the experimental system investigated (eg cell line, species name).
- the assay(s) and method(s) used to carry out the reported observations and measurements.
- an explicit mention of the biological and chemical entity(ies) that are being measured.
- an explicit mention of the biological and chemical entity(ies) that are altered/varied/perturbed in a controlled manner.
- the exact sample size (n) for each experimental group/condition, given as a number, not a range;
- a description of the sample collection allowing the reader to understand whether the samples represent technical or biological replicates (including how many animals, litters, cultures, etc.).
- a statement of how many times the experiment shown was independently replicated in the laboratory.
- definitions of statistical methods and measures:
  - common tests, such as t-test (please specify whether paired vs. unpaired), simple χ2 tests, Wilcoxon and Mann-Whitney tests, can be unambiguously identified by name only, but more complex techniques should be described in the methods section;
  - are tests one-sided or two-sided?
  - are there adjustments for multiple comparisons?
  - exact statistical test results, e.g., P values = x but not P values < x;
  - definition of 'center values' as median or average;
  - definition of error bars as s.d. or s.e.m.

Please complete ALL of the questions below.
Select "Not Applicable" only when the requested information is not relevant for your study.

Materials

Newly Created Materials	Information included in the manuscript?	In which section is the information available? (Reagents and Tools Table, Materials and Methods, Figures, Data Availability Section)
New materials and reagents need to be available; do any restrictions apply?	Yes	Materials and Methods

Antibodies	Information included in the manuscript?	In which section is the information available? (Reagents and Tools Table, Materials and Methods, Figures, Data Availability Section)
For **antibodies** provide the following information:<br>- Commercial antibodies: RRID (if possible) or supplier name, catalogue number and or/clone number<br>- Non-commercial: RRID or citation	Yes	Materials and Methods

DNA and RNA sequences	Information included in the manuscript?	In which section is the information available? (Reagents and Tools Table, Materials and Methods, Figures, Data Availability Section)
**Short novel DNA or RNA including primers, probes**: provide the sequences.	Yes	Materials and Methods

Cell materials	Information included in the manuscript?	In which section is the information available? (Reagents and Tools Table, Materials and Methods, Figures, Data Availability Section)
**Cell lines:** Provide species information, strain. Provide accession number in repository **OR** supplier name, catalog number, clone number, and/**OR** RRID.	Not Applicable	
**Primary cultures:** Provide species, strain, sex of origin, genetic modification status.	Not Applicable	
Report if the cell lines were recently **authenticated** (e.g., by STR profiling) and tested for mycoplasma contamination.	Not Applicable	

Experimental animals	Information included in the manuscript?	In which section is the information available? (Reagents and Tools Table, Materials and Methods, Figures, Data Availability Section)
**Laboratory animals or Model organisms:** Provide species, strain, sex, age, genetic modification status. Provide accession number in repository **OR** supplier name, catalog number, clone number, **OR** RRID.	Yes	Materials and Methods and Appendix Table S1
**Animal observed in or captured from the field:** Provide species, sex, and age where possible.	Not Applicable	
Please detail **housing and husbandry conditions**.	Yes	Materials and Methods

Plants and microbes	Information included in the manuscript?	In which section is the information available? (Reagents and Tools Table, Materials and Methods, Figures, Data Availability Section)
**Plants:** provide species and strain, ecotype and cultivar where relevant, unique accession number if available, and source (including location for collected wild specimens).	Not Applicable	
**Microbes:** provide species and strain, unique accession number if available, and source.	Not Applicable	

Human research participants	Information included in the manuscript?	In which section is the information available? (Reagents and Tools Table, Materials and Methods, Figures, Data Availability Section)
If collected and within the bounds of privacy constraints report on age, sex and gender or ethnicity for all study participants.	Not Applicable	

Core facilities	Information included in the manuscript?	In which section is the information available? (Reagents and Tools Table, Materials and Methods, Figures, Data Availability Section)
If your work benefited from core facilities, was their service mentioned in the acknowledgments section?	Not Applicable	

Design

Study protocol	Information included in the manuscript?	In which section is the information available? (Reagents and Tools Table, Materials and Methods, Figures, Data Availability Section)
If study protocol has been **pre-registered, provide DOI in the manuscript**. For clinical trials, provide the trial registration number **OR** cite DOI.	Not Applicable	
Report the **clinical trial registration number** (at ClinicalTrials.gov or equivalent), where applicable.	Not Applicable	

Laboratory protocol	Information included in the manuscript?	In which section is the information available? (Reagents and Tools Table, Materials and Methods, Figures, Data Availability Section)
Provide DOI OR other citation details if **external detailed step-by-step protocols** are available.	Yes	Material and Methods and reference cited in the main text

Experimental study design and statistics	Information included in the manuscript?	In which section is the information available? (Reagents and Tools Table, Materials and Methods, Figures, Data Availability Section)
Include a statement about **sample size** estimate even if no statistical methods were used.	Yes	Figure legends
Were any steps taken to minimize the effects of subjective bias when allocating animals/samples to treatment (e.g. **randomization procedure**)? If yes, have they been described?	Not Applicable	
Include a statement about **blinding** even if no blinding was done.	Yes	No blinding was done
Describe **inclusion/exclusion criteria** if samples or animals were excluded from the analysis. Were the criteria pre-established?<br><br>If sample or data points were omitted from analysis, report if this was due to attrition or intentional exclusion and provide justification.	Not Applicable	
For every figure, are **statistical tests** justified as appropriate? Do the data meet the assumptions of the tests (e.g., normal distribution)? Describe any methods used to assess it. Is there an estimate of variation within each group of data? Is the variance similar between the groups that are being statistically compared?	Yes	Figure legends / Materials and methods

Sample definition and in-laboratory replication	Information included in the manuscript?	In which section is the information available? (Reagents and Tools Table, Materials and Methods, Figures, Data Availability Section)
In the figure legends: state number of times the experiment was **replicated** in laboratory.	Yes	Figures
In the figure legends: define whether data describe **technical or biological replicates**.	Yes	Figures

Ethics

Ethics	Information included in the manuscript?	In which section is the information available? (Reagents and Tools Table, Materials and Methods, Figures, Data Availability Section)
Studies involving **human participants**: State details of **authority granting ethics approval** (IRB or equivalent committee(s), provide reference number for approval.	Not Applicable	
Studies involving **human participants**: Include a statement confirming that **informed consent** was obtained from all subjects and that the experiments conformed to the principles set out in the WMA Declaration of Helsinki and the Department of Health and Human Services Belmont Report.	Not Applicable	
Studies involving **human participants:** For publication of **patient photos**, include a statement confirming that consent to publish was obtained.	Not Applicable	
Studies involving experimental **animals**: State details of **authority granting ethics approva**l (IRB or equivalent committee(s), provide reference number for approval. Include a statement of compliance with ethical regulations.	Not Applicable	
Studies involving **specimen and field samples:** State if relevant **permits** obtained, provide details of authority approving study; if none were required, explain why.	Not Applicable	

Dual Use Research of Concern (DURC)	Information included in the manuscript?	In which section is the information available? (Reagents and Tools Table, Materials and Methods, Figures, Data Availability Section)
Could your study fall under dual use research restrictions? Please check biosecurity documents and list of **select agents and toxins** (CDC): https://www.selectagents.gov/sat/list.htm	Not Applicable	
If you used a select agent, is the security level of the lab appropriate and reported in the manuscript?	Not Applicable	
If a study is subject to dual use research of concern regulations, is the name of the **authority granting approval and reference number** for the regulatory approval provided in the manuscript?	Not Applicable	

Reporting

The MDAR framework recommends adoption of discipline-specific guidelines, established and endorsed through community initiatives. Journals have their own policy about requiring specific guidelines and recommendations to complement MDAR.

Adherence to community standards	Information included in the manuscript?	In which section is the information available? (Reagents and Tools Table, Materials and Methods, Figures, Data Availability Section)
State if relevant guidelines or checklists (e.g., **ICMJE, MIBBI, ARRIVE, PRISMA**) have been followed or provided.	Not Applicable	
For **tumor marker prognostic studies,** we recommend that you follow the **REMARK** reporting guidelines (see link list at top right). See author guidelines, under 'Reporting Guidelines'. Please confirm you have followed these guidelines.	Not Applicable	
For **phase II and III randomized controlled trials**, please refer to the **CONSORT** flow diagram (see link list at top right) and submit the CONSORT checklist (see link list at top right) with your submission. See author guidelines, under 'Reporting Guidelines'. Please confirm you have submitted this list.	Not Applicable	

Data Availability

Data availability	Information included in the manuscript?	In which section is the information available? (Reagents and Tools Table, Materials and Methods, Figures, Data Availability Section)
Have **primary datasets** been deposited according to the journal's guidelines (see 'Data Deposition' section) and the respective accession numbers provided in the Data Availability Section?	Yes	Material and Methods
Were **human clinical and genomic datasets** deposited in a public access-controlled repository in accordance to ethical obligations to the patients and to the applicable consent agreement?	Not Applicable	
Are **computational models** that are central and integral to a study available without restrictions in a machine-readable form? Were the relevant accession numbers or links provided?	Not Applicable	
If publicly available data were reused, provide the respective **data citations in the reference list**.	Yes	Main text and reference list
